# Mitochondrial DNA drives NLRP3-IL-1β axis activation in microglia by binding to NLRP3, leading to neurodegeneration in Parkinson’s disease models

**DOI:** 10.1038/s41419-026-08424-7

**Published:** 2026-02-10

**Authors:** Qinglin Gan, Xiaolong Fu, Ting Zhou, Naiyu Fan, Nan Nan, Yi Wang, Yonggang Yang, Shiyi Gou, Lizhen Hu, Shaoyu Zhou

**Affiliations:** 1 Key Laboratory of Basic Pharmacology of Ministry of Education and Joint International Research Laboratory of Ethnomedicine of Ministry of Education, Zunyi Medical University, Zunyi, Guizhou China; 2 Jinsha County People’s Hospital, Bijie, Guizhou China

**Keywords:** Parkinson's disease, Neurodegeneration, Microglia

## Abstract

Dysregulated mitochondrial DNA (mtDNA) promotes inflammatory response and disease progression. However, the mechanism and role of mtDNA-mediated inflammatory activation in the pathogenesis of Parkinson’s disease (PD) are not yet clear. This study demonstrates that the injection of mtDNA into the substantia nigra pars compacta induces PD pathology in mice, characterized by the loss of dopaminergic (DA) neurons and the activation of microglia. Transcriptomic profiling of magnetic-activated cell sorting (MACS)-sorted cells reveals a pronounced upregulation of genes associated with the NLRP3 inflammasome pathway in microglia following the mtDNA administration. Critically, lipopolysaccharide (LPS) and rotenone induced in vivo and in vitro PD models show oxidized mtDNA (ox-mtDNA) release and microglial NLRP3-IL-1β axis activation as evidenced by upregulation of NLRP3 and IL-1β, caspase-1 cleavage, and IL-1β release. The role of mtDNA in activating the NLRP3-IL-1β axis is further validated in BV2 cells through exogeneous mtDNA transfection, while the NLRP3-IL-1β activation is negated in the LPS and rotenone induced model when mtDNA release is inhibited. Especially, oxidized mtDNA is superior to nonoxidized mtDNA in activating the NLRP3-IL-1β axis. NLRP3 knockdown in BV2 cells abolishes the activation of NLRP3-IL-1β axis induced by mtDNA or exposure of LPS and rotenone and mitigates the damage to SH-SY5Y cells in co-culture systems. Ox-mtDNA-mediated neuronal cell damage is initiated through binding to NLRP3, as demonstrated by co-immunoprecipitation and co-localization in BV2 cells. Molecular docking prediction and analysis of intrinsically disordered region (IDR) of NLRP3 indicate that ox-mtDNA interacts with the positively charged IDR of NLRP3. This interaction is validated by electrophoretic mobility shift and in vitro PYD-caspase-1 cleavage assays, demonstrating the formation of the ox-mtDNA-NLRP3 complex and subsequent activation of NLRP3. This study describes a critical role of mtDNA in activating microglial NLRP3-IL-1β axis, leading to neurodegeneration in PD pathology, which provides clear clues for developing anti-PD drugs targeting NLRP3.

## Introduction

Parkinson’s disease (PD), the second most prevalent neurodegenerative disorder, is characterized by the progressive loss of dopaminergic (DA) neurons in the substantia nigra pars compacta (SNpc) and motor dysfunction [1]. Recent evidence highlights microglial overactivation as a crucial neuroinflammation driver associated with neuronal death [2, 3]. Activated microglia facilitate the progression of PD through the overproduction of reactive oxygen species (ROS) and the release of pro-inflammatory cytokines [4]. However, the molecular events linking microglial activation to the loss of DA neurons remain elusive.

Mitochondria play a central role in cellular energy metabolism through ATP production, and their dysfunction has been extensively studied, which is associated with neuronal loss leading to PD [5]. Especially, mitochondrial complex I (CI) impairment in DA neurons has long been recognized as a hallmark of PD. Yet, new research demonstrated that mice with genetic disruption of mitochondrial CI in DA neurons do not exhibit PD’s characteristic severe movement impairments, which occur only in older mice with DA signaling deficiencies spreading over both striatum and SNpc [6]. In addition, a recent study of brain tissues found that PD patients exhibit two distinct subtypes, widespread neuronal CI deficiency and non-CI deficient subtype, with the non-CI deficient subtype only showing mitochondrial impairment in the DA SNpc [7]. These studies do not deny the contribution of mitochondrial dysfunction to the pathological progression of PD, but rather unravel the heterogeneous pathophysiology and complexity of mitochondrial dysfunction in the pathological mechanism of PD. Therefore, further research on the molecular mechanisms of mitochondrial dysfunction, particularly in-depth exploration of extra-neuron mitochondrial dysfunction, is crucial for understanding PD’s pathological progression and therapeutic strategies.

Increasing studies suggest that microglial hyperactivation is related to mitochondrial dysfunction and oxidative stress [8]. Mitochondrial DNA (mtDNA) exhibits immunostimulatory capacity due to its bacterial endosymbiotic heritage combined with acquired features like oxidative damage and mutagenic signatures [9, 10]. These unique features enable mtDNA to function as a damage-associated molecular pattern (DAMP), leading to neuroinflammation. Many mitochondrial components and metabolites can act as DAMPs and promote inflammation when released into the cytoplasm or extracellular environment [11]. Among mitochondrial DAMPs, mtDNA has been identified as a potent activator of microglial pro-inflammatory pathways, including TLR9 and cGAS-STING signaling [12, 13]. Notably, mitochondrial dysfunction is often accompanied by oxidative stress, which generates large amounts of ROS, leading to the oxidation of mtDNA. Zhong et al. showed that oxidized mtDNA (ox-mtDNA), compared to unoxidized mtDNA, preferentially activates the inflammasome sensor NLRP3 [14]. A study also showed that mtDNA must first be oxidized to activate NLRP3 [15]. Despite these studies, the exact molecular mechanism of ox-mtDNA-mediated microglial activation and its downstream signaling to neurodegeneration in the pathogenesis of PD has not been fully determined.

Ox-mtDNA activates multiple inflammatory pathways in microglia, with the NLRP3 inflammasome emerging as a central mediator [16, 17]. It has been demonstrated that inhibiting mtDNA release or oxidation mitigates neuroinflammation and the loss of DA neurons [18, 19]. Upon priming via NF-κB signaling, NLRP3 recruits ASC and pro-caspase-1 to form the inflammasome complex, which catalyzes the autoactivation of caspase-1. Active caspase-1 cleaves pro-IL-1β into mature IL-1β, a potent neurotoxic cytokine [20]. Elevated plasma NLRP3 expression and IL-1β levels had been observed in individuals with PD [21]. Additionally, preclinical studies demonstrate that either NLRP3 knockout or pharmacological inhibition attenuates DA neuron loss in MPTP and α-synuclein models [22]. Despite these advances, the precise mechanism by which ox-mtDNA engages NLRP3 to initiate inflammasome assembly and neurodegeneration remains unclear in PD pathology, thereby limiting potential therapeutic targeting.

This study demonstrated that stereotactic injection of ox-mtDNA into the mouse SNpc recapitulates PD pathology. Utilizing magnetic-activated cell sorting (MACS) to isolate midbrain neurons and microglia for transcriptome sequencing, we showed that genes associated with PD exhibited significant alterations in midbrain neurons when comparing oxidized mtDNA group to the control group. Furthermore, genes related to the NLRP3 signaling pathway displayed substantial changes in midbrain microglia. Utilizing in vivo and in vitro models established with lipopolysaccharide (LPS) priming and rotenone treatment, we showed that the release of microglial ox-mtDNA drives NLRP3-dependent caspase-1 cleavage, IL-1β secretion, and neuronal death. We revealed a novel interaction between ox-mtDNA and NLRP3, in which ox-mtDNA directly binds to the intrinsically disordered region (IDR) of NLRP3, promoting NLRP3 activation. Our findings demonstrate a key role of mtDNA in driving the activation of the NLRP3-IL-1β axis in microglia, providing a mechanistic framework for targeting ox-mtDNA-driven microglial toxicity in PD.

## Results

### Ox-mtDNA induces neuronal damage in vivo

**Fig. 1:**
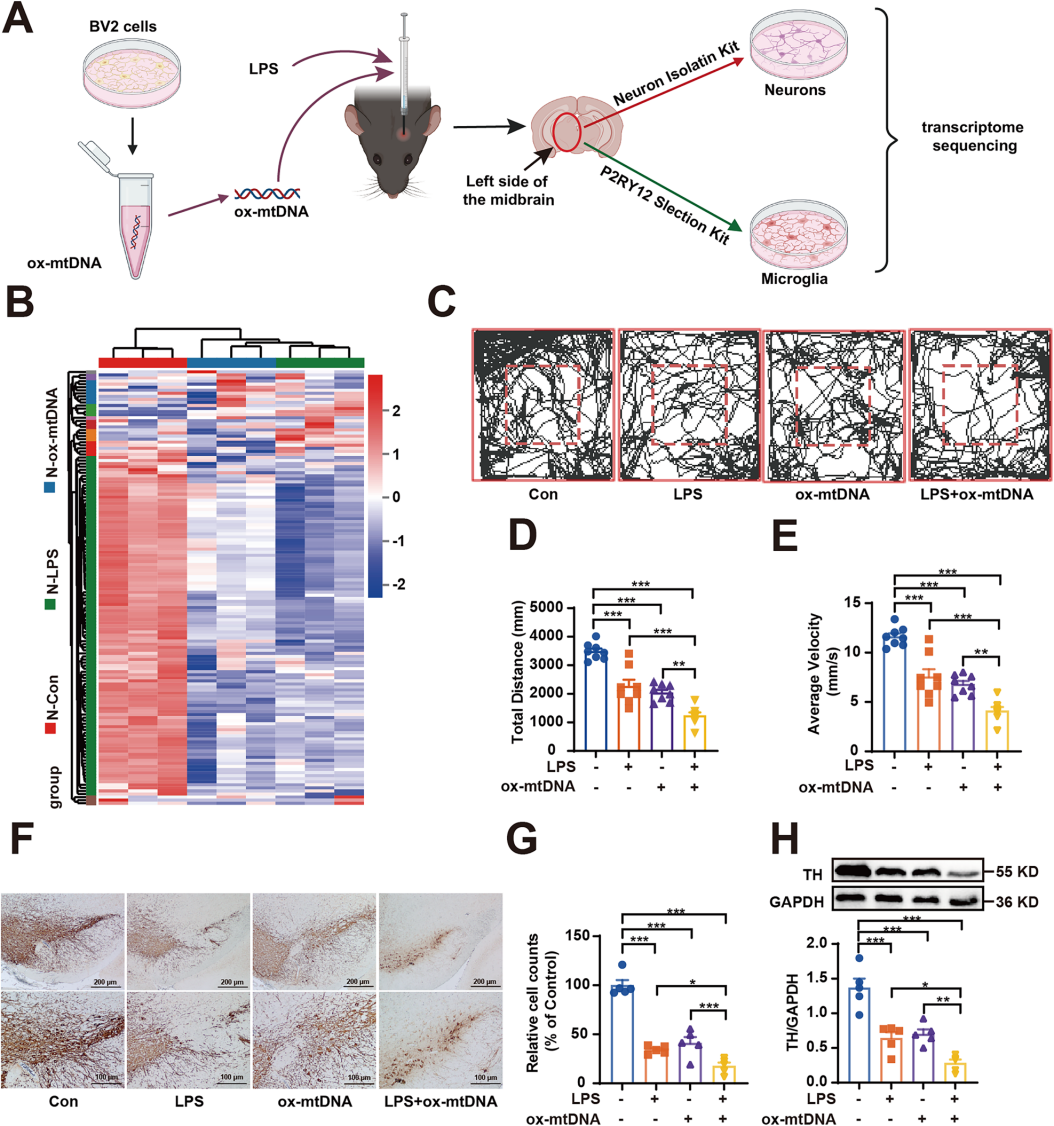
Ox-mtDNA induced pathogenesis of PD*.* **A** LPS (4 µg/2 µL) and ox-mtDNA (2 µg/2 µL) induced PD and neurons and microglia were sorted by magnetic beads for transcriptome sequencing separately. **B** Heatmap of Parkinson’s disease-related genes. Red indicates the upregulation, and blue indicates the downregulation in heatmaps (*n* = 3). **C** Representative traces of total distance traveled, **D** the total distance travelled and **E** the average velocity in the open field test (*n* = 8). **F**, **G** Immunohistochemical staining and quantification of TH positive neurons in SN were determined (*n* = 5). Scale bar = 200 μm (top), 100 μm (bottom). **H** TH protein level in the midbrain was examined by western blotting (*n* = 5). Data are presented as mean ± SEM. *p < 0.05, **p < 0.01, and ***p < 0.001.

Growing evidence has highlighted the emerging role of ox-mtDNA as a critical DAMP in neuroinflammation [23, 24]. Recent studies have increasingly focused on the involvement of ox-mtDNA in PD [9]; however, the precise mechanisms linking ox-mtDNA-mediated microglial responses to neuronal damage remain poorly understood. To address this knowledge gap, we first generated and extracted ox-mtDNA in vitro by treating BV2 cells with LPS and rotenone. The oxidized state was confirmed by a significant increase in the levels of 8-OHdG, as assessed through dot-blot analysis (Fig. S1A). Subsequently, we established a PD model by stereotactically injecting the validated ox-mtDNA into the SNpc of C57BL/6 mice (Fig. 1A). We subsequently employed MACS to isolate midbrain neurons and microglia from mice, followed by RNA sequencing to profile the transcriptional alterations in neurons and microglia induced by ox-mtDNA exposure (Fig. 1A). The purity of the isolated cell populations was confirmed through immunostaining for the neuronal marker MAP2 and the microglial marker Iba-1 (Fig. S1B, C).

The principal component analysis demonstrated a clear separation of neurons and microglia along the PC1 axis, with no overlap between the two cell types (Fig. S1D), indicating a significant distinction between neurons and microglia. Additionally, the intersection of the Venn diagrams illustrated the heterogeneity of isolated neurons and microglia (Fig. S1E). Subsequently, transcriptomic changes were compared between the LPS and control groups, and between the ox-mtDNA and control groups in isolated neurons from mice, leading to a comprehensive analysis of differentially expressed genes (DEGs) among the groups. A total of 1276 upregulated DEGs and 1628 down-regulated DEGs were identified in the ox-mtDNA group compared to the control group (Fig. S1F). In comparing the LPS and control groups, 3237 DEGs were detected, with 1502 DEGs upregulated and 1735 DEGs down-regulated (Fig. S1G). Hierarchical clustering analysis of 142 PD-related genes in neurons revealed that genes associated with the improvement of PD, such as LRRK2, were significantly down-regulated, while genes that promote PD, such as α-synuclein, were significantly upregulated in the LPS and ox-mtDNA groups (Fig. 1B). These findings suggest that ox-mtDNA can influence the progression of PD in mice.

We further investigated the behavior changes and DA neuronal damage in ox-mtDNA-induced mice. As illustrated in Fig. 1C–E, LPS and ox-mtDNA synergistically exacerbated neurological dysfunction, as evidenced by a significant reduction in both total distance traveled and average velocity during the open-field test. Immunohistochemical staining revealed a marked increase in the loss of tyrosine hydroxylase (TH)-positive DA neurons in mice injected with ox-mtDNA, which was further exacerbated by LPS priming (Fig. 1F, G). Similar findings were observed in TH protein expression analysis (Fig. 1H). These results indicate that ox-mtDNA can cause DA neuronal damage.

### Ox-mtDNA activates microglial NLRP3-IL-1β axis in vivo

**Fig. 2:**
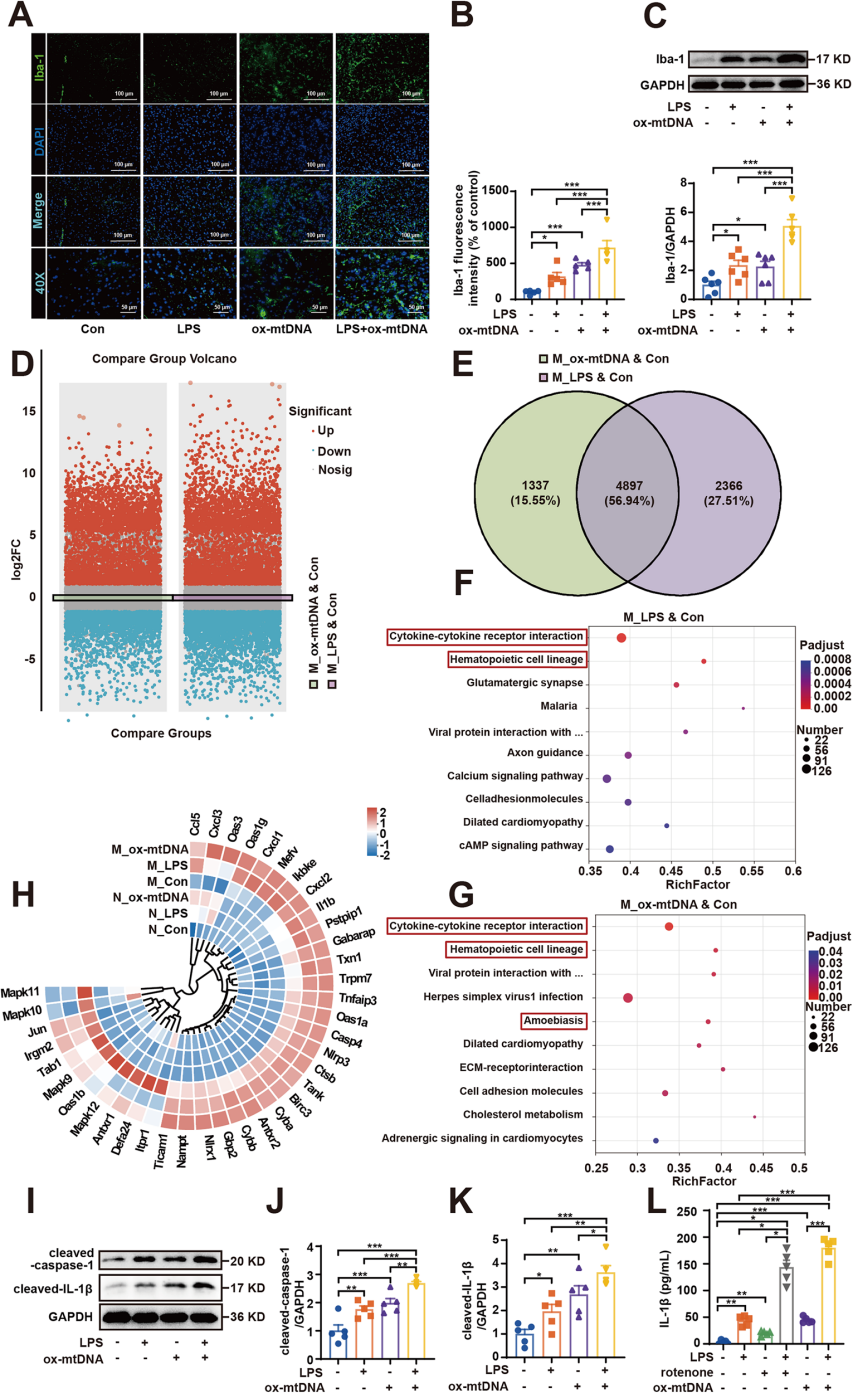
Ox-mtDNA activates microglial NLRP3-IL-1β axis in vivo. **A**, **B** Immunofluorescence staining and quantification of the fluorescence intensity of Iba-1 (green) in the midbrain were determined (*n* = 5). Scale bar = 100 μm (top), 50 μm (bottom). **C** Iba-1 protein level in the midbrain was examined by western blotting (*n* = 5). **D** The comparison of the number and Volcano plot of DEGs between mouse microglial LPS & Con groups and ox-mtDNA & Con groups (*n* = 3). **E** Venn diagram represented the DEPs of mouse microglial LPS & Con groups and ox-mtDNA & Con groups (*n* = 5). **F** The top ten KEGG pathways enriched by DEGs in the mouse microglial LPS & Con groups and **G** the mouse microglial ox-mtDNA & Con groups (*n* = 5). **H** Heatmap of target gene set circles of NLRP3 signaling pathway related genes. Red indicates the upregulation, and blue indicates the downregulation in heatmaps. **I**–**K** cleaved-caspase-1 and cleaved-IL-1β protein expressions in LPS (4 µg/2 µL) primed ox-mtDNA (2 µg/2 µL) induced mice midbrain were measured by western blotting (*n* = 5). **L** Serum IL-1β levels were measured in mice primed with LPS (4 µg/2 µL) and induced with ox-mtDNA (2 µg/2 µL) or rotenone (1.5 mg/kg) using ELISA kits. (*n* = 5). Data are presented as mean ± SEM. *p < 0.05, **p < 0.01, and ***p < 0.001.

Further analysis demonstrated that ox-mtDNA triggered significant activation of midbrain microglia, synergistically enhancing the activation in LPS-primed microglia (Fig. 2A–C). Analysis of DEGs from transcriptional sequencing of midbrain microglia revealed 4282 DEGs upregulated and 2981 DEGs downregulated in the LPS group compared to the control group. In the ox-mtDNA group, 4060 DEGs were upregulated and 2174 DEGs were down-regulated relative to the control group (Fig. 2D). Notably, 56.94% of these DEGs were co-regulated between the two comparison groups (Fig. 2E). Subsequent analysis of Kyoto Encyclopedia of Genes and Genomes (KEGG) pathways enriched for DEGs identified that inflammation-related signaling pathways were significantly enriched. Particularly, IL-1β is strongly associated with these inflammatory pathways (Fig. 2F, G). NLRP3 is a key mediator of IL-1β production during microglial activation [25]. Gene expression analysis further identified 38 DEGs involved in the NLRP3 inflammasome pathway (KEGG: mmu04621) co-regulated by LPS and ox-mtDNA in both microglia and neurons. Notably, NLRP3 and IL-1β were significantly upregulated, with microglia exhibiting markedly higher expression levels than neurons (Fig. 2H). NLRP3 inflammasome activation is a multi-step process involving the oligomerization of the adapter protein ASC and the self-cleavage of caspase-1, which then processes pro-IL-1β into its active form [20]. Consistent with this mechanism, our analysis of midbrain tissues revealed that ox-mtDNA treatment increased the protein levels of ASC and pro-IL-1β (Fig. S2A, B, D), indicative of transcriptional priming. Crucially, we observed a concomitant rise in the cleaved forms of both caspase-1 and IL-1β (Fig. 2I–K), while the level of pro-caspase-1 remained unchanged (Fig. S2A, C). Furthermore, ox-mtDNA led to a significant increase in serum IL-1β concentrations (Fig. 2L). These results demonstrate that ox-mtDNA induces robust NLRP3 inflammasome assembly and activation, underscoring its crucial role in triggering microglial activation and subsequent neuronal damage.

### Ox-mtDNA-NLRP3-IL-1β axis is involved in LPS-primed rotenone-induced neuronal damage in vivo

**Fig. 3:**
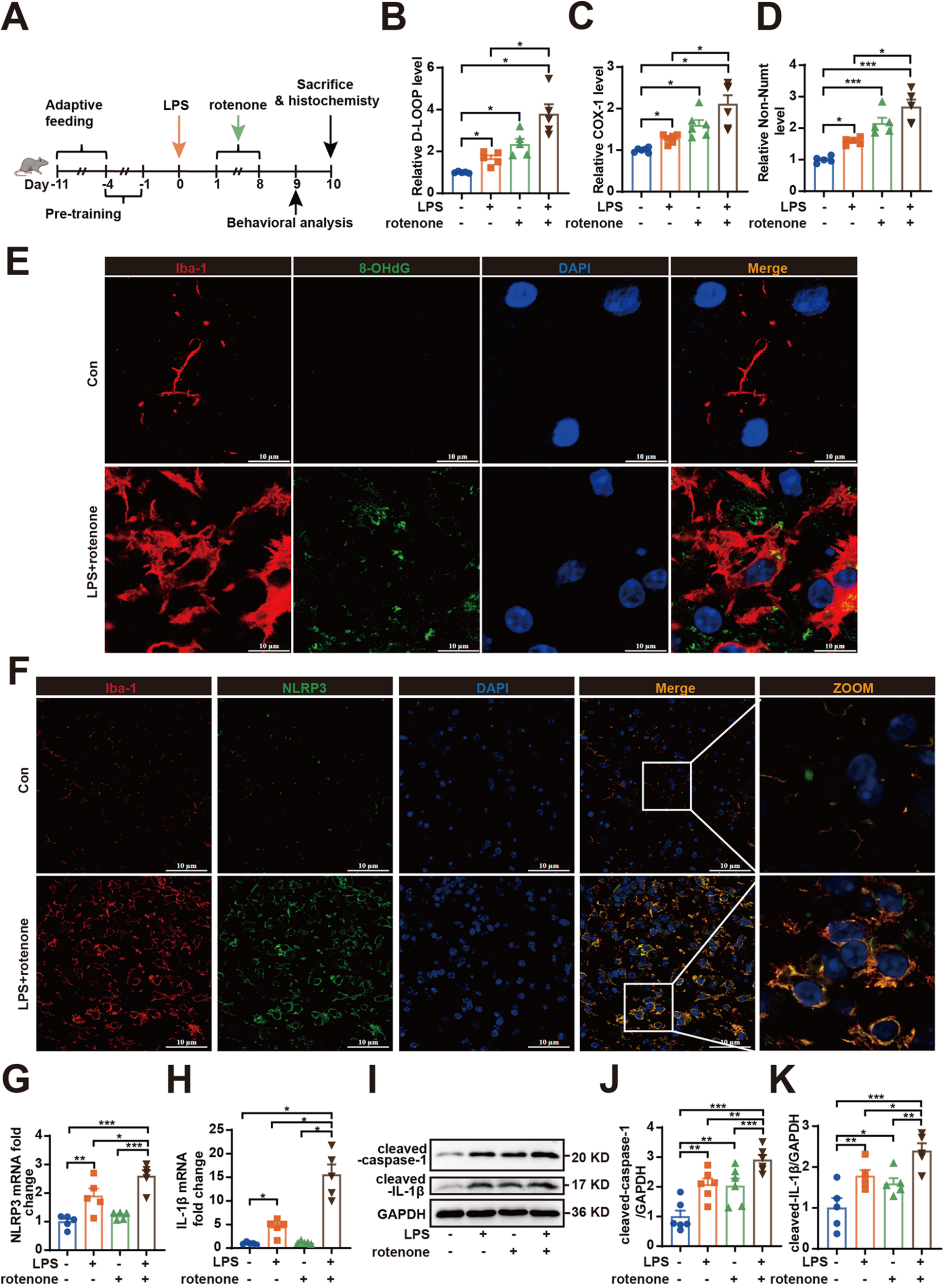
Ox-mtDNA-NLRP3-IL-1β axis is involved in LPS-primed rotenone-induced neuronal damage in vivo. **A** Schematic representation of the experimental design for PD induction in mice. **B**–**D** Relative amounts of total mtDNA in cytosols of midbrains from mice primed with LPS (4 µg/2 µL) and stimulated with rotenone (1.5 mg/kg). **E** Co-localization of 8-OHdG (green) with Iba-1 (red) assessed via confocal microscopy. Scale bar = 10 μm. **F** Co-localization of NLRP3 (green) with Iba-1 (red) assessed via confocal microscopy. Scale bar = 10 μm. **G**, **H** NLRP3 and IL-1β mRNA expression level in midbrain detected by RT-qPCR (*n* = 5). **I**–**K** cleaved-caspase-1 and cleaved-IL-1β protein expressions in LPS (4 µg/2 µL) primed rotenone (1.5 mg/kg) induced mice midbrain were measured by western blotting (*n* = 5). Data are presented as mean ± SEM. *p < 0.05, **p < 0.01, and ***p < 0.001.

Rotenone induces a burst of ROS by specifically inhibiting mitochondrial CI [26]. This oxidative stress can promote mtDNA oxidation and release, forming a positive feedback loop of ox-mtDNA [27]. Based on the findings of ox-mtDNA-induced neuronal injury and upregulation of NLRP3 and IL-1β in microglia revealed in the above experiments, we mimicked the pathological environment of PD using LPS and rotenone to further investigate the mechanism and role of ox-mtDNA-mediated NLRP3-IL-1β axis activation in PD pathology (Fig. 3A).

Under this PD model, we assessed microglial activation. As shown in Fig. S3A, B, through Iba-1 immunofluorescence staining analysis, the combination of LPS and rotenone induced further microglial activation compared to either treatment alone. Correspondingly, Iba-1 protein expression analysis yielded similar results (Fig. S3C). Additionally, we observed an increase in cytoplasmic mtDNA in the midbrain upon exposure to LPS and rotenone. And the combination of LPS and rotenone resulted in a greater release of mtDNA compared to either the LPS or rotenone exposure alone (Fig. 3B–D). To further determine whether the mtDNA released upon LPS + rotenone exposure was oxidized and localized within microglia, we performed confocal microscopy analysis. The results clearly showed that the cytoplasmic DNA was significantly oxidized and co-localized with activated microglia (Fig. 3E). These results suggest that LPS combined with rotenone induces microglial activation, leading to the oxidation and release of their mtDNA.

As illustrated in Fig. S3D–F, LPS-primed rotenone-induced PD mice exhibited significant exacerbation in the open field test compared to the groups exposed to LPS alone or rotenone alone, evidenced by a reduction in both total distance travel and average velocity in the open field test. These findings suggest that rotenone exacerbated neurological deficits in LPS-primed PD mice. Consistently, we observed an increase in the loss of TH-positive DA neurons in LPS + rotenone mice compared to those exposed to LPS alone or rotenone alone (Fig. S3G, H). Similar results were obtained from TH protein expression analysis (Fig. S3I). These results indicate that rotenone and LPS synergistically exacerbated DA neuronal damage in mice.

Based on the transcriptome sequencing results that ox-mtDNA induced neuronal damage and upregulated the genes associated with the microglial NLRP3-IL-1β axis (Figs. 1 and 2), we propose that LPS and rotenone triggers the NLRP3-IL-1β axis in microglia by releasing ox-mtDNA, which subsequently leads to neuronal damage. To further investigate this hypothesis under the condition of maximal mtDNA release, we employed laser confocal microscopy to observe the expression of NLRP3 in midbrain microglia. The results indicated that NLRP3 expression was significantly upregulated in microglia following treatment with LPS and rotenone (Fig. 3F). Then the mRNA levels of NLRP3 and IL-1β in midbrain tissues, as well as the downstream proteins cleaved-caspase-1 and cleaved-IL-1β were analyzed. The results demonstrated that rotenone significantly increased the mRNA levels of NLRP3 and IL-1β when primed with LPS but not with rotenone alone (Fig. 3G, H). Notably, treatment with rotenone resulted in the upregulation of ASC, pro-IL-1β (Fig. S3J, K, M), cleaved-caspase-1, and cleaved-IL-1β (Fig. 3I–K), as well as an increase in serum IL-1β levels (Fig. 2L), while the level of pro-caspase-1 unchanged (Fig. S3J, L). These effects were synergistically enhanced following LPS priming. However, the combination of LPS and rotenone did not alter the protein level of pro-caspase-1. Collectively, these results suggest that promoting microglia ox-mtDNA release and thus activating the NLRP3-IL-1β axis may be an essential mechanism by which rotenone exacerbates PD in the LPS-primed PD model.

### Ox-mtDNA is released in LPS-primed rotenone-induced BV2 cells

**Fig. 4:**
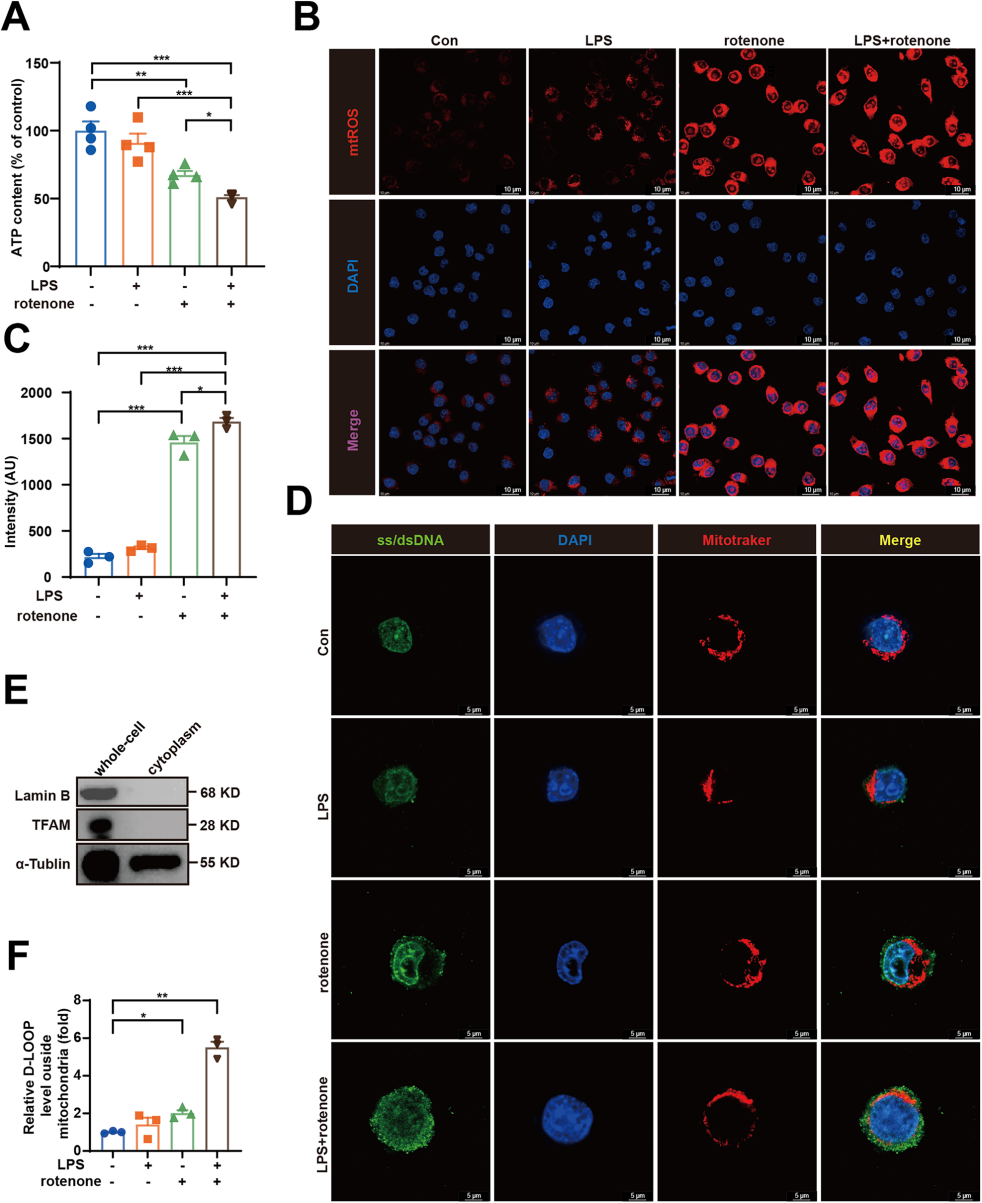
Ox-mtDNA is released in LPS-primed rotenone-induced BV2 cells. **A** ATP content in BV2 cells (*n* = 3). **B**, **C** mtROS (red) production detected by MitoSOX Red staining and Quantitation of mtROS (*n* = 3). Scale bar = 25 μm. **D** The extra-mitochondrial ss/dsDNA (green) in LPS (100 ng/mL) primed rotenone (0.1 μM) treated BV2 cells was detected by Laser confocal microscopy. Scale bar = 5 μm**. E** BV2 cells cytosolic DNA was isolated and purified using the method in the Methods and whole-cell and cytosolic proteins were blotted using the Lamin B, TFAM and α-Tublin antibodies. **F** Relative amounts of total mtDNA in cytosols of LPS (100 ng/mL) primed rotenone (0.1 μM) stimulated BV2 cells (*n* = 3). Data are presented as mean ± SEM. *p < 0.05, **p < 0.01, and ***p < 0.001.

A prominent mechanism associated with ox-mtDNA escape involves ROS-induced oxidative stress [28]. Studies have demonstrated that the mitochondrial CI inhibitor rotenone increased ROS production and reduced ATP levels [26, 29]. Therefore, we investigated the mitochondrial function and related mtDNA release in BV2 cells challenged with LPS and rotenone and found that rotenone, but not LPS, significantly decreased ATP levels (Fig. 4A) and increased mitochondrial ROS production, as measured by the MitoSOX fluorescence probe (Fig. 4B, C). To investigate rotenone-induced mtDNA release, we quantified cytoplasmic mtDNA levels and performed confocal microscopy. Both assays demonstrated that rotenone exposure significantly increased mtDNA leakage, with LPS further enhancing this effect (Fig. 4D–F). mtDNA is particularly vulnerable to oxidation by ROS. Due to its high redox potential, guanine is the most prone to oxidation, resulting in the formation of 8-hydroxy-2-deoxyguanosine (8-OHdG) [30], a well-established marker of ROS-induced DNA lesions [31]. Dot blotting analysis utilizing an antibody against 8-OHdG confirmed that LPS and rotenone induced oxidative damage to mtDNA (Fig. S1A). These findings indicate that LPS and rotenone cooperatively induce mitochondrial dysfunction, promoting ox-mtDNA cytoplasmic release.

### NLRP3-IL-1β axis is involved in LPS-primed rotenone-induced neuronal damage

**Fig. 5:**
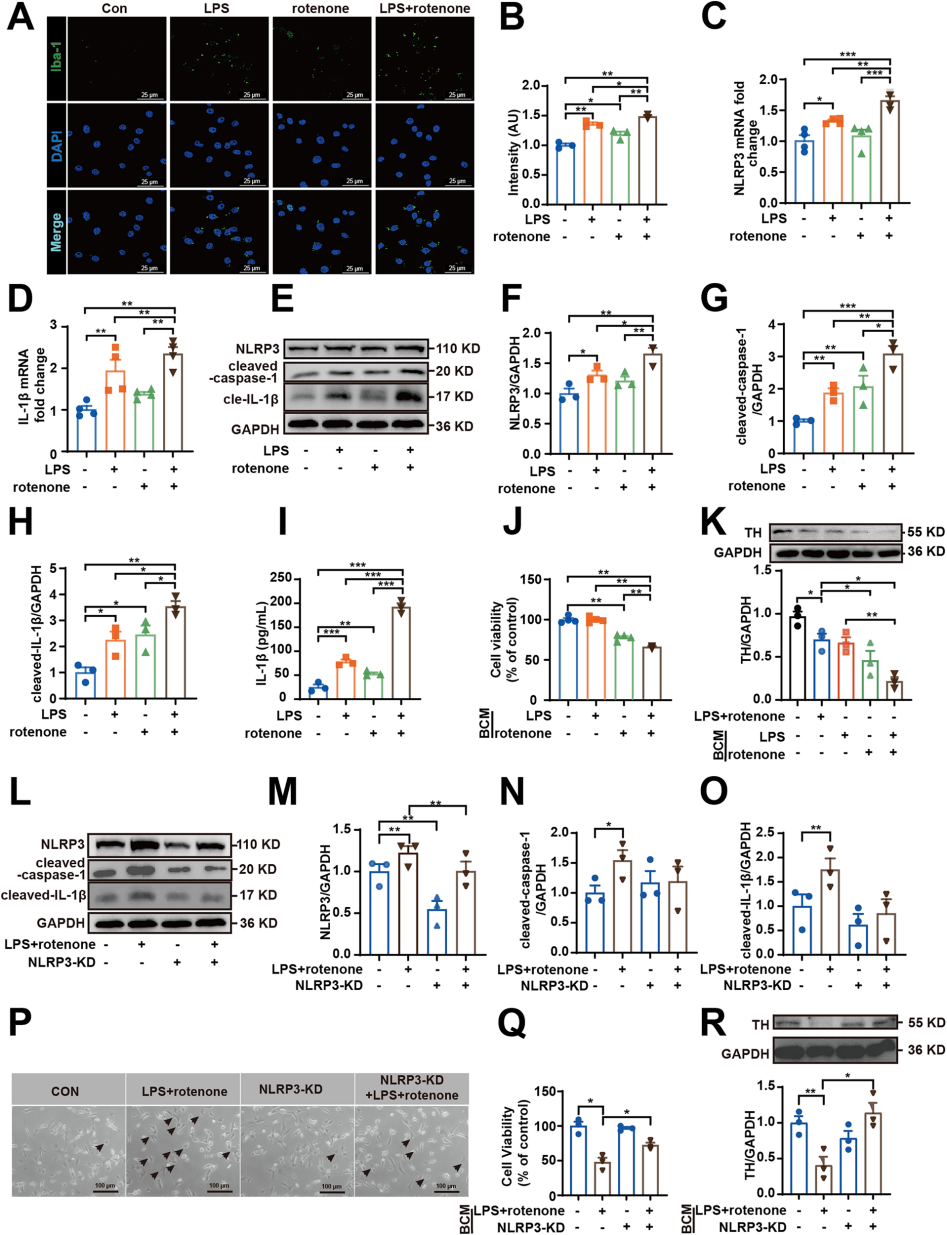
NLRP3-IL-1β axis is involved in LPS-primed rotenone-induced neuronal damage. **A**, **B** Immunofluorescence staining and quantification of the fluorescence intensity of Iba-1 (green) in BV2 cells were determined (*n* *=* 3). Scale bar = 25 μm. **C**, **D** NLRP3 and IL-1β mRNA expression level in BV2 cells detected by RT-qPCR (*n* *=* 3). **E**–**H** NLRP3, cleaved-caspase-1 and cleaved-IL-1β protein expressions in LPS (100 ng/mL) primed rotenone (0.1 μM) induced BV2 cells were measured by western blotting (*n* *=* 3). **I** IL-1β levels in medium were quantified using ELISA kits (*n* *=* 3). **J** SH-SY5Y cells were incubated with the BCM from BV2 cells for 24 h and cell viability was measured with MTT assay and **K** TH protein expression was measured by western blotting (*n* *=* 3). **L**–**O** NLRP3, cleaved-caspase-1 and cleaved-IL-1β protein expressions in NLRP3-KD BV2 cells treated with LPS (100 ng/mL) and rotenone (0.1 μM) were measured by western blotting (*n* *=* 3). **P** SH-SY5Y cells were incubated with the BCM from NLRP3-KD BV2 cells for 24 h, and microscopic observation of morphological changes (arrowed) in SH-SY5Y cells, Scale bar = 100 μm, **Q** cell viability was measured with MTT assay, and **R** TH protein expression was measured by western blotting (*n* *=* 3). Data are presented as mean ± SEM *p *<* 0.05, **p *<* 0.01, and ***p *<* 0.001.

BV2 cells were utilized to further explore the mechanism of neuronal damage mediated by microglia activation (Fig. 5A, B). Consistent with the results of the animal experiments, similar findings were observed in the mRNA levels of NLRP3 and IL-1β (Fig. 5C, D), as well as in the protein expression of ASC, pro-caspase-1, pro-IL-1β (Fig. S4A–D, NLRP3, cleaved-caspase-1 and cleaved-IL-1β (Fig. 5E–H) and the levels of IL-1β in the culture medium (Fig. 5I). The BV2 conditioned medium (BCM) was collected and co-cultured with SH-SY5Y cells. BCM collected from LPS-treated cells did not decrease the viability of SH-SY5Y cells; however, it significantly exacerbated the damage of SH-SY5Y cells induced by rotenone-treated BCM (Fig. 5J) and led to further downregulation of TH protein expression (Fig. 5K). These in vitro findings establish microglial NLRP3 as a key mediator linking LPS and rotenone exposure to neuronal injury.

To investigate NLRP3’s role in neuronal damage, we generated NLRP3-knockdown (NLRP3-KD) BV2 cells using lentiviral shRNA. The staining efficiency of lentivirus targeting NLRP3 was demonstrated by immunofluorescence in Fig. S4E, and verified at the protein level with 54.9% relative to the scramble group (Fig. S4F). LPS + rotenone treatment significantly upregulated NLRP3, cleaved-caspase-1 and cleaved-IL-1β protein expression in wild-type cells, but these effects were markedly attenuated in NLRP3-KD cells (Fig. 5L–O). Given that microglia-mediated neuroinflammation ultimately leads to neuronal damage, we specifically examined whether NLRP3 inflammasome activation in microglia drives this neurotoxicity. As shown in Fig. 5P–R, BCM (LPS + rotenone + NLRP3-KD) significantly attenuated neuronal toxicity compared to BCM (LPS + rotenone), as demonstrated by improved cell viability and restored TH expression. To further validate the specificity of NLRP3 in mediating neuroinflammation, we extended our investigation to an in vivo model using the NLRP3 inhibitor MCC950. In the LPS + rotenone group, immunohistochemical analysis revealed a significant reduction in TH expression in the SNpc, indicative of DA neuron loss, which was robustly reversed by MCC950 co-treatment (Fig. S5A, B). Conversely, Iba-1 immunofluorescence demonstrated pronounced microglial activation in LPS + rotenone mice, while MCC950 attenuated this effect (Fig. S5C, D). Western blotting corroborated these findings, showing that LPS + rotenone mice had decreased TH levels and increased Iba-1 protein levels, which were reversed by MCC950. Notably, NLRP3 inflammasome component, ASC, was upregulated in LPS + rotenone mice, however, MCC950 treatment suppressed its expression. While the expression of the precursor pro-caspase-1 remained unchanged across groups, the levels of its active form, cleaved-caspase-1, along with the mature cytokine cleaved-IL-1β and its precursor pro-IL-1β, were significantly elevated. MCC950 treatment effectively suppressed all these activation markers without altering pro-caspase-1 levels (Fig. S5E–L). These results establish that microglial NLRP3-IL-1β axis activation is essential for LPS + rotenone-induced neurotoxicity. Parallel results were observed in the ox-mtDNA-injected model. MtDNA alone reduced TH expression and enhanced Iba-1 activation, whereas MCC950 rescued both effects (Fig. S5A, B). Inflammasome protein profiles mirrored those in LPS + rotenone mice, with MCC950 suppressing ASC, cleaved-caspase-1, cleaved-IL-1β, and pro-IL-1β but not pro-caspase-1 (Fig. S5M–T). Collectively, these in vivo pharmacologic inhibition data, combined with in vitro genetic knockdown approaches, unequivocally demonstrate that NLRP3 is the crucial mediator of microglia-dependent neurotoxicity in PD models.

### Ox-mtDNA activates NLRP3 by binding to NLRP3 in LPS-primed rotenone-induced BV2 cells

**Fig. 6:**
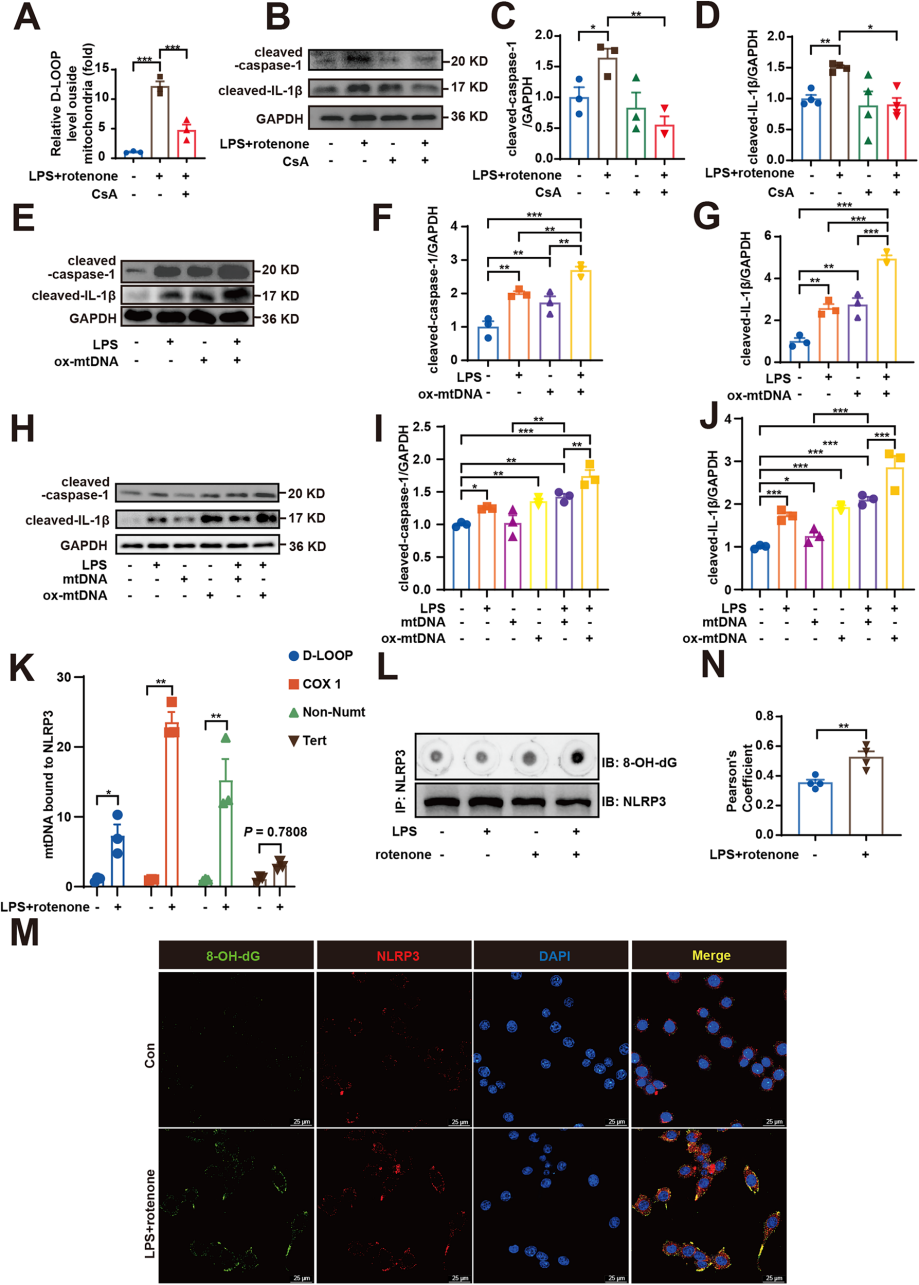
Ox-mtDNA activates NLRP3 via binding to it in LPS-primed rotenone-induced BV2 cells. **A** BV2 cells were pretreated with CsA (2 μM) followed by LPS (100 ng/mL) + rotenone (0.1 μM) stimulation, and the relative cytoplasmic mtDNA content of BV2 cells was detected, and **B**–**D** cleaved-caspase-1 and cleaved-IL-1β protein expressions were measured by western blotting (*n* *=* 3). **E**–**G** cleaved-caspase-1 and cleaved-IL-1β protein expressions in BV2 cells transfected with mtDNA derived from BV2 cells were measured by western blotting (*n* *=* 3). **H**–**J** BV2 cells were transfected with synthetic mtDNA (with or without hydrogen peroxide oxidation) for 24 h, and cleaved-caspase-1 and cleaved-IL-1β protein expression were examined (*n* *=* 3). **K** The protein lysate from BV2 cells after indicated treatments were immunoprecipitated with NLRP3 antibodies. DNA were eluted and purified from the immunocomplexes, and the relative amounts of mtDNA (D-LOOP, COX-1, Non-Numt) and nuclear DNA were measured by RT-qPCR, **L** and the immunocomplexes were spotted on a nitrocellulose membrane, UV-crosslinked and probed with antibodies to 8-OHdG. **M** Representative images and **N** colocalization analysis of 8-OHdG (green) colocalizing with NLRP3 (red) in BV2 cells upon LPS + rotenone (*n* *=* 3). Scale bar = 25 μm. Data are presented as mean ± SEM. *p *<* 0.05, **p *<* 0.01, and ***p *<* 0.001.

Previous studies have suggested that ox-mtDNA indirectly regulates NLRP3 activity, for instance, through TLR9 [32]. Here, we further define the interaction of ox-mtDNA with NLRP3. We employed both positive and negative approaches to determine the effect of mtDNA on NLRP3 activation: (1) intervention with the mtDNA release inhibitor cyclosporine A (CsA) and (2) transfection of ox-mtDNA derived from BV2 cells treated with LPS and rotenone. As illustrated in Fig. 6A, CsA significantly reduced ox-mtDNA release induced by LPS and rotenone. Meanwhile, CsA notably downregulated the protein expression of cleaved-caspase-1 and cleaved-IL-1β (Fig. 6B–D). These findings suggest that the inhibition of mPTP opening, along with the subsequent reduction in the release of mitochondrial DAMPs, including mtDNA, contributes to the suppression of NLRP3 activation. In contrast, the expression levels of these proteins were markedly upregulated following transfection with ox-mtDNA generated and extracted from BV2 cells treated with LPS and rotenone (Fig. 6E–G), indicating that the release of ox-mtDNA plays a role in the activation of NLRP3. Subsequently, we investigated whether there are differences in NLRP3 activation between oxidized and non-oxidized mtDNA. D-loop mtDNA was synthesized and then oxidized with hydrogen peroxide (Fig. S6A), and agarose gel electrophoresis showed that oxidation of mtDNA by hydrogen peroxide did not result in mtDNA breaks (Fig. S6B). The results showed that compared with normal mtDNA, ox-mtDNA significantly enhanced the protein expression of cleaved-caspase-1 and cleaved-IL-1β (Fig. 6H–J). Taken together, these data indicate that ox-mtDNA can induce preferential activation of NLRP3 compared with normal mtDNA.

We further hypothesized that ox-mtDNA might bind to NLRP3. Therefore, we immunoprecipitated (IP) NLRP3 with anti-NLRP3 antibody, and IP products were purified, and the relative amount of mtDNA was detected by RT-qPCR. mtDNA but not nuclear DNA was significantly detected in the NLRP3 IP product of BV2 cells after LPS + rotenone challenged (Fig. 6K). IP products were directly subjected to 8-OHdG dot blotting, and 8-OHdG signal was clearly detected (Fig. 6L). Confocal microscopy showed that after LPS + rotenone treatment, the co-localization of 8-OHdG and NLRP3 yielded similar results (Fig. 6M, N). These results indicate that ox-mtDNA is binding with NLRP3.

### Ox-mtDNA binds directly to the IDR of NLRP3 and activates NLRP3

**Fig. 7:**
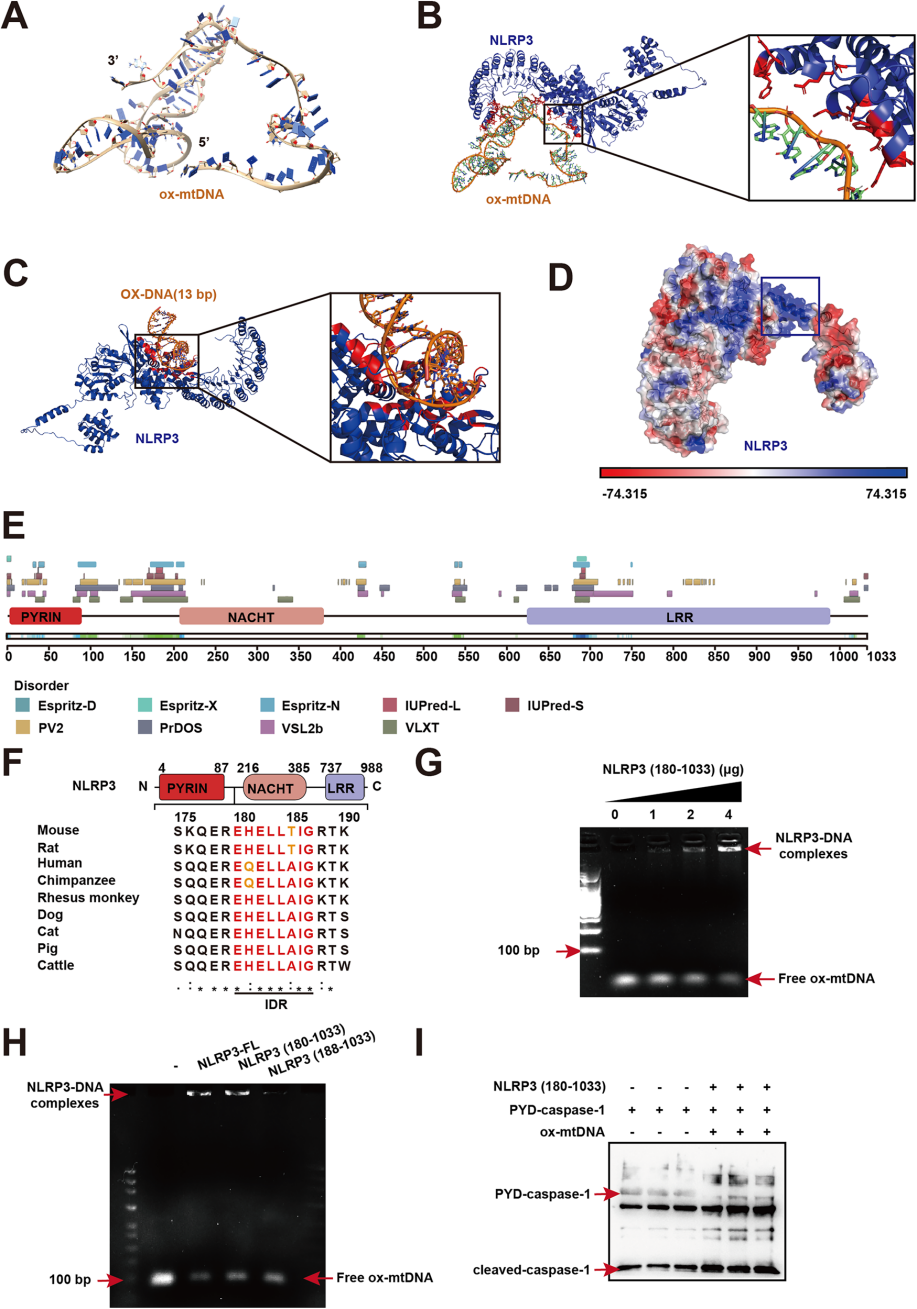
NLRP3 activation by ox-mtDNA through its direct binding to the IDR of NLRP3. **A** The 3D conformation of mtDNA (D-LOOP 90 bp) was predicted using the 3dDNA online program (http://biophy.hust.edu.cn/new/3dRNA), and the hydrogen atom at position C8 on the guanine base in the mtDNA was replaced with a hydroxyl group using the Chimera software and structure optimisation was performed to obtain the 3D conformation of ox-mtDNA. **B** The ox-mtDNA bound to the 180-187 sequences of NLRP3 (PDB 7PZC). **C** The ox-DNA (PDB 3I0W) bound to the 180-187 sequences of NLRP3 (PDB 7PZC). **D** NLRP3 molecule from PDBID 7pzc. Electrostatic surface potential illustrates the putative DNA binding site (the box). **E** The sequence NLRP3 (180-187) and NLRP3 (690-695) are predicted to be disordered regions of NLRP3 by 75% comprehensive database. **F** Sequence comparisons using Clustal Omega showed that the IDR of NLRP3 (180-187) is highly conserved among various NLRP3 homologs. **G** SYBR stained agarose gel showing EMSA with the indicated amounts of purified His6-tagged NLRP3 (180-1033) and ox-mtDNA (2 μg). **H** SYBR stained agarose gel showing the EMSA with 2 μg of purified of NLRPR (FL), NLRP3 (188-1033) or NLRP3 (180-1033) (4th lane) and poly (dA:dT) (2 μg). **I** Immunoblot for cleaved-caspase-1 following in vitro incubation of PYD-caspase-1 with purified NLRP3 (180-1033).

To further characterize the interaction of ox-mtDNA with NLRP3, we predicted the 3D conformation of ox-mtDNA (Fig. 7A). The dominant binding conformation of ox-mtDNA to NLRP3 (PDB 7PZC) was further predicted using the Z-dock program. The results suggested that ox-mtDNA could bind with an unstructured domain of NLRP3 between the NACHT and PYD domains (△G = -10.9) (Fig. 7B). Consistently, molecular docking of NLRP3 (PDB 7PZC) with ox-DNA (PDB 3I0W) showed a similar result (△G = -14.6) (Fig. 7C). The NLRP3 molecule possesses a significantly large positive surface, which includes the region predicted to interact with ox-mtDNA as determined by molecular docking studies (Fig. 7D). Intrinsically disordered regions (IDR) are oftentimes known to convert from disordered to ordered, or vice versa, when interacting with macromolecules [33]. Given that the region predicted to bind directly to oxidized ox-mtDNA appears predominantly disordered in NLRP3, we investigated whether NLRP3 conforms to the canonical definition of an intrinsically disordered protein (IDP). Analysis using the D^2^P^2^ software revealed that two sequences, exhibiting over 75% consistency, fulfill the criteria for IDR as defined by the prediction program: residues 180-187 and 690-695 (Fig. 7E). Sequence comparisons using Clustal Omega revealed that the segment of the IDR, specifically residues 180-187, is highly conserved among NLRP3 homologs across various species (Fig. 7F). This significant degree of conservation suggests that these sequences may possess crucial biological functions [34]. Together, these results indicate that ox-mtDNA may bind directly to NLRP3.

To further investigate whether mtDNA directly binds to NLRP3, we conducted electrophoretic mobility shift assays (EMSA). Our results showed that the binding band strength between truncated NLRP3 (180-1033) and ox-mtDNA increased with the increase of truncated NLRP3 (180-1033) content, which indicates that ox-mtDNA can directly bind to truncated NLRP3 (180-1033) (Fig. 7G). When the 180-187 region of NLRP3 was truncated, the binding band intensity of truncated NLRP3 (188-1033) to ox-mtDNA was significantly weakened, suggesting that ox-mtDNA binds directly to the 180-187 region of NLRP3. To gain a deeper understanding of whether ox-mtDNA activates NLRP3 when ox-mtDNA directly binds to truncated NLRP3 (180-1033), we incubated the truncated NLRP3 (180-1033) with ox-mtDNA followed by co-incubation with PYD-casepase-1 (Fig. 7H). As shown in Fig. 7I, a significant increase in cleaved-caspase-1 was detected in the co-incubation group. These results indicate that ox-mtDNA can directly bind to the truncated NLRP3 (180-1033) and activate NLRP3. To further validate the critical role of the NLRP3 180-187 sequence in its activation and to compare the relative potency of oxidized versus unoxidized mtDNA, we performed an in vitro PYD-caspase-1 cleavage assays. In this assay, different NLRP3 protein constructs were incubated with either oxidized (ox-) or unoxidized mtDNA. The results demonstrated that while unoxidized mtDNA was capable of inducing NLRP3 activation and generating cleaved-caspase-1, ox-mtDNA exhibited a markedly stronger effect. Crucially, when the NLRP3 180-187 sequence was truncated, the ability of the resulting protein to activate caspase-1 was significantly diminished upon stimulation by either form of mtDNA (Fig. S7F).

Taken together, ox-mtDNA is released in microglia in an LPS-primed rotenone-induced mouse PD model, binding directly with the 180-187 sequence of NLRP3 and resulting in NLRP3 activation in microglia. The knockdown of NLRP3 in vitro and the using of NLRP3 inhibitor in vivo attenuated the damage of neurons, highlighting a mechanism by which microglial NLRP3 could contribute to the progression of PD associated with neuroinflammation and mitochondrial stress. Consequently, strategies designed to prevent the release of microglial ox-mtDNA and its binding to the 180-187 sequence of NLRP3 may offer effective protective measures against PD.

## Discussion

This study demonstrated that ox-mtDNA activates the NLRP3-IL-1β axis in microglia, both in vitro and in vivo, driving PD-like pathology. Our work builds upon foundational studies, such as that by Sarkar et al., which established that mitochondrial damage in microglia amplifies NLRP3 inflammasome signaling to exacerbate dopaminergic neurodegeneration [35]. However, the precise identity of the mitochondrial-derived signal that directly initiates NLRP3 assembly remains unclear. Our study addresses this critical knowledge gap by identifying ox-mtDNA as the key DAMP that translates mitochondrial stress into NLRP3 inflammasome activation in microglia. Furthermore, we elucidate a novel mechanism by demonstrating that ox-mtDNA directly binds to the IDR of the NLRP3 protein, thereby facilitating inflammasome assembly. These findings provide a mechanistic insight into the critical role of mtDNA in linking microglial activation to neuronal injury in the context PD pathology. We further employed LPS plus rotenone models to simulate the environmental toxins induced neuropathology. Our findings showed that LPS and rotenone caused PD pathology by promoting ox-mtDNA release in microglia and activating the NLRP3-IL-1β axis. These results suggest that microglial mtDNA release-induced activation of the NLRP3-IL-1β axis is a key mechanism in the pathogenesis of PD. Mechanistically, we found that ox-mtDNA promotes NLRP3 activation by directly binding to the IDR region of NLRP3.

The pathogenesis of PD involves complex interactions among genetic predisposition, mitochondrial dysfunction, α-synuclein aggregation, and neuroinflammation [36]. While emerging studies highlight microglia-mediated neuroinflammation as a critical driver of PD progression [2, 3]. However, the precise mechanisms linking microglial inflammation to PD pathology remain poorly understood, partly due to the cellular complexity of the brain and the absence of direct evidence isolating the contributions of microglia from those of other neural or immune cells. This study employed MACS to isolate microglia and neurons from the mouse midbrain, followed by transcriptomic profiling. Although MACS of microglia has been used to study microglial neuroinflammation, there are few reports for PD research [37]. Transcriptomic analysis revealed that while both LPS and ox-mtDNA administration induced NLRP3 pathway gene regulation (e.g., NLRP3, IL-1β) in neurons, the effect was markedly more pronounced in microglia, providing direct evidence that microglial activation drives neurodegeneration. Furthermore, confocal microscopy demonstrated that the combination of LPS and rotenone synergistically induced cytoplasmic accumulation of ox-DNA and expression of NLRP3 in microglia. In vitro, LPS and rotenone-treated BV2 cells exhibited enhanced activation of the NLRP3-IL-1β axis. SH-SY5Y cells significantly reduced cell viability and TH expression when co-cultured with BCM. Our study hypothesizes that LPS primes microglial neuroinflammation, while rotenone amplifies this neuroinflammation by inducing microglial mitochondrial dysfunction, leading to oxidative stress that exacerbates DA neuronal damage. Notably, LPS alone was sufficient to induce cleavage of caspase-1 and IL-1β in the BV2 model, which is consistent with previous reports [38, 39]. This response may involve non-canonical pathways such as necroptosis or caspase-8 activation [35]. Nevertheless, it is clear that rotenone significantly amplifies NLRP3 inflammasome activation beyond the effect of LPS alone. Rotenone has long been employed as a model for inducing PD due to its capacity to directly inflict neuronal damage [40]. The effects of rotenone in in vivo experiments are extensive and may directly compromise DA neurons, which is an unavoidable aspect of our investigation. Consequently, we cannot definitively assert from the in vivo experiments alone that LPS initiates neuroinflammation in microglial cells and that rotenone amplifies this neuroinflammation through oxidative stress, exacerbating of PD. Nonetheless, the combination of rotenone and LPS exacerbates the activation of the NLRP3-IL-1β axis in midbrain microglia. Furthermore, our hypothesis is further supported by in vitro experiments. Direct administration of LPS and rotenone stimulation in SH-SY5Y cells for 24 h significantly decreased TH protein expression compared to the control group. However, the BCM subjected to the same exposure concentrations of LPS and rotenone stimulation for 12 h resulted in a further decrease in TH protein expression compared to SH-SY5Y cells stimulated with direct administration of LPS and rotenone. These results suggested that while LPS and rotenone directly induce neuronal damage, they also exacerbate microglial activation, contributing to neuronal damage. Collectively, while the potential contributions of other cell types (e.g., astrocytes or neurons) to PD pathogenesis are not excluded, it is undeniable that we have conclusively demonstrated the role of microglia in promoting PD, which was achieved through the integration of in vivo sorting, imaging, and in vitro co-culture system, providing actionable insights for therapies targeting microglial activation in PD.

Although the exact role of mitochondrial dysfunction in the pathogenesis of PD has not been fully defined, it is evident that mitochondrial damage may result in a range of adverse effects in cells, including bioenergetic collapse, interruption of signal transduction, and release of pro-inflammatory DAMP such as mtDNA. Mounting evidence implicates mtDNA as a key instigator of microglial neuroinflammation in PD [41]. In this study, we performed stereotactic injection of mtDNA extracted from LPS and rotenone-treated BV2 cells into the mouse midbrain, which triggered activation of midbrain microglia, accompanied by PD-related alterations, including dyskinesia and the loss of DA neurons. Transcriptomic profiling of sorted midbrain microglia and neurons further delineated the neuroinflammatory signatures induced by mtDNA specifically to microglia, thereby underscoring the central role of mtDNA in microglial function during the progression of PD. We note that the direct administration of ox-mtDNA in our models serves as a proof-of-concept to establish its intrinsic sufficiency as a DAMP. While this method bypasses endogenous release mechanisms, it provides direct causal evidence for ox-mtDNA’s neuroinflammatory capacity. This foundational finding is physiologically contextualized by our complementary data showing that pathophysiological stimuli (LPS + rotenone) trigger the endogenous oxidation and cytosolic release of mtDNA from microglia (Figs. 3 and 4), thereby bridging an exogenous challenge to a relevant cellular event.

Numerous alterations in inflammation-related factors, including TLR9, IL-10, and IL-12α, were identified among several thousand DEGs sequenced from the midbrain microglia transcriptome. While we do not exclude the activation of other inflammatory pathways in microglia, it is evident that IL-1β-related signaling pathways were significantly enriched among the top 10 signaling pathways identified through KEGG functional enrichment analysis. Furthermore, genes associated with the NLRP3 signaling pathway, such as NLRP3 and IL-1β, were markedly upregulated, indicating that the NLRP3-IL-1β axis plays a crucial role in the activation of microglia induced by mtDNA. These findings align with prior reports linking mtDNA to NLRP3 inflammasome priming [16], which was further supported by: (1) Functional analyses demonstrated that mtDNA upregulated NLRP3-associated genes in microglia, supported by both in vivo and in vitro evidence of NLRP3-IL-1β axis activation through immunofluorescence, immunoblot, RT-qPCR, and ELISA techniques. (2) Both the inhibition of mtDNA release and NLRP3 knockdown in BV2 cells significantly attenuated the production of cleaved caspase-1 and IL-1β, while the NLRP3 inhibitor MCC950 effectively suppressed inflammasome signaling induced by either LPS + rotenone or mtDNA. (3) Additionally, the neuroprotective role of targeting NLRP3 was demonstrated both in vitro, where its knockdown restored viability and TH expression in co-cultured dopaminergic neurons, and in vivo, where MCC950 attenuated LPS + rotenone- or mtDNA-induced neuronal damage and microglial activation. Notably, although our in vivo and in vitro data showed no significant change in pro-caspase-1 protein levels, this likely reflects its dynamic processing. Inflammatory activation may lead to increased synthesis of pro-caspase-1, but concurrent cleavage into its active form may mask detectable changes in total pro-caspase-1 expression when measured by Western blot. This observation aligns with previous studies, where robust cleavage and elevated levels of cleaved-caspase-1 were observed despite stable pro-caspase-1 levels [42, 43]. Importantly, our study specifically implicates ox-mtDNA as a direct NLRP3 activator in microglia—a mechanism previously unexplored in PD models. Notably, ox-mtDNA demonstrated superior potency in activating NLRP3 [14, 15]. Dot-blot assays confirmed elevated levels of 8-OHdG in mtDNA from LPS + rotenone-treated BV2 cells, which may be attributed to the overproduction of mitochondrial ROS. Synthetic ox-mtDNA, generated via H_2_O_2_ treatment, robustly induced cleaved-caspase-1 and cleaved-IL-1β in BV2 cells compared to non-oxidized mtDNA, which underscored ox-mtDNA as a critical DAMP that amplifies NLRP3-dependent neuroinflammation. Our work provides new insights into the numerous past studies that report the ameliorative effects of anti-oxidative stress on PD. Specifically, the mechanisms underlying these reductions in oxidative stress may involve the inhibition of mtDNA oxidation as well as the release of ox-mtDNA.

While previous studies have demonstrated that transfected mtDNA can bind to NLRP3 in Kupffer cells [44] and that ox-mtDNA interacts with NLRP3 in macrophages [15], its role in microglia, particularly in the context of PD, remains unexplored. This study demonstrated that treatment with LPS and rotenone-induced mtDNA-NLRP3 binding in BV2 cells, as evidenced by the detection of mtDNA in NLRP3 immunoprecipitates. Notably, the dot-blot analysis revealed significant oxidation of the co-precipitated DNA, suggesting that ox-mtDNA serves as the primary ligand. Molecular docking studies of ox-mtDNA and NLRP3 revealed a high-affinity binding interface within a positively charged, non-domain region connecting the PYD and NACHT domains. This finding aligns with Cabral et al.’s 2023 report of a direct interaction between oxidized DNA and NLRP3 [45], although its relevance in PD had not been previously established. We revealed that the segment from residues 180 to 187 within this highly positively charged domain constitutes the IDR of NLRP3 and is highly conserved. The IDR of the protein is intricately linked to its phase separation, which promotes NLRP3 aggregation and subsequent activation [46]. Despite its significance, the role of phase separation in NLRP3 activation has often been overlooked. A recent study has highlighted this mechanism; it was reported that cardiolipin, palmitate, and other NLRP3-binding molecules directly induce conformational changes and facilitate the phase separation of NLRP3 [46]. However, the potential binding of ox-mtDNA to the IDR region of NLRP3 remains unreported. Notably, EMSA confirmed that a truncated NLRP3 (180-1033) retaining this IDR bound to ox-mtDNA in a dose-dependent manner and triggered caspase-1 cleavage, confirming NLRP3 activation. Since D^2^P^2^ predicts IDR using a consensus of 10 algorithms (requiring > 75% agreement), some potential IDR may remain undetected, which could explain why the truncated NLRP3 (180-1033) exhibits slightly reduced ox-mtDNA binding compared to full-length NLRP3. However, it is undeniable that further truncation (188-1033) significantly weakened this binding. These findings implicate the IDR (residues 180-187) as critical for ox-mtDNA-mediated NLRP3 activation. Furthermore, given recent findings that NLRP3 activators (e.g., cardiolipin, palmitate) induce conformational changes and phase separation, we propose that the binding of ox-mtDNA to the IDR may similarly drive NLRP3 condensation, thereby amplifying inflammasome assembly. Future studies should investigate whether ox-mtDNA directly modulates NLRP3 phase separation to facilitate neurotoxic inflammation in PD.

It is important to acknowledge a limitation of our study that the primary in vitro findings were obtained using the BV2 cells line. We recognize that immortalized cell lines may not fully recapitulate the complexity of primary microglia. However, the central conclusions of our study—that ox-mtDNA is a key DAMP released from stressed microglia and directly activates the NLRP3 inflammasome via its IDR—are robustly supported by a convergence of evidence from multiple experimental systems. This includes key demonstrations in an in vivo mouse model, where microglial activation and subsequent neuronal damage were directly observed, and the core mechanism was validated using the NLRP3-specific inhibitor MCC950. The consistent observation of this pathway in vivo lends strong biological relevance to our findings. Nevertheless, future studies utilizing primary microglial cultures will be invaluable to further refine our understanding of the precise molecular dynamics involved.

In conclusion, our study reveals that ox-mtDNA serves as a crucial mediator in the activation of the NLRP3-IL-1β axis in microglia, contributing to the pathogenesis of PD. Given that ox-mtDNA directly interacts with the IDR region 180-187 of NLRP3 and activates NLRP3, the development of competitive inhibitors targeting this specific region may represent a promising therapeutic strategy to mitigate NLRP3 activation induced by microglial ox-mtDNA in response to neurotoxins, thereby potentially preventing the onset of PD.

## Materials and methods

### Cell culture and drug treatment

The BV2 cells and SH-SY5Y cells were obtained from Pricella (Wuhan, China). These cells were cultured in Dulbecco’s modified Eagle’s medium (Invitrogen, California, USA) supplemented with 10% FBS (Pricella) and 100 units/mL penicillin plus 100 µg/mL streptomycin (Invitrogen). Cells were cultured in a 37 °C incubator at 5% CO_2_. BV2 cells were first primed with 100 ng/mL LPS (Sigma-Aldrich, Missouri, USA) for 3 h, media was replaced, and cells were subsequently stimulated followed by treatment with rotenone (MedChemExpress, NJ, USA) at concentrations of 0.1 μM. BV2 cells were pretreated with 2 μM Cyclosporin A (CsA) (MedChemExpress) for 2 h before exposure to LPS and rotenone. After treatment, BV2 cells were either collected for mRNA extraction or for protein analysis by RT-qPCR or western blotting, respectively. The BCM was collected from treated BV2 cells, which were subsequently co-cultured with SH-SY5Y cells in fresh DMEM medium at a 1:1 ratio for 24 h [47]. The following reagents were obtained from commercial sources: MitoTracker Deep Red FM was from Beyotime (Nantong, Jiangsu, China); MitoSOX Red was from Invitrogen; Luminescent ATP Detection Assay Kit was from Abcam (Cambridge, UK).

### Animals and study design

Male C57BL/6J mice (18-22 g, 6-8 weeks) were obtained from Hangzhou Ziyuan Laboratory Animal Science and Technology Co., Ltd. The mice were randomly divided into groups (10 mice/group), and the investigators were blinded to the experiment performance. As illustrated in Fig. 1A, mtDNA was isolated from LPS-primed rotenone-induced BV2 cells, utilizing the mitochondrial fraction obtained as previously described [48]. LPS (4 µg/2 µL) or mtDNA (2 µg/2 µL) was injected into the left side of C57BL/6 mice in the Substantia Nigra (A/P − 2.2 mm, M/L 1.4 mm, D/V − 4.7 mm) relative to the bregma using a stereotactic instrument. Behavioral assessments were conducted for 7 days post-injection, followed by the collection of brains and blood for further analysis. As depicted in Fig. 3A, C57BL/6J mice were stereotactically injected with LPS as previously described and subsequently received intraperitoneal injections of rotenone (1.5 mg/kg) for 7 days. Finally, behavioral tests were conducted, and brains and blood were collected for further evaluation. All animal experimental protocols in the present study were operated according to the Guide for the Care and Use of Laboratory Animals published by the United States National Institutes of Health (NIH Publication No. 85-23, revised 1996) and were approved by the Experimental Animal Ethics Committee of the Zunyi Medical University (No. ZMU 21-2303-332). And we have complied with all relevant ethical regulations for animal use.

### Behavioral test

The open-field test evaluated the spontaneous locomotor activity of the animals. Mice underwent pre-training daily for three days prior to drug intervention. Before sampling, the mice were allowed to acclimate to the testing environment for 30 min without disturbance. Subsequently, they were placed in the center of the field and permitted to explore freely for 5 min. Their movements were recorded using video-tracking software, which monitored the activity for 5 min and calculated the total distance traveled and average speed.

### Isolation of neurons and microglia in midbrain

Single-cell suspensions were prepared from the midbrain of mice, following previously established protocols [49]. Neurons were isolated in accordance with the instructions provided by the Neuron Isolation Kit (Miltenyi Biotec, Bergisch Gladbach, Germany). Microglia were isolated according to the guidelines of the MojoSort™ Mouse P2RY12 Selection Kit (BioLegend, Inc., San Diego, CA, USA). The collected cells were assessed for purity, which was verified to exceed 90% through immunofluorescence analysis. Subsequently, transcriptome sequencing was conducted on the isolated cells.

### RNA extraction for transcriptomic sequencing

RNA extraction was conducted on neurons and microglia isolated from the brain tissues of three mice per group, utilizing TRIzol® Reagent (Takara, Kusatsu, Japan) according to the manufacturer’s guidelines. Following extraction, the samples were sent to Shanghai Majorbio Biopharm Technology Co., Ltd. for transcriptomic analysis. The quality of the RNA was assessed using the 5300 Bioanalyzer (Agilent) and quantified with the ND-2000 (NanoDrop Technologies). Ultimately, the RNA samples were used for the construction of sequencing libraries.

### Library preparation and sequencing

RNA purification, reverse transcription, library construction, and sequencing were performed according to the manufacturer’s guidelines (Illumina, San Diego, CA). The RNA-seq transcriptome library was generated using the SMART-Seq_V4 Ultra Low Input RNA Kit for Sequencing from Takara, starting with 10 ng of total RNA. Initially, reverse transcription, which involves the synthesis of a single strand, was conducted. During this process, RNA with a polyA tail (primarily mRNA) was reverse transcribed using an Oligo (dT) primer. Three cytosine (C) residues were added to the 3’ terminus of the cDNA strand due to the use of a specialized active reverse transcriptase (MMLVRT). Utilizing template-switching oligo (TSO) primers, two cDNA strands were synthesized, allowing for the replacement of RNA complementary to one of the cDNA strands. Subsequently, the cDNA was amplified to nanogram levels through PCR. A modified, highly active Tn5 transposase was employed for DNA fragmentation, and linkers were introduced at both ends of the cDNA. Following the final PCR amplification step, the library was prepared for sequencing. After quantification using Qubit 4.0, the sequencing library was processed on the NovaSeq X Plus platform (PE150) with the NovaSeq Reagent Kit.

### Quality control and read mapping

The raw paired-end reads were trimmed and quality-controlled using fastp with default parameters. Subsequently, the clean reads were aligned to the reference genome in orientation mode using HISAT2 software. The mapped reads for each sample were then assembled using StringTie in a reference-based approach for subsequent quantitative gene expression analysis.

### Principal component analysis (PCA)

PCA was performed to investigate and clarify the separation between neurons and microglia based on RNA-sequencing data, employing the princomp function in R.

### Differential expression analysis and functional enrichment

To identify DEGs between two distinct samples, the expression level of each transcript was calculated using the transcripts per million reads (TPM) method. RSEM was employed to quantify gene abundances. Differential expression analysis was conducted using DESeq2. DEGs with |log2FC | ≥ 1 and FDR < 0.05 (DESeq2) were considered significantly differentially expressed. Furthermore, functional enrichment analysis via KEGG was performed to determine which DEGs were significantly enriched in metabolic pathways, with a Bonferroni-corrected P-value < 0.05 compared to the whole transcriptome background. KEGG pathway analysis was executed using Python’s Scipy software. To investigate the pathways related to PD and the NLRP3 inflammasome, we extracted gene sets from the KEGG database using the following entries: mmu05012 (Parkinson’s disease pathway) and mmu04621 (NLRP3 inflammasome-related genes in the NOD-like receptor signaling pathway). Heatmaps were generated, displaying normalized read counts (Z-score) of significantly dysregulated genes within these pathways.

### Mitochondrial ROS assays

According to the manufacturer’s specifications, the mitochondrial ROS levels were evaluated by using MitoSOX Red (Thermo Fisher Scientific, Waltham, MA, USA). Cells posted by the specified treatments/interventions were incubated with the ROS fluorescent probe MitoSOX Red (500 nM) for 30 min and then washed thrice with PBS for 5 min each. Next, nuclei were stained with DAPI for 5 min, after which cells were washed thrice with PBS for 5 min each. Images were observed under a laser confocal microscope (Leica, Wetzlar, Germany).

### Immunofluorescence and confocal microscopy analysis

The cells were placed in 24-well plates with glass slides for 24 h. Upon treatment, the cells were fixed with 4% paraformaldehyde for 15 min and permeabilized with 0.1% Triton (Solarbio, Beijing, China) for 10 min at room temperature. Then, the cells were blocked with goat serum (Zhongshan Golden Bridge Biotechnology, Beijing, China) for 30 min at room temperature and incubated with primary antibodies overnight at 4 °C. After washing with PBS, the cells were incubated with Fluor secondary fluorescent antibodies (Proteintech, Wuhan, China) for 1 h at room temperature and stained with DAPI (Solarbio).

The cells were seeded in 24-well plates containing glass slides for a duration of 24 hours to facilitate the detection of cytoplasmic DNA or mitochondria. Following treatment, the cells were incubated with MitoTracker Deep Red FM for 30 minutes at 37 °C. Subsequently, after washing with PBS, the cells were fixed using 4% paraformaldehyde for 15 minutes and permeabilized with 0.2% Triton for 20 minutes at room temperature. The cells were then incubated with anti-ss/dsDNA antibody (Santa Cruz Biotechnology, Dallas, TX, USA) for 16 hours at 4 °C. Finally, the cells were treated with secondary fluorescent antibodies for 1 hour at room temperature and subsequently stained with DAPI. The paraffin tissue sections were dewaxed and citrate repaired followed by goat serum blocking for 30 min. A primary antibody was added to cover the tissues at 4 °C overnight. Then, the sections were washed with PBS three times, 5 min each time. Secondary fluorescent antibodies were dripped onto the sections until covered completely, then incubated them at room temperature for 2 h. The secondary antibodies were washed with PBS, and DAPI was dripped. Images were captured using orthogonal fluorescence microscope (Leica), inverted fluorescence microscope (Leica) or laser confocal microscope (Leica). Analysis of the image. The secondary only controls for the ICC staining are shown in Fig. S8A–E.

### Immunoprecipitation

To detect the binding of mtDNA to NLRP3 in BV2 cells, the cells were initially seeded in a 15 cm cell culture dish. Then, formaldehyde was added to a final concentration of 4% for 10 min at room temperature to cross-link, and glycine (2.5 M) was added to terminate the cross-link. Next, they were lysed using 500 µl of lysis buffer (Absin Bioscience, Shanghai, China) containing 1 mM PMSF and centrifuged at 14000 ×g for 10 min at 4 °C. The resulting supernatants were collected, and the concentrations of total proteins were determined using BCA kits. Samples with equal protein concentrations were transferred to fresh 1.5 mL Eppendorf centrifuge tubes and incubated with antibody against NLRP3 at 4 °C for 16 h. Protein A + G Agarose beads, which had been prewashed three times with wash buffer, were added to the tubes, which were then incubated for 16 h at 4 °C. The immune complexes are then washed. After washing, DNA was eluted in 1% SDS, 100 mM NaHCO3, cross-link was reversed with NaCl and DNA was purified and assessed by qPCR or dot-blot. Immunoblotting was performed using mouse antibody against 8OH-dG (Rockland Immunochemicals Inc., Limerick, PA, USA). The primer sequences provided are shown in Supplementary Table 1.

### Lentiviral transfection

The NLRP3-interfering lentivirus (NLRP3-shRNA) and the corresponding negative control virus were synthesized by Obio Technology (Shanghai, China). NLRP3-shRNA consisted of the following sequence: CCCGGACTGTAAACTACAGAT. After 24 h, the original medium was removed; the fresh medium and an appropriate amount of virus solution (MOI: 20) were added to 1 mL per well. The transfected virus was an shNC lentivirus and a shNLRP3 lentivirus, which was then cultured in an incubator. After about 16 h of culture, the cells were monitored, and the fresh medium was changed for further culture. About 72 h after infection, the efficiency of infection was observed using a fluorescence microscope. BV2 cells grew well after infection, and 3 µg/mL puromycin (Beyotime) was added for cell screening. After 6 days of screening, western blotting was conducted to determine the transfection efficiency of shNLRP3 in BV2 cells. The fresh complete medium was changed to allow the surviving cells to proliferate and freeze for subsequent experiments.

### Extraction of mtDNA

To isolate mtDNA, BV2 cells were treated with LPS and rotenone prior to collection. The cells were harvested via trypsinization, washed with PBS, and subjected to mild detergent-based lysis using 1% NP-40 on ice for 10 minutes. Subsequently, the lysates were centrifuged at 17,000 ×g for 10 minutes at 4 °C to separate the nuclear pellet from the cytoplasmic supernatant. Finally, mtDNA was purified from the supernatant through enzymatic digestion with RNase A and proteinase K, which effectively removed RNA and protein contaminants, respectively.

### Extraction of cytoplasmic mtDNA

mtDNA in the cytosolic fraction was detected by cell compartment fractionation followed by qPCR [50]. Briefly, half of the cells were lysed by mild detergent digitonin (20 nM) and incubated on ice for 10 min. Lysates were centrifuged at 950 ×g for 5 min at 4 °C and then centrifuged at 17 000 ×g for 5 min to obtain the cytoplasmic mtDNA (supernatant). The other half of the cells were lysed with 10% SDS lysate at 95 °C for 15 min and sonicated to extract whole cell DNA. The midbrain isolated the cytoplasmic portion of the DNA and the nucleus according to the previous methods [51]. DNARun denatured and reduced protein extract on SDS polyacrylamide mini gels and performed western blotting. Antibodies against GAPDH or Tubulin (cytosolic extract), TFAM (mitochondrial extract), and Lamin B (nuclear extract) were used for immunoblotting to assess purity. Phenol, chloroform and isopropanol were used to purify DNA. For SYBR Green-based qPCR analysis, calculate mtDNA abundance relative to nuclear DNA using the delta delta Cq (ΔΔCq) method. Specific primer sequences for all genes are shown in Supplementary Table 1.

### mtDNA synthesis and oxidation

The 90 bp D-loop mtDNA was synthesized by Sangon Biotech (Shanghai, China) [45], and the specific sequence is provided in Supplementary Table 1. The synthesized mtDNA was oxidized by treatment with H_2_O_2_ (88 mM) for 3 h at 37 °C. A dot blotting assay, incubated with antibody against 8OHdG, was employed to confirm the oxidation of mtDNA, while agarose gel electrophoresis was utilized to ensure that this oxidation did not lead to any further degradation of the mtDNA.

### mtDNA transfection

The BV2 cells were initially primed with LPS, as previously described. Following this, mtDNA extracted from LPS + rotenone-treated BV2 cells, or synthetic oxidized or non-oxidized mtDNA (1 μg/mL), was transfected using Lipofectamine 2000 (Invitrogen) in accordance with the manufacturer’s protocol.

### Analysis of protein expression

Primary antibodies against TH (1:2000, #58844, CST, Danvers, MA, USA), NLRP3 (1:1000, #15101, CST), IL-1β (1:1000, #31202, CST), cleaved-caspase-1 (1:1000, #83383, CST), TFAM (1:1000, #sc-166965, Santa Cruz), Lamin B (1:5000, #66095-1-Ig, Proteintech),α-Tubulin (1:1000, #14555-1-AP, Proteintech) and GAPDH (1:1000, #60004-1-Ig, Proteintech) were used for Western blotting and performed as previously described [52]. Original blot pictures are shown in the Supplementary Material. Band intensities were quantified using NIH ImageJ software.

### Real-time quantitative polymerase chain reaction analysis

Total RNA was prepared from BV2 cells and mouse midbrain samples using TRIzol® reagent (Takara) according to the manufacturer’s instructions. Total RNA content and purity were measured by Ultra Micro Spectrophotometer (Thermo Fisher Scientific). cDNA was synthesized with 1 µg RNA using the Prime Script™ RT Reagent Kit (Takara). The real-time qPCR apparatus (Bio-Rad, Hercules, CA, USA) was used for PCR amplify-cation. The GAPDH was used as the internal reference gene for data normalization. Specific primer sequences for all genes are shown in Supplementary Table 1.

### Immunohistochemistry

After the animals were sacrificed, the midbrain was collected and immersed in 10% neutral formaldehyde for fixation. The tissues containing the SNpc were dehydrated, paraffin-embedded, and sectioned into serial coronal sections. Subsequently, the sections were deparaffinized, hydrated, and incubated with the antibody against TH (1:5000, #25859-1-AP, Proteintech) for immunohistochemical staining using an immunohistochemistry kit (Zhongshan Golden Bridge). The secondary only controls for the IHC staining are shown in Fig. S8F.

### Dot blotting

DNA samples (50 ng to 400 ng in 0.5 to 1 μL) were spotted onto a nitrocellulose membrane. The membrane was air-dried for 10 min and subsequently UV-cross-linked (120 mJ/cm²) for 30 s to immobilize the DNA. Following this, the membranes were incubated with 5% skimmed milk at 4 °C for 2 h to block non-specific binding. Afterward, they were incubated at room temperature for 2 h with a primary antibody against 8-OH-dG (1:1000, #200-301-A99, Rockland). The subsequent steps were conducted in accordance with standard Western blotting protocols.

### Molecular structure and molecular docking

The three-dimensional conformation of mitochondrial DNA was predicted using the 3dDNA online program. Subsequently, we utilized Chimera software to substitute the hydrogen atom at position C8 on the guanine base of mtDNA with a hydroxyl group, followed by structure optimization to obtain the three-dimensional conformation of ox-mtDNA. The predominant bindings conformation of ox-mtDNA or ox-DNA (PDB 3I0W) to NLRP3 (PDB 7PZC) were further predicted using the Z-dock program, and the docking results and the surface charge distribution of NLRP3 were visualized using PyMOL software. The dominant conformation PDB file was uploaded to the PDBePISA website, where the system automatically analyzed the intermolecular interaction parameters.

### Analysis of intrinsically disordered regions across NLRP3

The D^2^P^2^ Database of Disordered Protein Predictions analyzed intrinsically disordered regions (IDR) of NLRP3. By searching the Uniport database and selecting the BLAST feature, FASTA file of the protein sequence of NLRP3 was compiled. Then, it was inputted into the D^2^P^2^ “Match Amino Acid Sequence” protein search tool. This search returned to a matched sequence for NLRP3. The result provided maps incorporating identification of the type of predicted disorder. These maps were compared to one another to identify where most IDRs were predicted to be, and a region is labeled as a high-confidence IDR only when ≥ 75% of the algorithms consistently predict that the region is disordered.

### Sequence alignment

The sequence comparison of NLRP3 from various species was conducted using Clustal Omega software. The symbols ‘*’, ‘:’, and ‘.’ beneath the sequences indicate the conservation levels of the amino acid residues at corresponding positions. Specifically, ‘*’ denotes full conservation, ‘:’ indicates strong conservation, and ‘.’ signifies weak conservation among all sequences analyzed, which means that the amino acid at that position is either identical or similar in nature across all compared sequences.

### Protein expression and purification

As shown in Fig. S7A–D, the full-length mouse NLRP3 (NLRP3-FL, residues 1-1033), along with its N-terminal truncated variants truncated NLRP3 (180-1033) and truncated NLRP3 (188-1033), as well as the PYD-caspase-1 fusion protein, which incorporates the PYD of ASC in place of their original CARD, were cloned into an RN161 vector. This vector contains an N-terminal His-sumo tag and 5’ BamHI and 3’ XhoI restriction sites for prokaryotic expression. The constructs were transformed into E. coli BL21(DE3) cells and cultured in LB medium at 37 °C until the optical density at 600 nm (OD_600_) reached 0.6–0.8. Protein expression was then induced with 0.1 mM IPTG at 37 °C for 4 h, followed by 6 h at 22 °C. Cells were harvested by centrifugation (5000 ×g, 5 min, 4 °C), resuspended in lysis buffer (25 mM Tris, 500 mM NaCl, pH 7.4), and lysed using TieChui™ E. coli Lysis Buffer. The proteins were washed twice with wash buffer (TBS, 1% Triton X-100, pH 7.4) to eliminate membrane contaminants, followed by a final wash with lysis buffer devoid of detergent. The purified proteins were then solubilized in 8 M urea. For refolding, the solubilized proteins were gradually dialyzed against TBS buffer (pH 7.4) to remove urea. The refolded proteins were then purified by nickel affinity chromatography using Smart-NI resin. The column was equilibrated with TBS buffer (pH 7.4), and bound proteins were eluted with a linear gradient of imidazole (2 mM, 20 mM, and 250 mM) in TBS buffer (pH 7.4). The purity of the eluted proteins was confirmed to be >85% by SDS-PAGE (Fig. S7A–D). Eluted fractions were aliquoted and stored at -80 °C for subsequent experiments.

### EMSA and agarose gel electrophoresis

In this experiment, 1-4 μg of each protein was incubated with 1 μL of 1 μg of ox-mtDNA for 20 min at 37 °C. During this incubation, a 2% agarose gel was pre-run in TBE buffer for 30 min at 110 V. Subsequently, the samples were loaded onto the gel, which was then run for an additional 30 min at the same voltage.

### In vitro PYD-caspase-1 cleavage assays

Purified truncated NLRP3 (180-1033) was incubated with ox-mtDNA together with PYD-casepase-1 (8 μg) in PBS for 30 min at 37 °C. The reaction mixtures were then fractionated by SDS-PAGE and analyzed by western blotting with cleaved-caspase-1 antibody.

### Statistical analysis

All data are presented as individual points overlaid with mean ± SEM. Normality was assessed by Shapiro-Wilk test (α = 0.05). Parametric analyses were applied to normally distributed datasets (*p* > 0.05): Student’s t-test for two-group comparisons or one-way ANOVA for multi-group comparisons. Tukey’s post hoc test was used for analysis of data meeting the homogeneity of variance assessment, or Games Howell analysis for heterogeneous data was used for data from multiple groups if they followed a normal distribution. Non-normal data (*p* < 0.05) were analyzed using nonparametric equivalents. Statistical analyses and graphing utilized SPSS (version 29.0, SPSS Inc., USA) and GraphPad Prism (version 9.4.1, GraphPad Inc., USA), respectively. Statistical significance was defined as *p* < 0.05. Sample sizes and biological replicates are specified in figure legends.

## Supplementary information

Supplementary Figures

Supplementary table 1

Original western blots

## Data Availability

The data that support the findings of this study are available from the corresponding author [szhou@zmu.edu.cn] upon reasonable request.

## References

[1] Tolosa E, Garrido A, Scholz SW, Poewe W. Challenges in the diagnosis of Parkinson’s disease. Lancet Neurol. 2021;20:385–97. 33894193 10.1016/S1474-4422(21)00030-2 PMC8185633

[2] Zhang D, Li S, Hou L, Jing L, Ruan Z, Peng B, et al. Microglial activation contributes to cognitive impairments in rotenone-induced mouse Parkinson’s disease model. J Neuroinflammation. 2021;18:4. 33402167 10.1186/s12974-020-02065-z PMC7786472

[3] Zhao W, Liu Z, Wu J, Liu A, Yan J. Potential targets of microglia in the treatment of neurodegenerative diseases: mechanism and therapeutic implications. Neural Regen Res. 2026;21:1497–511. 40145977 10.4103/NRR.NRR-D-24-01343 PMC12407519

[4] Soraci L, Gambuzza ME, Biscetti L, Laganà P, Lo Russo C, Buda A, et al. Toll-like receptors and NLRP3 inflammasome-dependent pathways in Parkinson’s disease: mechanisms and therapeutic implications. J Neurol. 2023;270:1346–60. 36460875 10.1007/s00415-022-11491-3 PMC9971082

[5] Gu YY, Zhao XR, Zhang N, Yang Y, Yi Y, Shao QH, et al. Mitochondrial dysfunction as a therapeutic strategy for neurodegenerative diseases: current insights and future directions. Ageing Res Rev. 2024;102:102577. 39528070 10.1016/j.arr.2024.102577

[6] Wright R. Mitochondrial dysfunction and Parkinson’s disease. Nat Neurosci. 2022;25:2. 34992288 10.1038/s41593-021-00989-0

[7] Flønes IH, Toker L, Sandnes DA, Castelli M, Mostafavi S, Lura N, et al. Mitochondrial complex I deficiency stratifies idiopathic Parkinson’s disease. Nat Commun. 2024;15:3631. 38684731 10.1038/s41467-024-47867-4 PMC11059185

[8] Takeuchi H, Mizuno T, Zhang G, Wang J, Kawanokuchi J, Kuno R, et al. Neuritic beading induced by activated microglia is an early feature of neuronal dysfunction toward neuronal death by inhibition of mitochondrial respiration and axonal transport. J Biol Chem. 2005;280:10444–54. 15640150 10.1074/jbc.M413863200

[9] Tresse E, Marturia-Navarro J, Sew WQG, Cisquella-Serra M, Jaberi E, Riera-Ponsati L, et al. Mitochondrial DNA damage triggers spread of Parkinson’s disease-like pathology. Mol Psychiatry. 2023;28:4902–14. 37779111 10.1038/s41380-023-02251-4 PMC10914608

[10] West AP, Shadel GS. Mitochondrial DNA in innate immune responses and inflammatory pathology. Nat Rev Immunol. 2017;17:363–75. 28393922 10.1038/nri.2017.21 PMC7289178

[11] Marchi S, Guilbaud E, Tait SWG, Yamazaki T, Galluzzi L. Mitochondrial control of inflammation. Nat Rev Immunol. 2023;23:159–73. 35879417 10.1038/s41577-022-00760-x PMC9310369

[12] Maatouk L, Compagnion A-C, Sauvage M-AC-d, Bemelmans A-P, Leclere-Turbant S, Cirotteau V, et al. TLR9 activation via microglial glucocorticoid receptors contributes to degeneration of midbrain dopamine neurons. Nat Commun. 2018;9:2450. 29934589 10.1038/s41467-018-04569-y PMC6015079

[13] Gulen MF, Samson N, Keller A, Schwabenland M, Liu C, Glück S, et al. cGAS–STING drives ageing-related inflammation and neurodegeneration. Nature. 2023;620:374–80. 37532932 10.1038/s41586-023-06373-1 PMC10412454

[14] Zhong Z, Liang S, Sanchez-Lopez E, He F, Shalapour S, Lin XJ, et al. New mitochondrial DNA synthesis enables NLRP3 inflammasome activation. Nature. 2018;560:198–203. 30046112 10.1038/s41586-018-0372-z PMC6329306

[15] Shimada K, Crother TR, Karlin J, Dagvadorj J, Chiba N, Chen S, et al. Oxidized mitochondrial DNA activates the NLRP3 inflammasome during apoptosis. Immunity. 2012;36:401–14. 22342844 10.1016/j.immuni.2012.01.009 PMC3312986

[16] Haque ME, Akther M, Jakaria M, Kim IS, Azam S, Choi DK. Targeting the microglial NLRP3 inflammasome and its role in Parkinson’s disease. Mov Disord. 2020;35:20–33. 31680318 10.1002/mds.27874

[17] Yan YQ, Zheng R, Liu Y, Ruan Y, Lin ZH, Xue NJ, et al. Parkin regulates microglial NLRP3 and represses neurodegeneration in Parkinson’s disease. Aging cell. 2023;22:e13834. 37029500 10.1111/acel.13834 PMC10265164

[18] Szeto HH, Liu S, Soong Y, Seshan SV, Cohen-Gould L, Manichev V, et al. Mitochondria protection after acute ischemia prevents prolonged upregulation of IL-1β and IL-18 and arrests CKD. J Am Soc Nephrol. 2017;28:1437–49. 27881606 10.1681/ASN.2016070761 PMC5407729

[19] Wu Y, Hao C, Liu X, Han G, Yin J, Zou Z, et al. MitoQ protects against liver injury induced by severe burn plus delayed resuscitation by suppressing the mtDNA-NLRP3 axis. Int Immunopharmacol. 2020;80:106189. 31931374 10.1016/j.intimp.2020.106189

[20] Xu W, Huang Y, Zhou R. NLRP3 inflammasome in neuroinflammation and central nervous system diseases. Cell Mol Immunol. 2025;22:341–55. 40075143 10.1038/s41423-025-01275-w PMC11955557

[21] Li T, Tan X, Tian L, Jia C, Cheng C, Chen X, et al. The role of Nurr1-miR-30e-5p-NLRP3 axis in inflammation-mediated neurodegeneration: insights from mouse models and patients’ studies in Parkinson’s disease. J Neuroinflammation. 2023;20:274. 37990334 10.1186/s12974-023-02956-x PMC10664369

[22] Ou Z, Zhou Y, Wang L, Xue L, Zheng J, Chen L, et al. NLRP3 inflammasome inhibition prevents α-synuclein pathology by relieving autophagy dysfunction in chronic MPTP-treated NLRP3 knockout mice. Mol Neurobiol. 2021;58:1303–11. 33169332 10.1007/s12035-020-02198-5

[23] Kong L, Li W, Chang E, Wang W, Shen N, Xu X, et al. mtDNA-STING axis mediates microglial polarization via IRF3/NF-κB signaling after ischemic stroke. Front Immunol. 2022;13:860977. 35450066 10.3389/fimmu.2022.860977 PMC9017276

[24] Guan X, Zhu S, Song J, Liu K, Liu M, Xie L, et al. Microglial CMPK2 promotes neuroinflammation and brain injury after ischemic stroke. Cell Rep Med. 2024;5:101522. 38701781 10.1016/j.xcrm.2024.101522 PMC11148565

[25] Liang Z, Damianou A, Vendrell I, Jenkins E, Lassen FH, Washer SJ, et al. Proximity proteomics reveals UCH-L1 as an essential regulator of NLRP3-mediated IL-1β production in human macrophages and microglia. Cell Rep. 2024;43:114152. 38669140 10.1016/j.celrep.2024.114152

[26] Morton KS, George AJ, Meyer JN. Complex I superoxide anion production is necessary and sufficient for complex I inhibitor-induced dopaminergic neurodegeneration in Caenorhabditis elegans. Redox Biol. 2025;81:103538. 39952197 10.1016/j.redox.2025.103538 PMC11875150

[27] Norat P, Soldozy S, Sokolowski JD, Gorick CM, Kumar JS, Chae Y, et al. Mitochondrial dysfunction in neurological disorders: exploring mitochondrial transplantation. NPJ Regen Med. 2020;5:22. 33298971 10.1038/s41536-020-00107-x PMC7683736

[28] Pérez-Treviño P, Velásquez M, García N. Mechanisms of mitochondrial DNA escape and its relationship with different metabolic diseases. Biochim Biophys Acta Mol Basis Dis. 2020;1866:165761. 32169503 10.1016/j.bbadis.2020.165761

[29] Roy T, Chatterjee A, Swarnakar S. Rotenone induced neurodegeneration is mediated via cytoskeleton degradation and necroptosis. Biochim Biophys Acta Mol Cell Res. 2023;1870:119417. 36581087 10.1016/j.bbamcr.2022.119417

[30] Ravanat JL, Di Mascio P, Martinez GR, Medeiros MH, Cadet J. Singlet oxygen induces oxidation of cellular DNA. J Biol Chem. 2000;275:40601–4. 11007783 10.1074/jbc.M006681200

[31] Alexeyev M, Shokolenko I, Wilson G, LeDoux S. The maintenance of mitochondrial DNA integrity-critical analysis and update. Cold Spring Harb Perspect Biol. 2013;5:a012641. 23637283 10.1101/cshperspect.a012641 PMC3632056

[32] Marques E, Kramer R, Ryan DG. Multifaceted mitochondria in innate immunity. NPJ Metab Health Dis. 2024;2:6. 38812744 10.1038/s44324-024-00008-3 PMC11129950

[33] Jonas F, Navon Y, Barkai N. Intrinsically disordered regions as facilitators of the transcription factor target search. Nat Rev Genet. 2025;26:424–35. 39984675 10.1038/s41576-025-00816-3

[34] Guo B, Gu J, Zhuang T, Zhang J, Fan C, Li Y, et al. MicroRNA-126: from biology to therapeutics. Biomed Pharmacother. 2025;185:117953. 40036996 10.1016/j.biopha.2025.117953

[35] Sarkar S, Malovic E, Harishchandra DS, Ghaisas S, Panicker N, Charli A, et al. Mitochondrial impairment in microglia amplifies NLRP3 inflammasome proinflammatory signaling in cell culture and animal models of Parkinson’s disease. NPJ Parkinsons Dis. 2017;3:30. 29057315 10.1038/s41531-017-0032-2 PMC5645400

[36] Jankovic J, Tan EK. Parkinson’s disease: etiopathogenesis and treatment. J Neurol Neurosurg Psychiatry. 2020;91:795–808. 32576618 10.1136/jnnp-2019-322338

[37] Ni J, Wu Z, Meng J, Saito T, Saido TC, Qing H, et al. An impaired intrinsic microglial clock system induces neuroinflammatory alterations in the early stage of amyloid precursor protein knock-in mouse brain. J Neuroinflammation. 2019;16:173. 31470863 10.1186/s12974-019-1562-9 PMC6716829

[38] Lawana V, Singh N, Sarkar S, Charli A, Jin H, Anantharam V, et al. Involvement of c-Abl kinase in microglial activation of NLRP3 inflammasome and impairment in autolysosomal system. J Neuroimmune Pharmacol. 2017;12:624–60. 28466394 10.1007/s11481-017-9746-5 PMC5668207

[39] Li T, Li Y, Chen J, Nan M, Zhou X, Yang L, et al. Hyperibone J exerts antidepressant effects by targeting ADK to inhibit microglial P2X7R/TLR4-mediated neuroinflammation. J Adv Res. 2025;72:571–89. 39019111 10.1016/j.jare.2024.07.015 PMC12147645

[40] Pan-Montojo F, Anichtchik O, Dening Y, Knells L, Pursche S, Jung R, et al. Progression of Parkinson’s disease pathology is reproduced by intragastric administration of rotenone in mice. PLoS ONE. 2010;5:e8762. 20098733 10.1371/journal.pone.0008762 PMC2808242

[41] Pfeifer GP. DNA damage and Parkinson’s disease. Int J Mol Sci. 2024;25:4187. 38673772 10.3390/ijms25084187 PMC11050701

[42] Cao B, Wang T, Qu Q, Kang T, Yang Q. Long noncoding RNA SNHG1 promotes neuroinflammation in Parkinson’s disease via regulating miR-7/NLRP3 pathway. Neuroscience. 2018;388:118–27. 30031125 10.1016/j.neuroscience.2018.07.019

[43] Zhang W, Li G, Luo R, Lei J, Song Y, Wang B, et al. Cytosolic escape of mitochondrial DNA triggers cGAS-STING-NLRP3 axis-dependent nucleus pulposus cell pyroptosis. Exp Mol Med. 2022;54:129–42. 35145201 10.1038/s12276-022-00729-9 PMC8894389

[44] Pan J, Ou Z, Cai C, Li P, Gong J, Ruan XZ, et al. Fatty acid activates NLRP3 inflammasomes in mouse Kupffer cells through mitochondrial DNA release. Cell Immunol. 2018;332:111–20. 30103942 10.1016/j.cellimm.2018.08.006

[45] Cabral A, Cabral JE, Wang A, Zhang Y, Liang H, Nikbakht D, et al. Differential binding of NLRP3 to non-oxidized and Ox-mtDNA mediates NLRP3 inflammasome activation. Commun Biol. 2023;6:578. 37253813 10.1038/s42003-023-04817-y PMC10229695

[46] Zou G, Tang Y, Yang J, Fu S, Li Y, Ren X, et al. Signal-induced NLRP3 phase separation initiates inflammasome activation. Cell Res. 2025;35:437–52. 40164768 10.1038/s41422-025-01096-6 PMC12134225

[47] Zhang Q, Zhou J, Shen M, Xu H, Yu S, Cheng Q, et al. Pyrroloquinoline quinone inhibits rotenone-induced microglia inflammation by enhancing autophagy. Molecules. 2020;25:4359. 32977419 10.3390/molecules25194359 PMC7582530

[48] Shen Y, Wang X, Nan N, Fu X, Zeng R, Yang Y, et al. SIRT3-mediated deacetylation of SDHA rescues mitochondrial bioenergetics contributing to neuroprotection in rotenone-induced PD models. Mol Neurobiol. 2024;61:4402–20. 38087172 10.1007/s12035-023-03830-w

[49] Zhang W, Zhang M, Wu Q, Shi J. Dendrobium nobile Lindl. Alkaloids ameliorate Aβ25-35-induced synaptic deficits by targeting Wnt/β-catenin pathway in Alzheimer’s disease models. J Alzheimers Dis. 2022;86:297–313. 35068466 10.3233/JAD-215433

[50] Bryant JD, Lei Y, VanPortfliet JJ, Winters AD, West AP. Assessing mitochondrial DNA release into the cytosol and subsequent activation of innate immune-related pathways in mammalian cells. Curr Protoc. 2022;2:e372. 35175686 10.1002/cpz1.372 PMC8986093

[51] Jiménez-Loygorri JI, Villarejo-Zori B, Viedma-Poyatos Á, Zapata-Muñoz J, Benítez-Fernández R, Frutos-Lisón MD, et al. Mitophagy curtails cytosolic mtDNA-dependent activation of cGAS/STING inflammation during aging. Nat Commun. 2024;15:830. 38280852 10.1038/s41467-024-45044-1 PMC10821893

[52] Wang Y, Fu X, Zeng L, Hu Y, Gao R, Xian S, et al. Activation of Nrf2/HO-1 signaling pathway exacerbates cholestatic liver injury. Commun Biol. 2024;7:621. 38783088 10.1038/s42003-024-06243-0 PMC11116386

