# Supplementary material for: Mitochondrial DNA drives NLRP3-IL-1β axis activation in microglia by binding to NLRP3, leading to neurodegeneration in Parkinson’s disease models
Source: Cell Death Dis. 2026 Feb 10;17(1):213. doi: 10.1038/s41419-026-08424-7 (PMC12921273; doi:10.1038/s41419-026-08424-7)
Supplement: Supplementary file 1 — Supplementary Figures [file 41419_2026_8424_MOESM1_ESM.docx]

# Mitochondrial DNA drives NLRP3-IL-1β axis activation in microglia by binding to NLRP3, leading to neurodegeneration in Parkinson’s disease models

Qinglin Gan^1, 3^, Xiaolong Fu^1, 3^, Ting Zhou^1^, Naiyu Fan^1^, Nan Nan^1^, Yi Wang^2^, Yonggang Yang^1^, Shiyi Gou^1^, Lizhen Hu^1^, Shaoyu Zhou^1, *^

^1^Key Laboratory of Basic Pharmacology of Ministry of Education and Joint International Research Laboratory of Ethnomedicine of Ministry of Education, Zunyi Medical University, Zunyi, Guizhou Province, China.

^2^Jinsha County People’s Hospital, Bijie, Guizhou Province, China.

^3^These authors contributed equally: Qinglin Gan and Xiaolong Fu.

**Fig. S1**


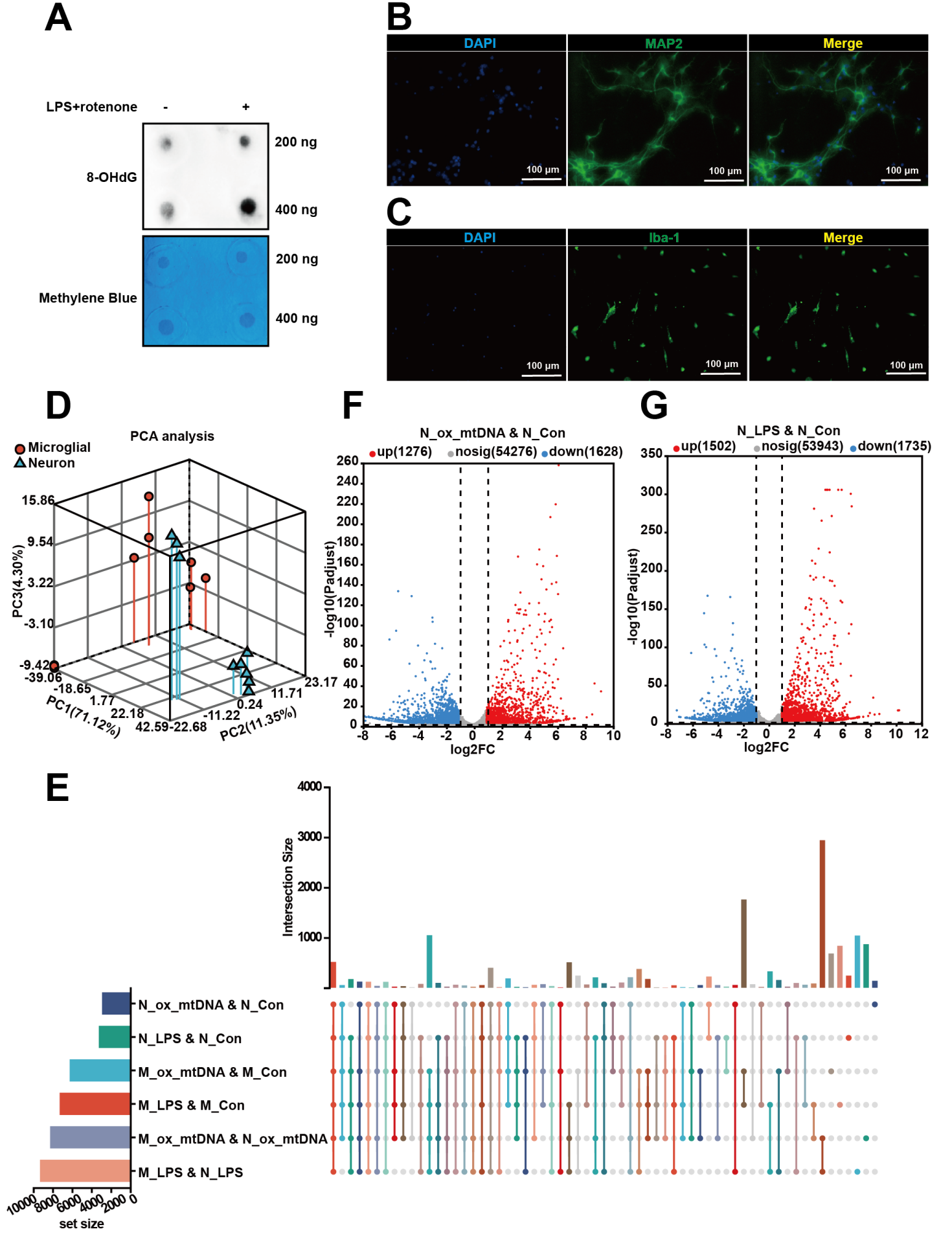


**Fig. S1 Stereotactic injection of ox-mtDNA elicits distinct transcriptomic profiles in midbrain neurons and microglia. A** The oxidation of mtDNA was detected using anti-8-OHdG antibody in LPS (100 ng/mL) primed rotenone (0.1 μM) stimulated BV2 cells. **B** Images showing immunostaining of Neurons isolated by the Neuron Isolation Kit Microglia for MAP2. Scale bar = 100 μm (n = 3). **C** Images showing immunostaining of microglia isolated by the MojoSort™ Mouse P2RY12 Selection Kit for Iba-1. Scale bar = 100 μm. **D** Principal Component Analysis (PCA) of RNA-seq data from mice neurons and microglia with or without LPS (4 µg/2 µL) or ox-mtDNA (2 µg/2 µL) treatment (n = 3). **E** The comparison of the number and MA plot of DEGs between mouse neurons LPS (4 µg/2 µL) & Con groups (n = 3). **F** The comparison of the number and Volcano plot of DEGs between mouse neurons ox-mtDNA (2 µg/2 µL) & Con groups (n = 3). **G** UpSet plot showing intersection relationships of DEGs in 6 comparisons.

**Fig.S2**

**
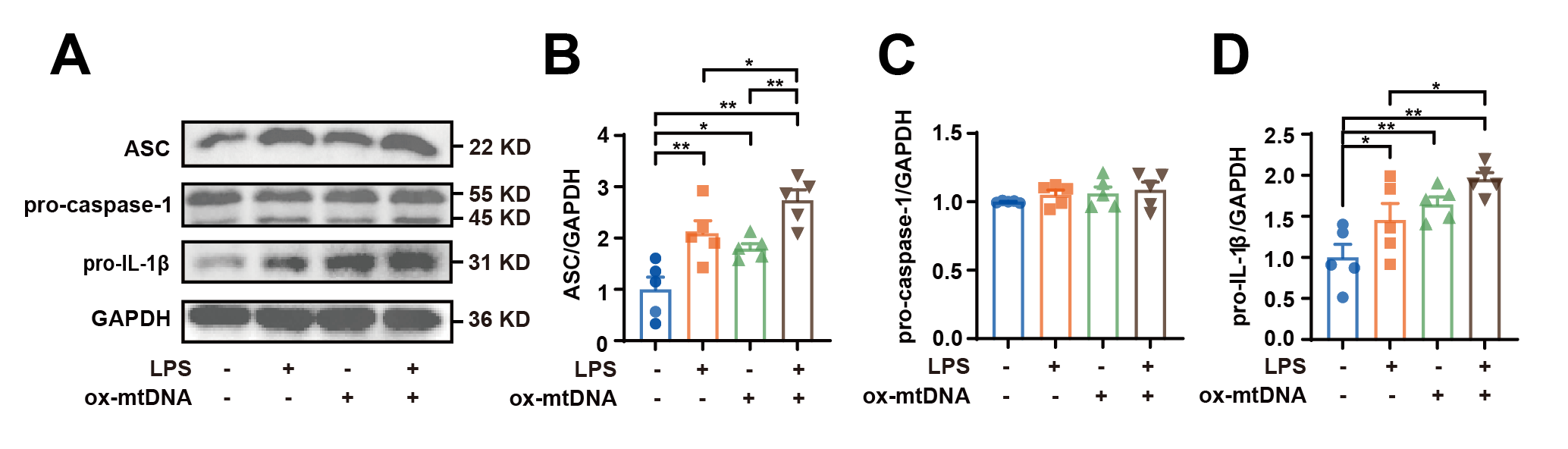
**

**Fig. S2 The expression of ASC, pro-caspase-1 and IL-1β in the LPS-primed ox-mtDNA-induced mouse midbrain. A-D** ASC, pro-caspase-1 and pro-IL-1β protein expressions in LPS (4 µg/2 µL) primed ox-mtDNA (2 µg/2 µL) induced mice midbrain were measured by [western blotting](https://www.sciencedirect.com/topics/pharmacology-toxicology-and-pharmaceutical-science/western-blot) (n = 5). Data are presented as mean ± SEM. ^*^*p* < 0.05, ^**^ *p* < 0.01, and ^***^ *p* < 0.001.

**Fig. S3**


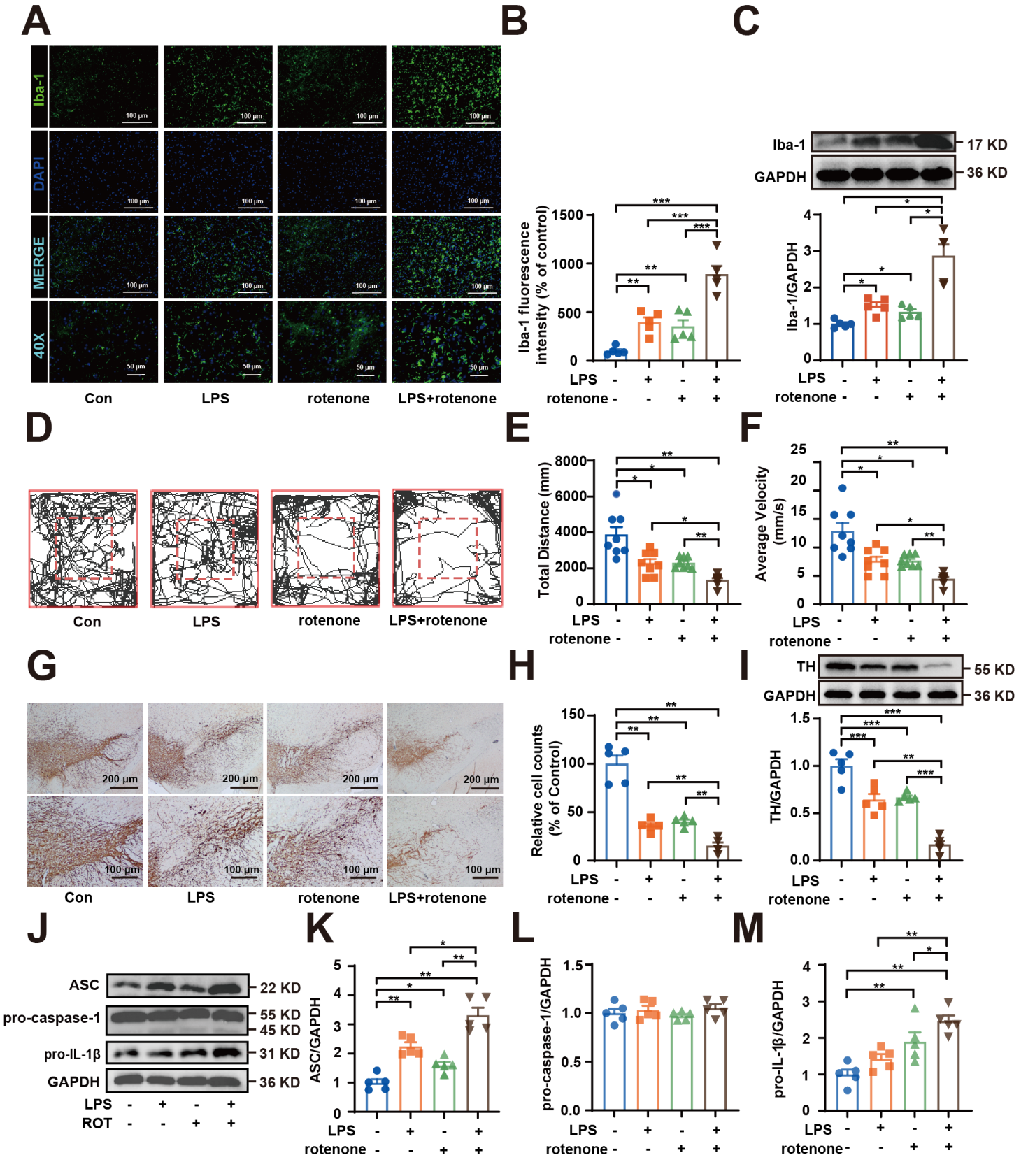


**Fig. S3 LPS primed and rotenone induced pathological changes in PD and upregulation of the NLRP3 inflammasome signaling pathway. A and B** Immunofluorescence [staining](https://www.sciencedirect.com/topics/neuroscience/staining-technique) and quantification of the fluorescence intensity of Iba-1 (green) in the midbrain were determined (n = 5). Scale bar = 100 μm (top), 50 μm (bottom). **C** [Iba-1](https://www.sciencedirect.com/topics/neuroscience/tyrosine-hydroxylase) protein level in the midbrain was examined by [western blotting](https://www.sciencedirect.com/topics/medicine-and-dentistry/western-blot) (n = 5). **D** Representative traces of total distance traveled in the open field test (n = 8). **E** The total distance travelled and (**F)** the average velocity (n = 8). **G and H** Immunohistochemical [staining](https://www.sciencedirect.com/topics/neuroscience/staining-technique) and quantification of TH-positive neurons in [SN](https://www.sciencedirect.com/topics/medicine-and-dentistry/substantia-nigra) were determined (n = 5). Scale bar = 200 μm (top), 100 μm (bottom). **I** [TH](https://www.sciencedirect.com/topics/neuroscience/tyrosine-hydroxylase) protein level in the midbrain was examined by [western blotting](https://www.sciencedirect.com/topics/medicine-and-dentistry/western-blot) (n = 5). **J-M** ASC, pro-caspase-1 and pro-IL-1β protein expressions in LPS (4 µg/2 µL) primed rotenone (1.5 mg/kg) induced mice midbrain were measured by [western blotting](https://www.sciencedirect.com/topics/pharmacology-toxicology-and-pharmaceutical-science/western-blot) (n = 5). Data are presented as mean ± SEM. ^*^*p* < 0.05, ^**^ *p* < 0.01, and ^***^ *p* < 0.001.

**Fig. S4**

**
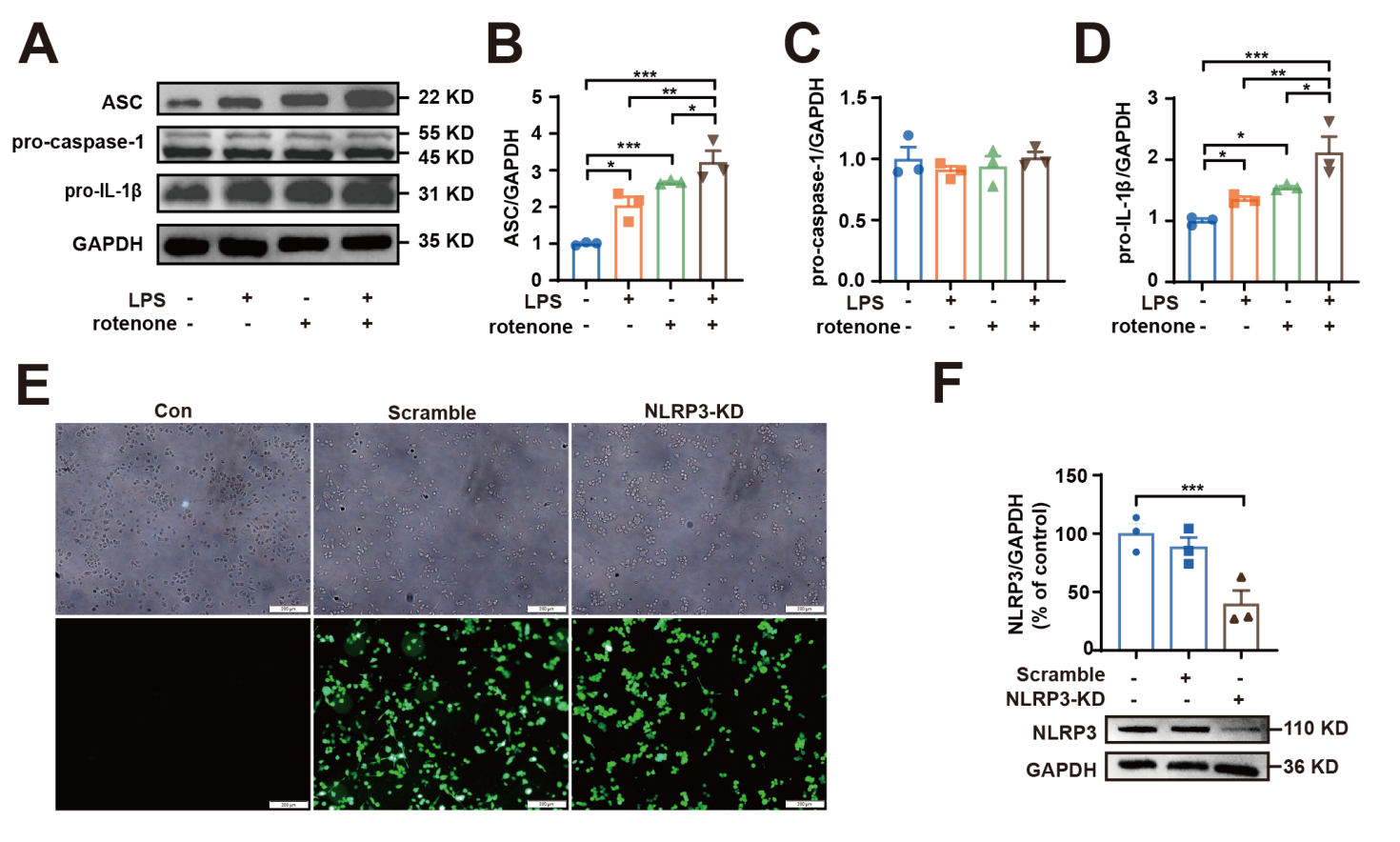
**

**Fig. S4 Effect of LPS and rotenone on the NLRP3 inflammasome pathway and validation of NLRP3 knockdown in BV2 Cells. A-D** ASC, pro-caspase-1 and pro-IL-1β protein expressions in LPS (100 ng/mL) primed rotenone (0.1 μM) induced BV2 cells were measured by [western blotting](https://www.sciencedirect.com/topics/pharmacology-toxicology-and-pharmaceutical-science/western-blot) (n = 3). **E** Representative image to assess the transfection efficiency of LV-NLRP3 in BV2 cells. Scale bar = 200 μm. **F** Western blotting was conducted to evaluate the knockdown efficiency of NLRP3 (n = 3). Data are presented as mean ± SEM. ^*^*p* < 0.05, ^**^ *p* < 0.01, and ^***^ *p* < 0.001.

**Fig. S5**

**
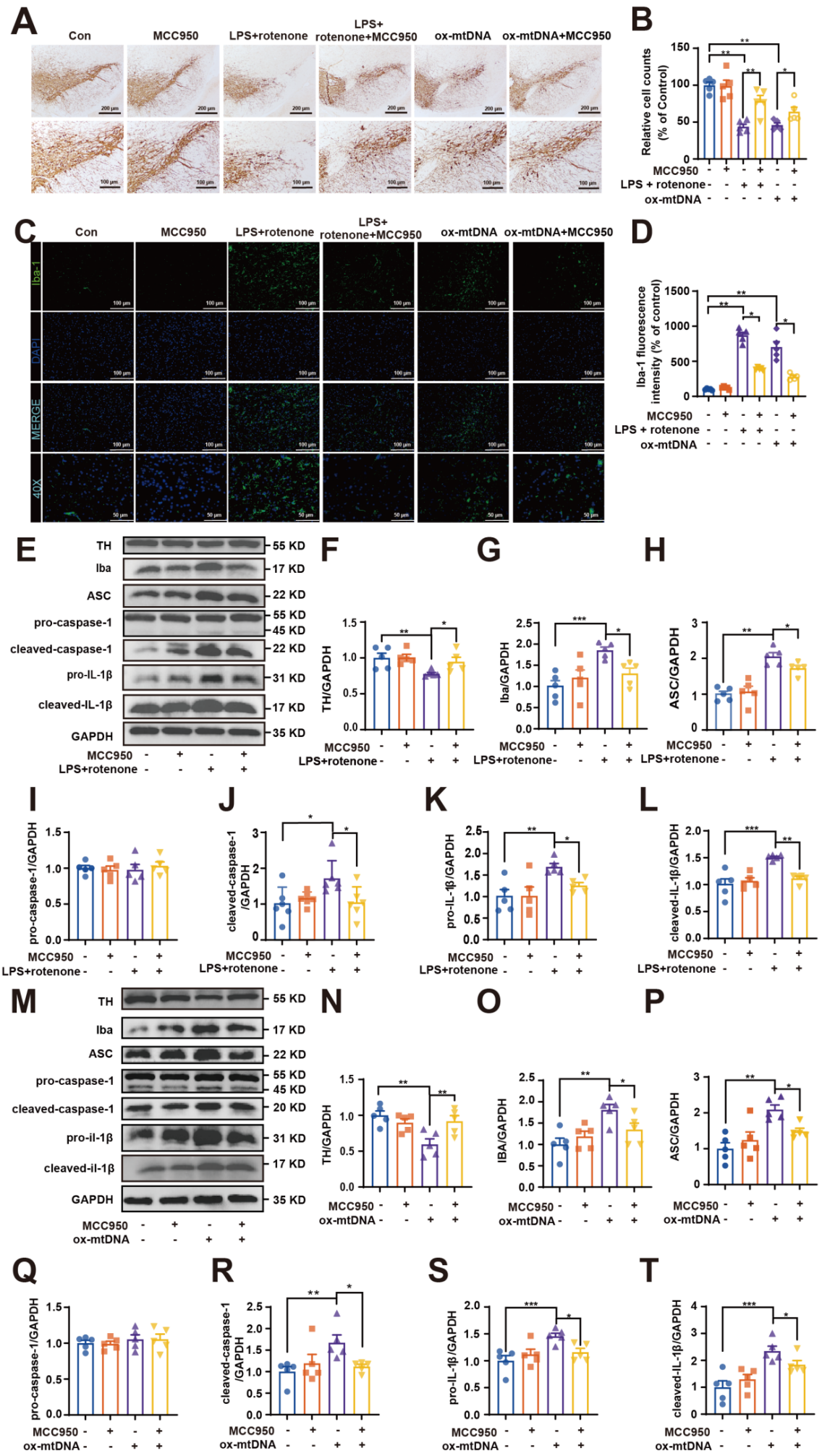
**

**Fig. S5 The NLRP3 inhibitor MCC950 attenuates microglial activation and preserves dopaminergic neurons in LPS primed rotenone or ox-mtDNA induced PD models. A and B** Immunohistochemical [staining](https://www.sciencedirect.com/topics/neuroscience/staining-technique) and quantification of TH-positive neurons in [SN](https://www.sciencedirect.com/topics/medicine-and-dentistry/substantia-nigra) were determined (n = 5). Scale bar = 200 μm (top), 100 μm (bottom). **C and D** Immunofluorescence [staining](https://www.sciencedirect.com/topics/neuroscience/staining-technique) and quantification of the fluorescence intensity of Iba-1 (green) in the midbrain were determined (n = 5). Scale bar = 100 μm (top), 50 μm (bottom). **E-L** TH, Iba, ASC, pro-caspase-1, cleaved-caspase-1, pro-IL-1β and cleaved-IL-1β protein expressions in LPS (4 µg/2 µL) primed rotenone (1.5 mg/kg) induced mice midbrain treated with MCC950 (10 mg/kg) were determined by Western blotting (n = 5). **M-T** TH, Iba, ASC, pro-caspase-1, cleaved-caspase-1, pro-IL-1β and cleaved-IL-1β protein expressions in LPS (4 µg/2 µL) primed ox-mtDNA (2 µg/2 µL) induced mice midbrain treated with MCC950 (10 mg/kg) were determined by Western blotting (n = 5). Data are presented as mean ± SEM. ^*^*p* < 0.05, ^**^ *p* < 0.01, and ^***^ *p* < 0.001.

**Fig. S6**


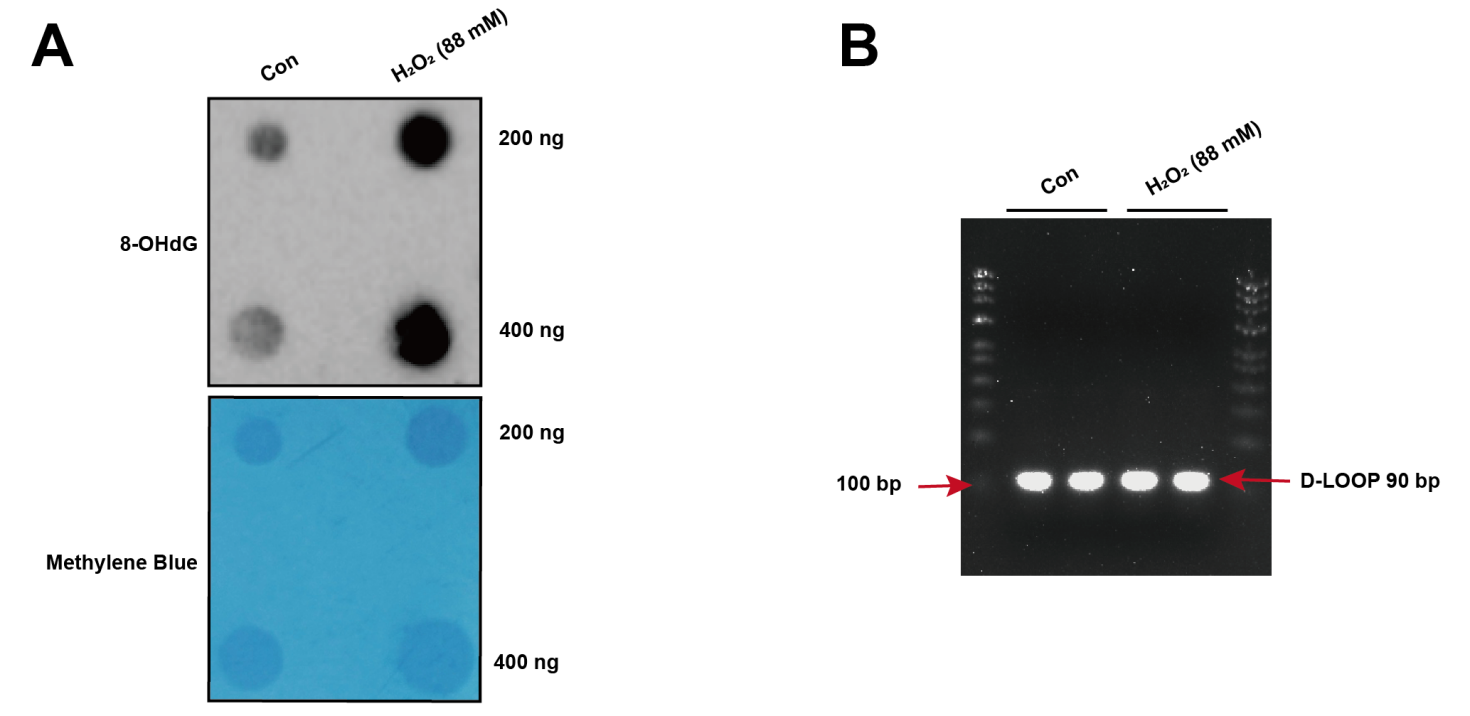


**Fig. S6 Characterization of synthesized oxidized mtDNA: 8-OHdG Modification Assessment and Integrity Verification. A** 8-OHdG detection of synthesized 90 bp mtDNA oxidized by hydrogen peroxide (88 mM). **B** Agarose gel electrophoresis validated that the synthesised 90 bp mtDNA is not degraded by incubating with hydrogen peroxide.

**Fig. S7**


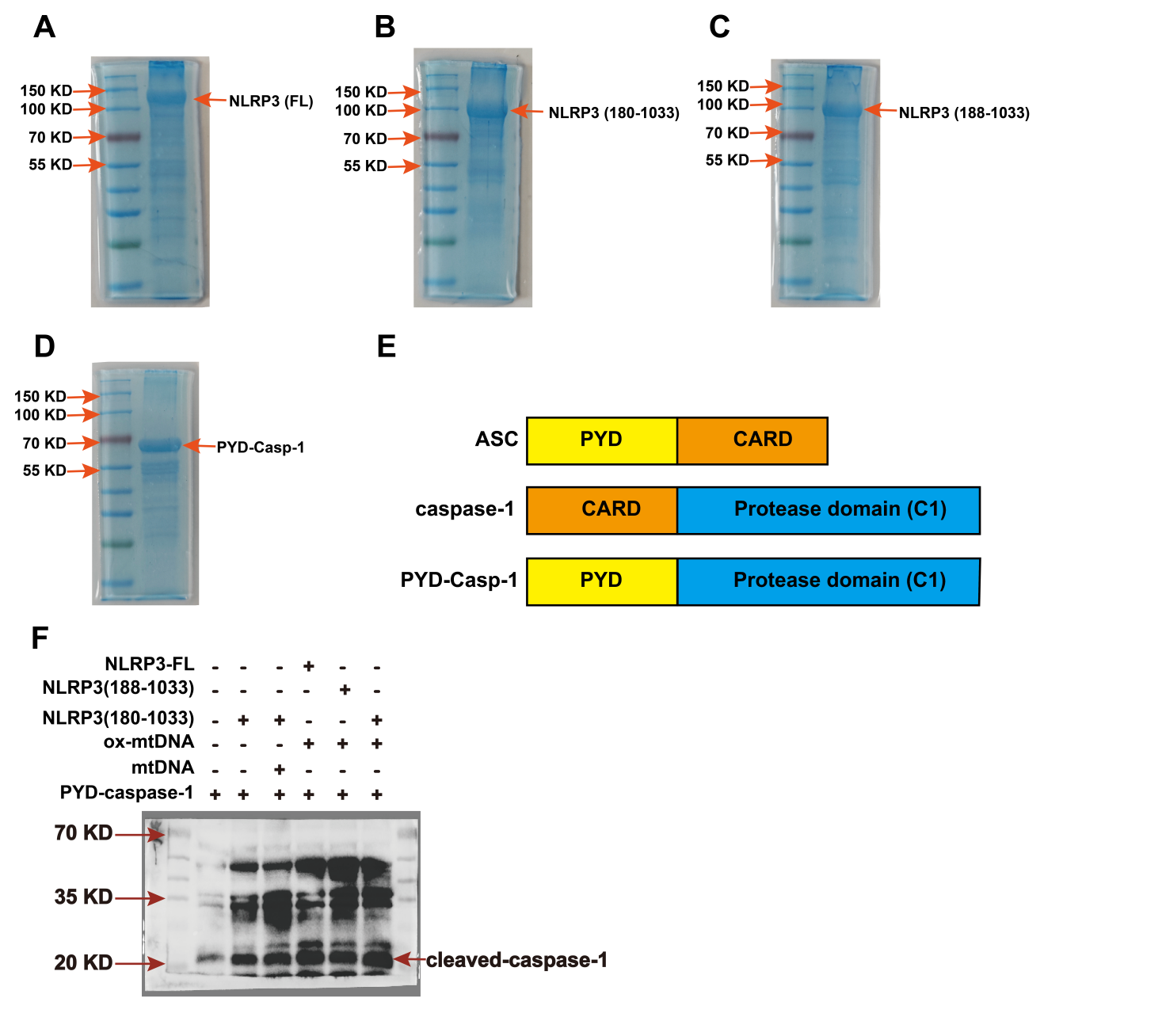


**Fig. S7 The purity of NLRP3 and its truncated proteins and in vitro PYD-caspase-1 cleavage assays. A-D** NLRP3 (FL), NLRP3 (180-1033), NLRP3 (188-1033) and PYD-Caspase-1 protein by Western blotting. **E** Design of PYD-caspase-1 protein. **F** Immunoblot for cleaved-caspase-1 following in vitro incubation of PYD-caspase-1 with purified protein NLRP3 (FL), NLRP3 (180-1033), NLRP3 (188-1033) with oxidized or non-oxidized mtDNA.

**Fig. S8**


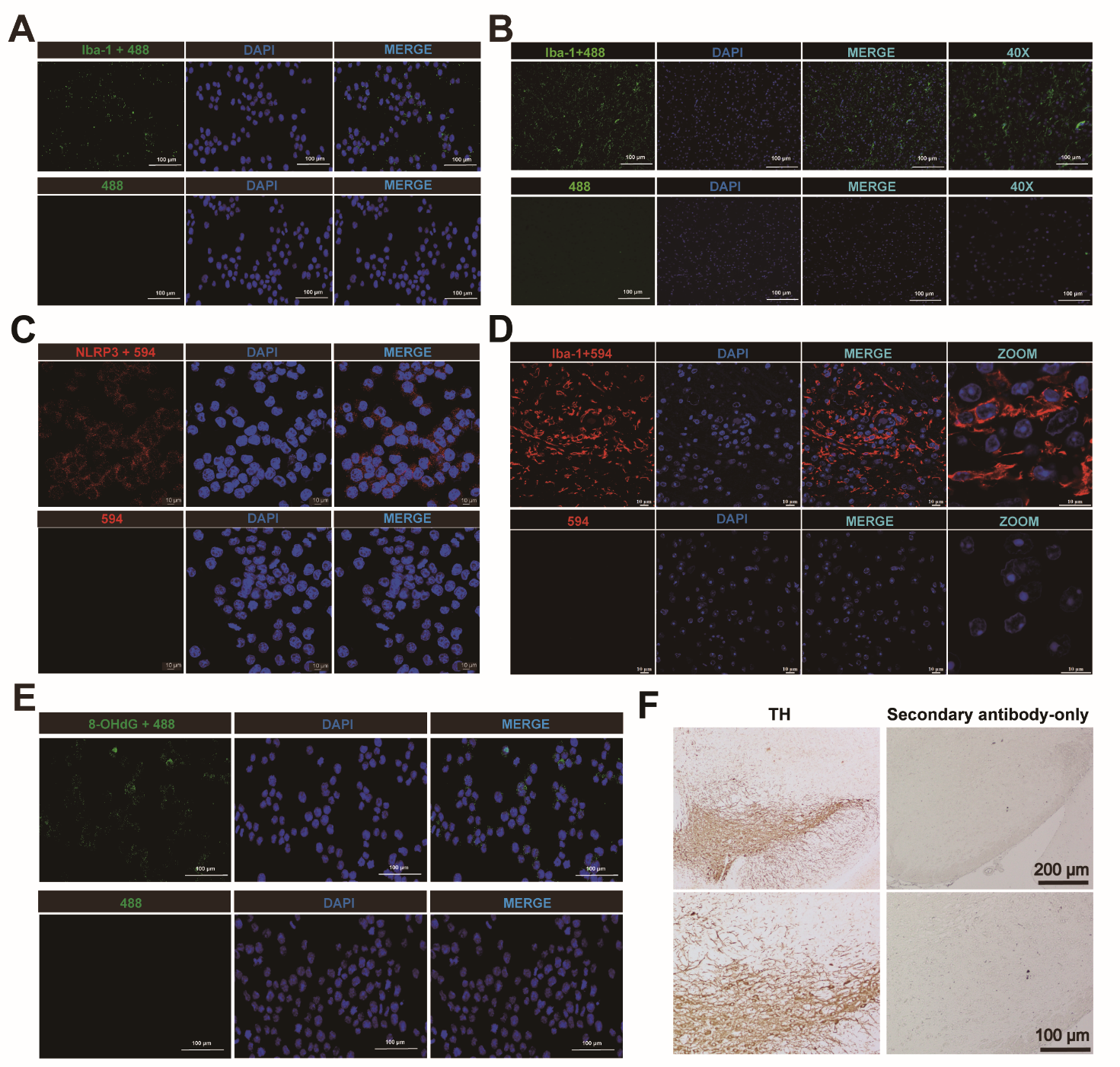


**Fig. S8 Validation of antibody specificity for ICC and IHC.** **A** Immunofluorescence staining of BV2 cells shows that anti-Iba-1 antibody produces a specific signal (green) in the positive control and no signal in the negative control where the primary antibody was omitted. Scale bar = 100 μm. **B** Immunofluorescence staining of brain paraffin section shows that anti-Iba-1 antibody produces a specific signal (green) in the positive control and no signal in the negative control omitting the primary antibody. Scale bar = 100 μm. **C** Immunofluorescence staining of brain paraffin section shows that anti-NLRP3 antibody produces a specific signal (red) in the positive control and no signal in the negative control omitting primary antibody. Scale bar = 10 μm. **D** Immunofluorescence staining of brain paraffin section shows that anti-Iba-1 antibody produces a specific signal (red) in the positive control and no signal in the negative control omitting the primary antibody. Scale bar = 10 μm. **E** Immunofluorescence staining of brain paraffin section shows that anti-8-OHdG antibody produces a specific signal (green) in the positive control and no signal in the negative control omitting the primary antibody, confirming the specificity of the antibody response. Scale bar = 100 μm. **F** Immunohistochemical staining of brain paraffin section shows that anti-TH antibody produces a specific signal in the positive control and no signal in the negative control omitting primary antibody. Scale bar = 200 μm (top), 100 μm (bottom).
