# Supplementary material for: Mitochondrial DNA drives NLRP3-IL-1β axis activation in microglia by binding to NLRP3, leading to neurodegeneration in Parkinson’s disease models
Source: Cell Death Dis. 2026 Feb 10;17(1):213. doi: 10.1038/s41419-026-08424-7 (PMC12921273; doi:10.1038/s41419-026-08424-7)

Fig. 1H TH


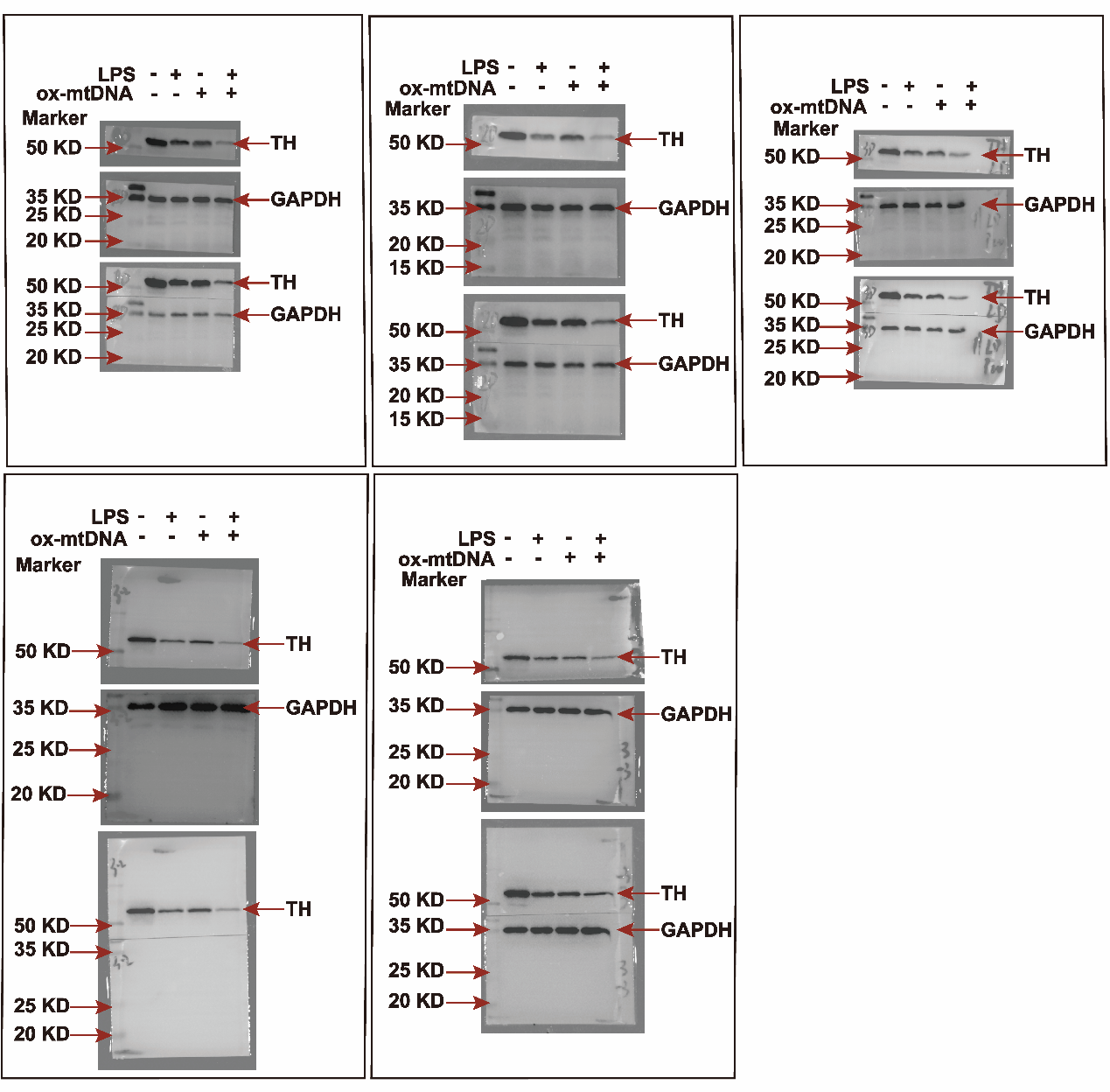


Fig. 2C Iba-1


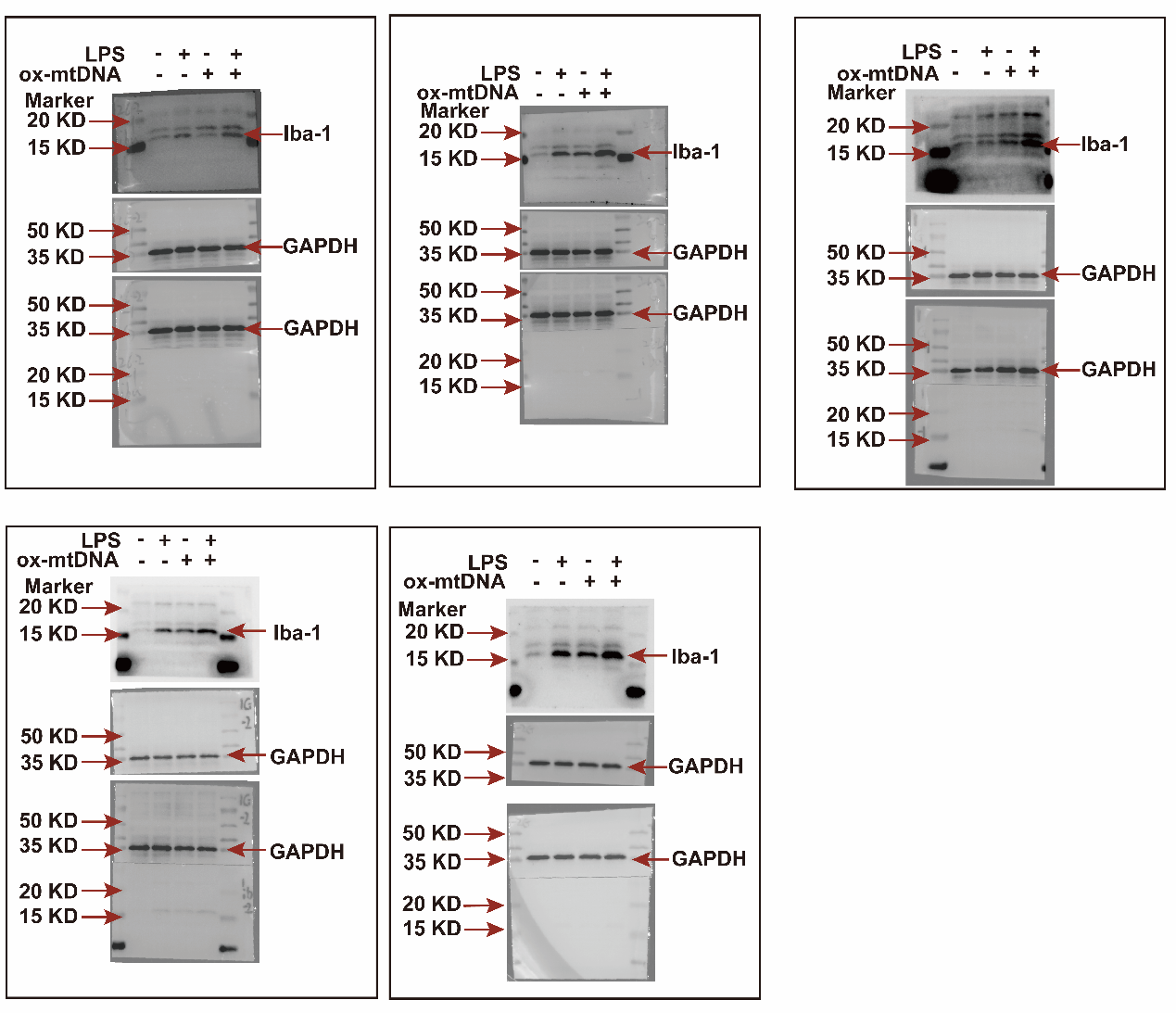


Fig. 2I cleaved-caspase-1


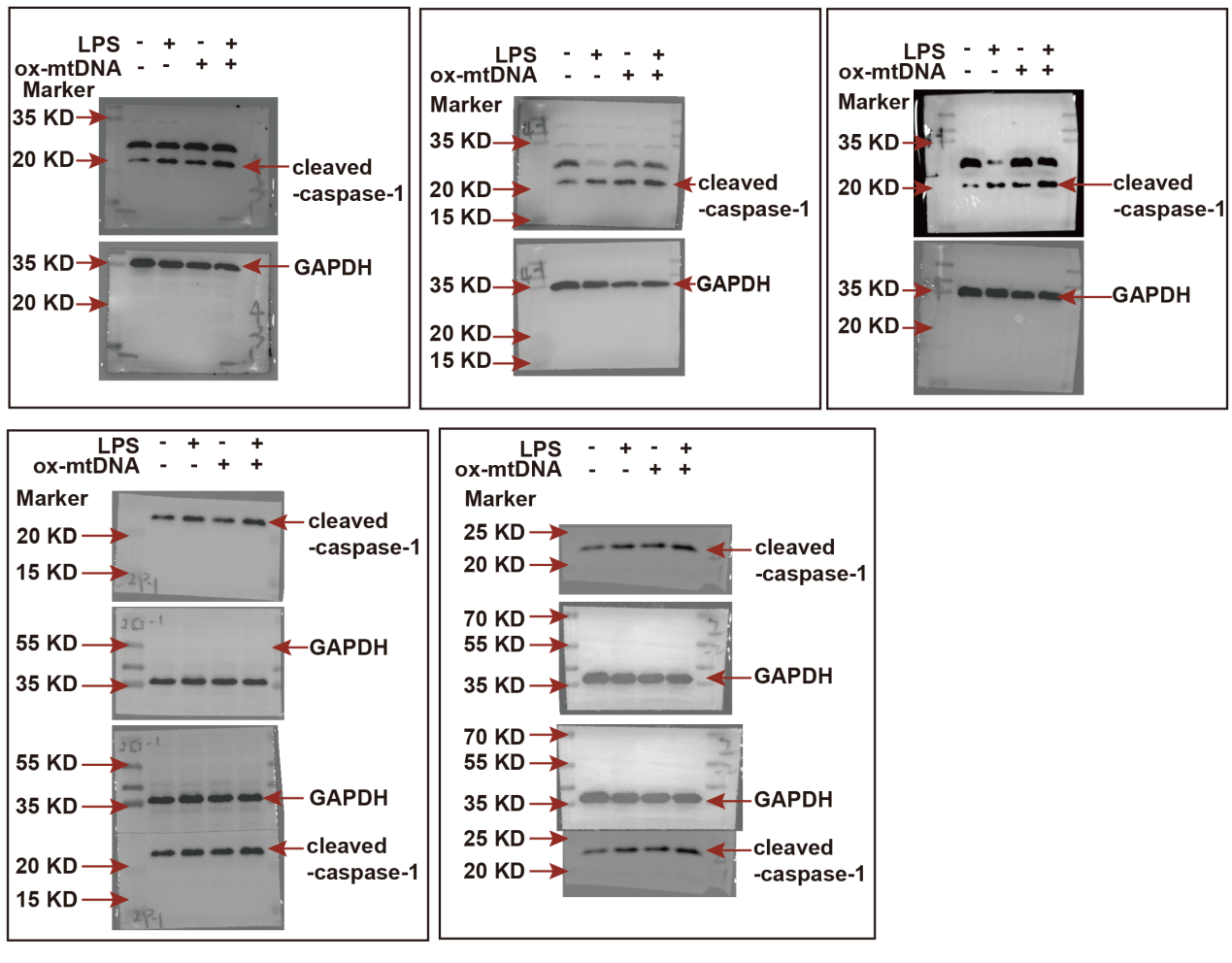


Fig. 2I cleaved-IL-1β


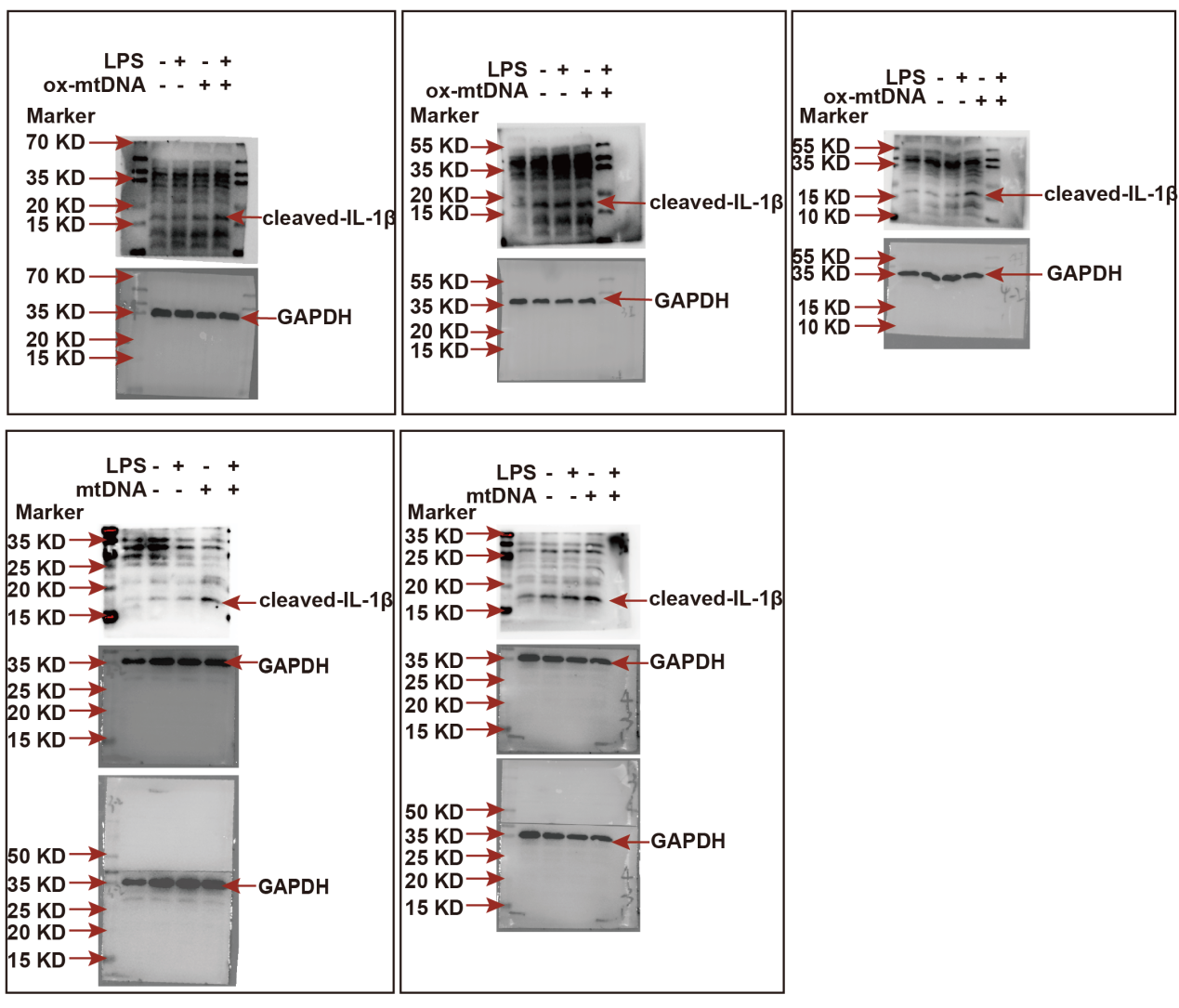


Fig. 3I cleaved-caspase-1


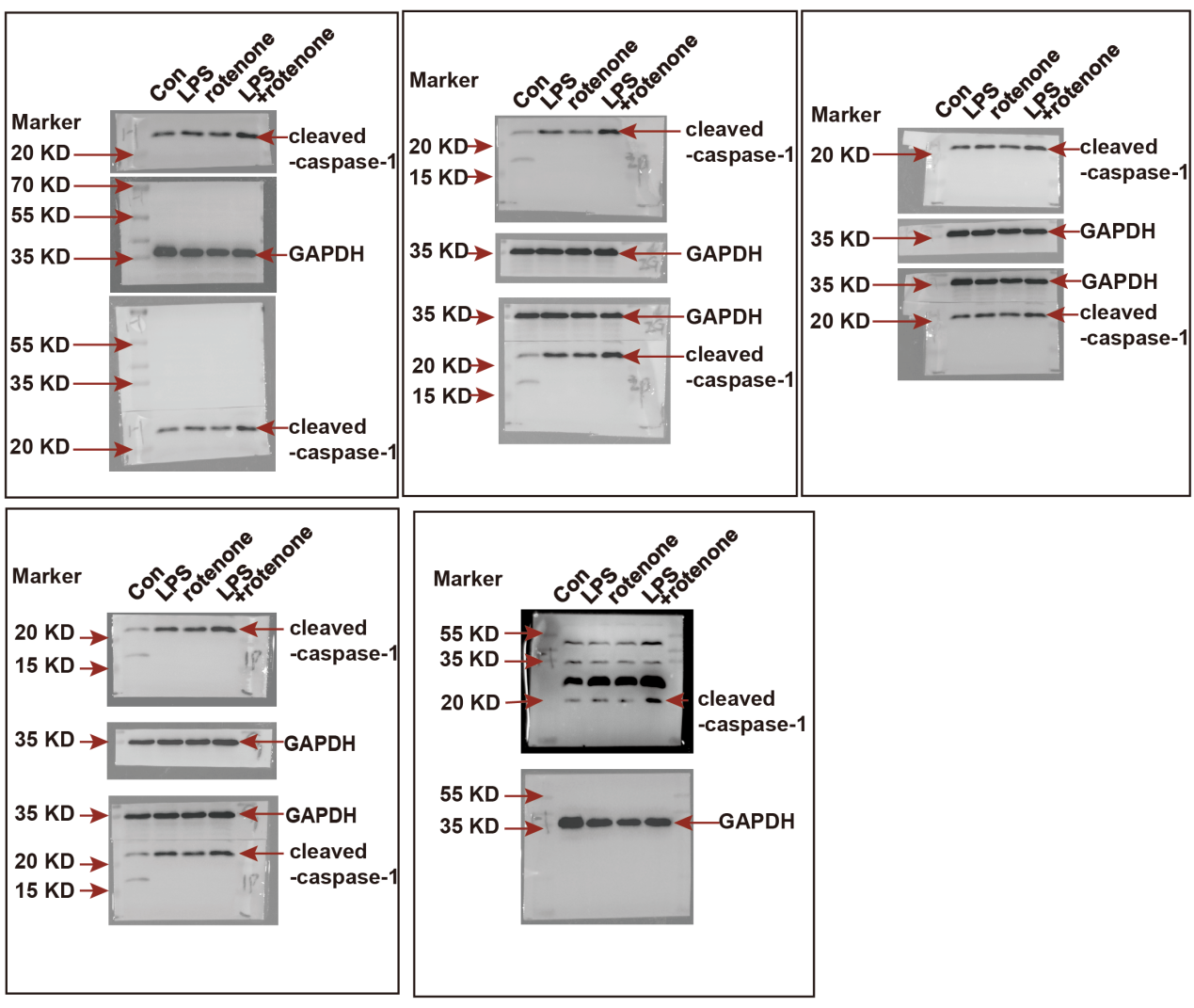


Fig. 3I cleaved-IL-1β


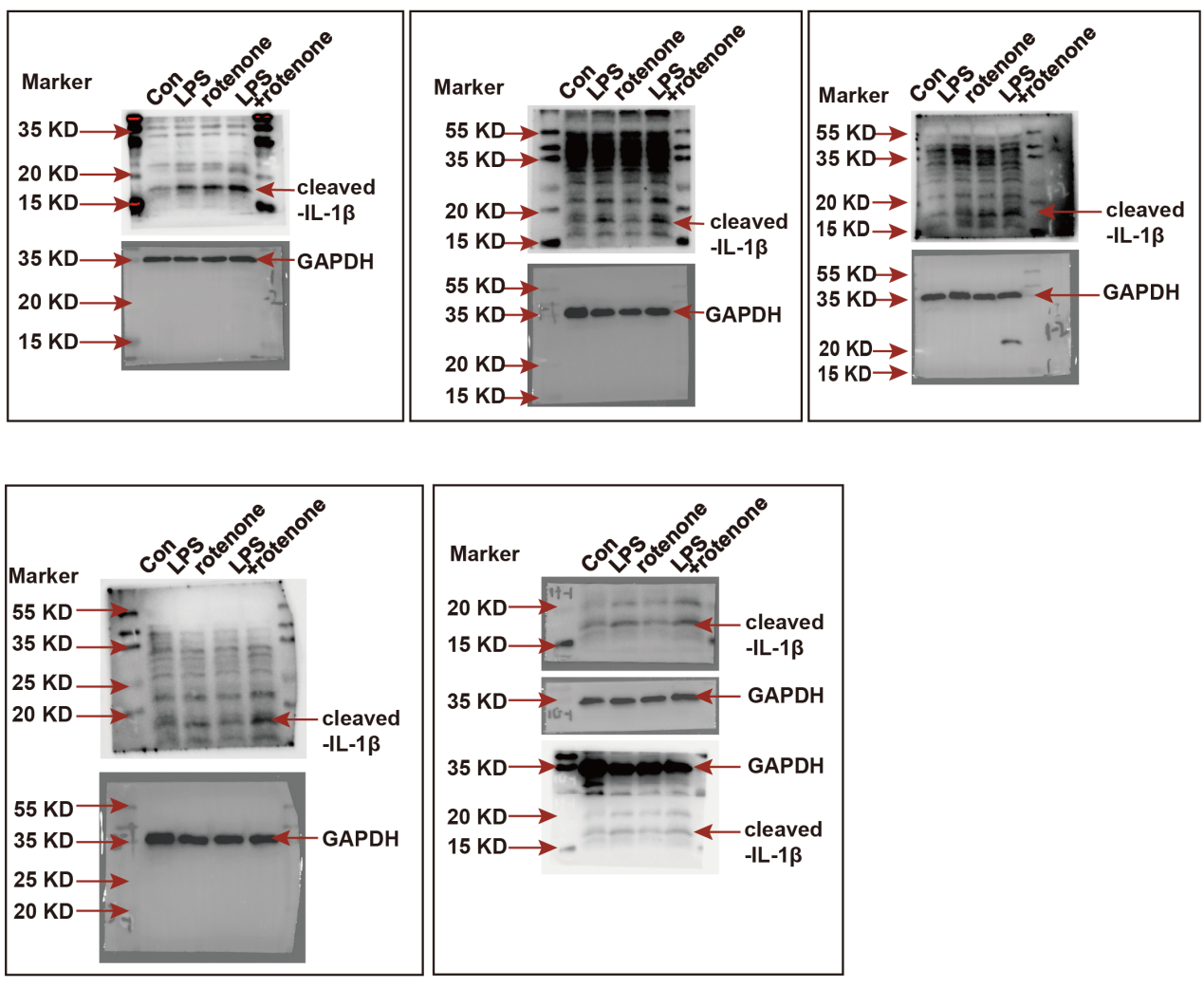


Fig. 4E Lamin B, TFAM, α-Tubulin


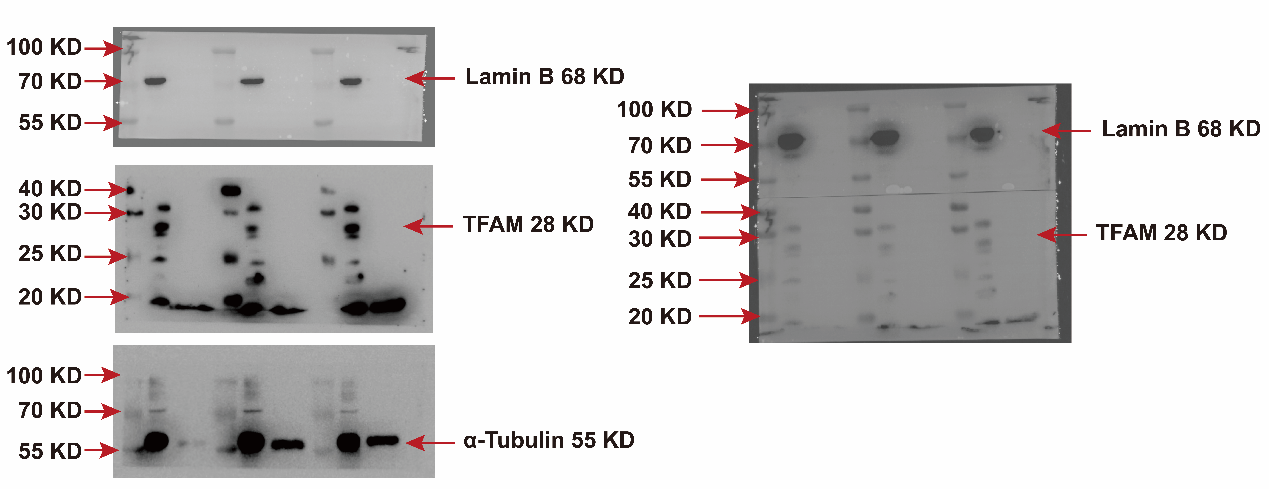


Fig. 5E NLRP3


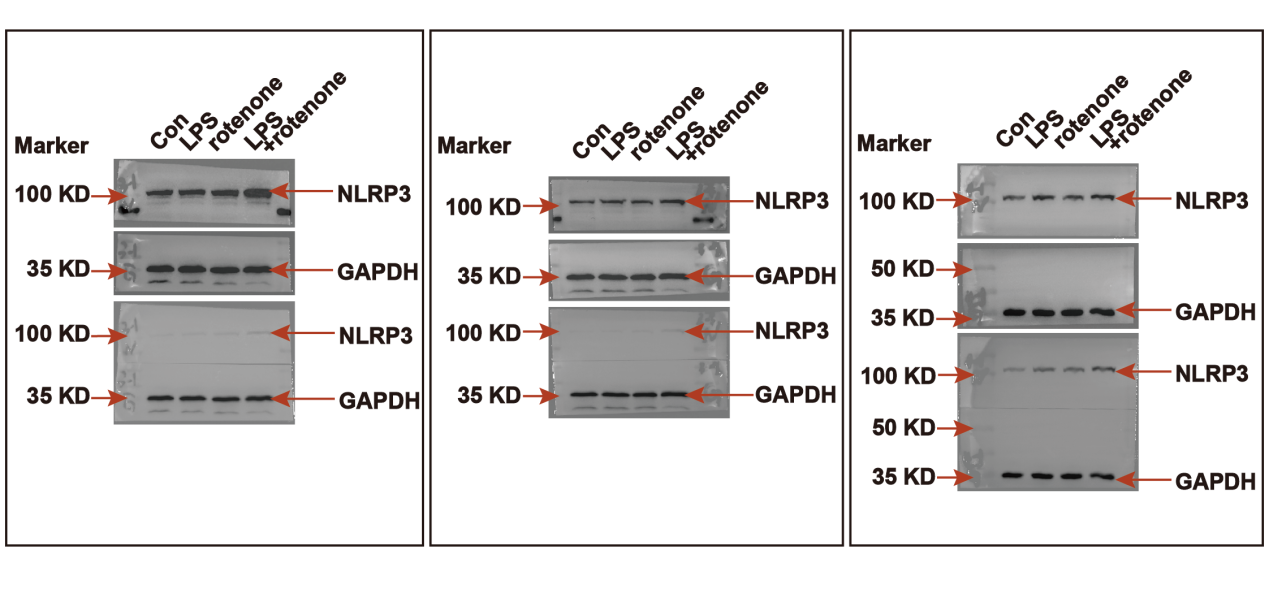


Fig. 5E cleaved-caspase-1


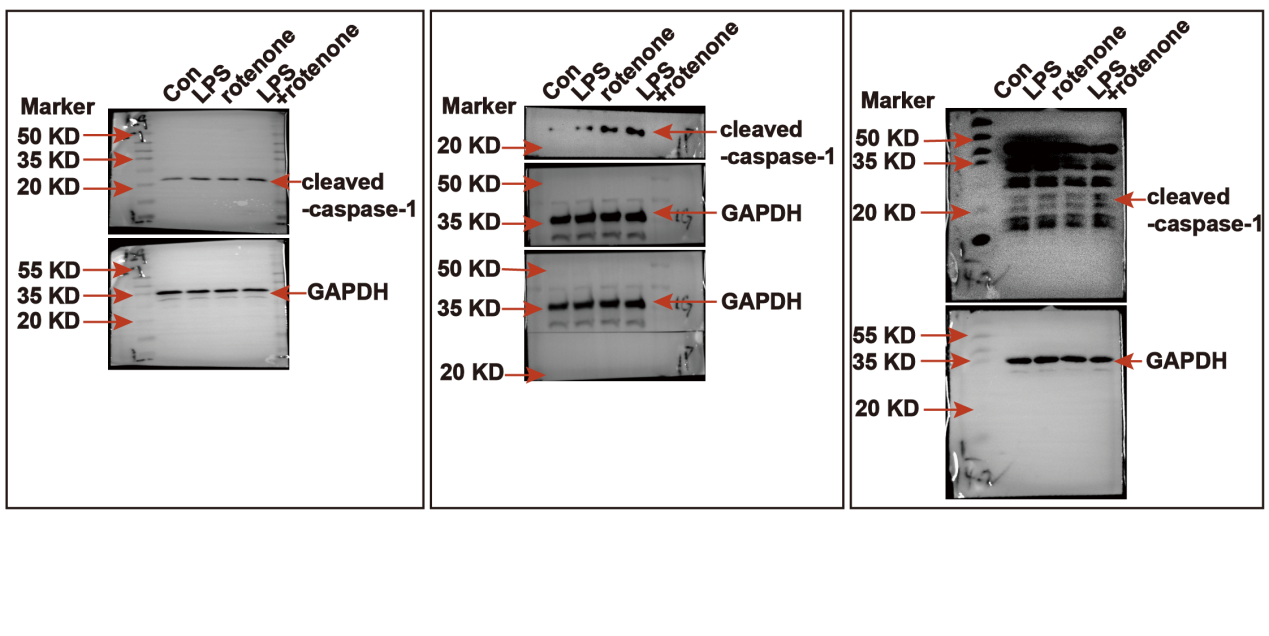


Fig. 5E cleaved-IL-1β


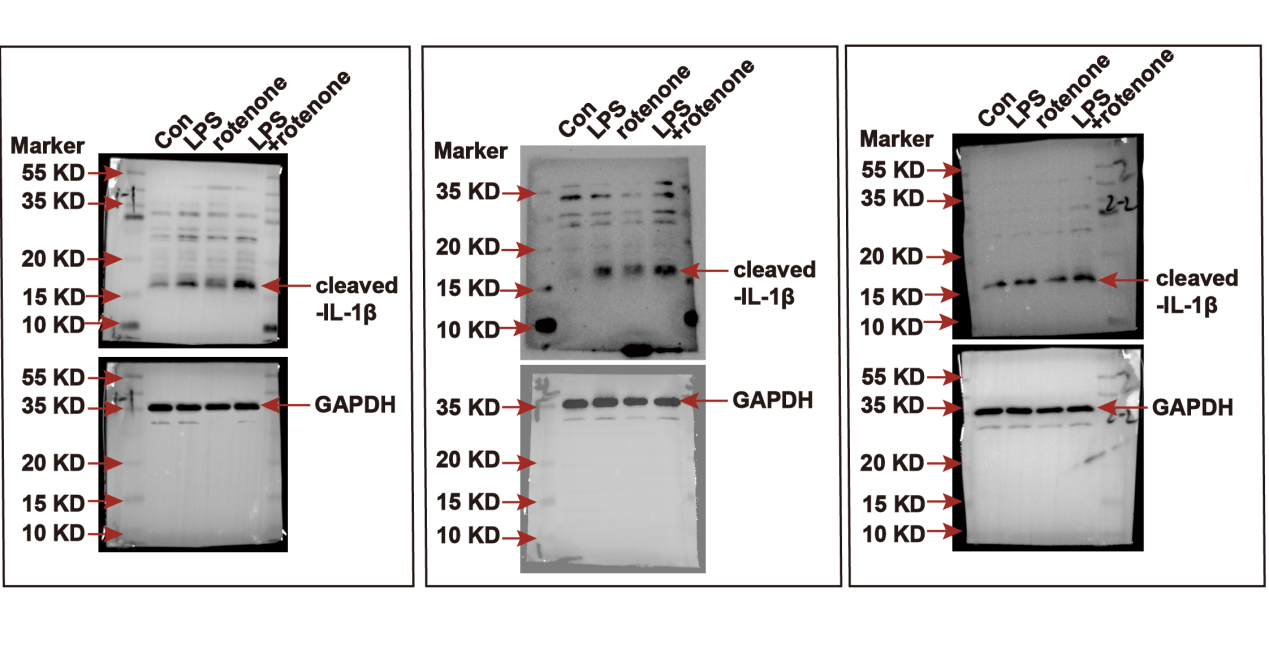


Fig. 5K TH


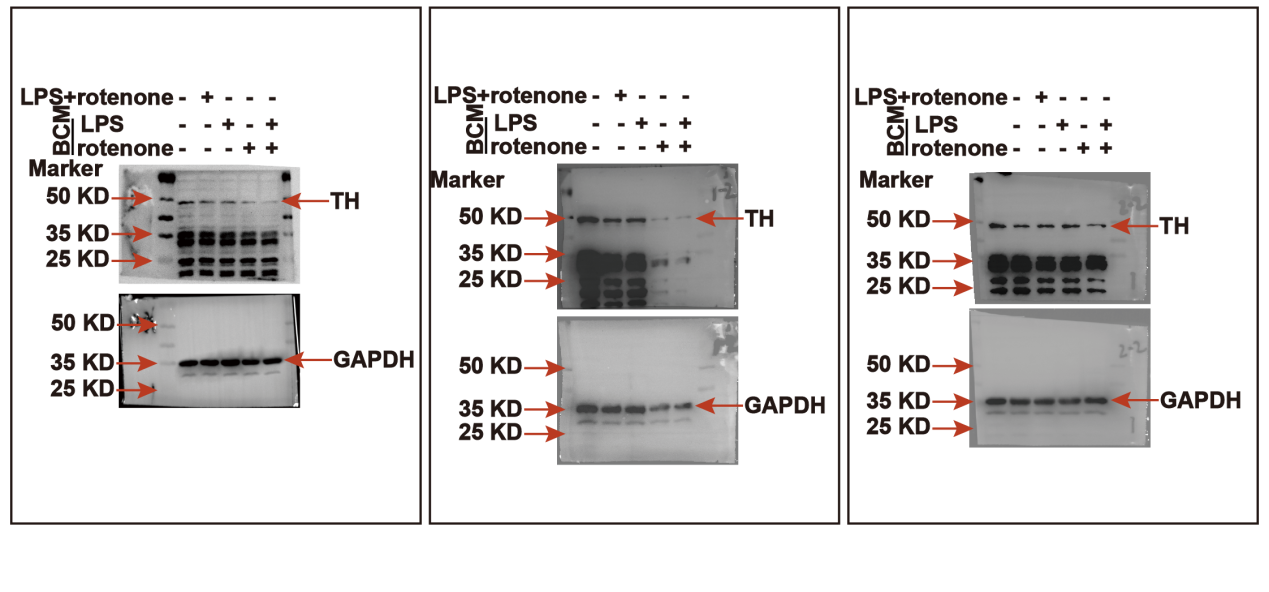


Fig. 5L NLRP3


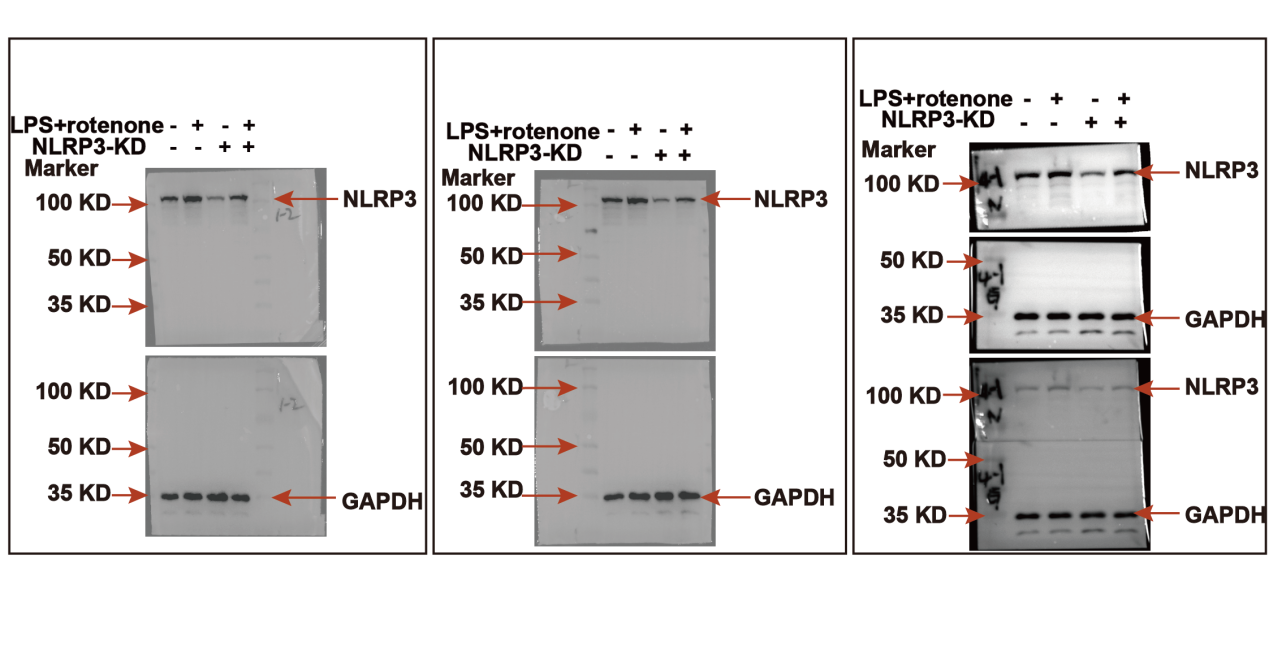


Fig. 5L cleaved-caspase-1


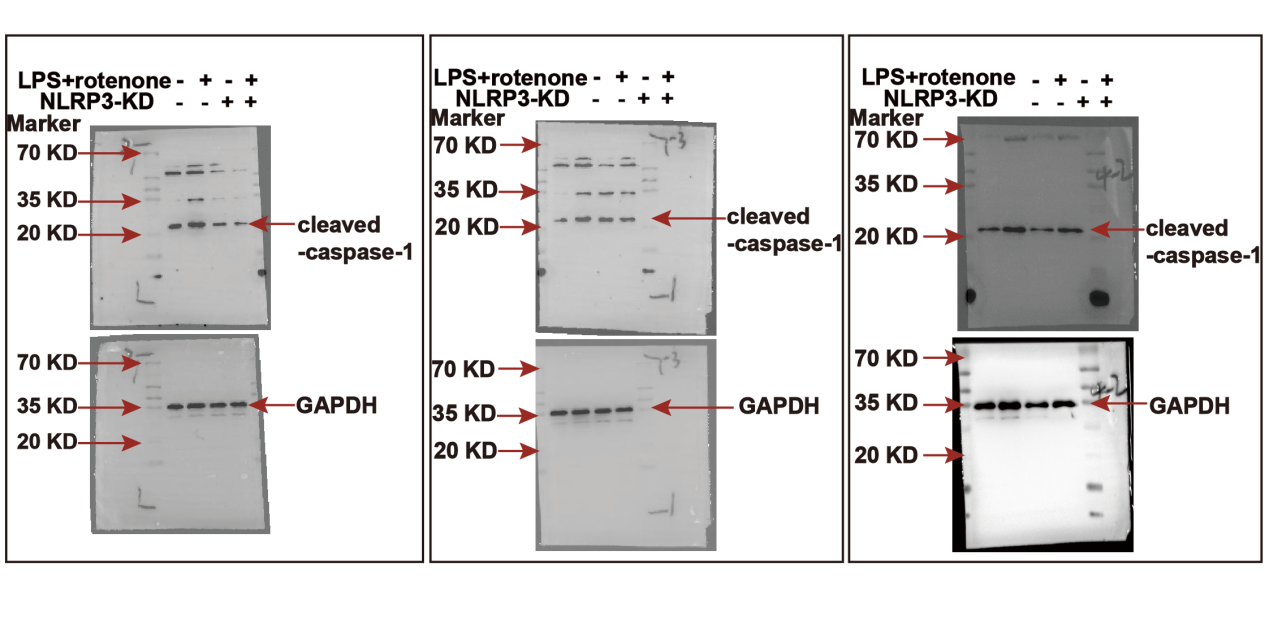


Fig. 5L cleaved-IL-1β


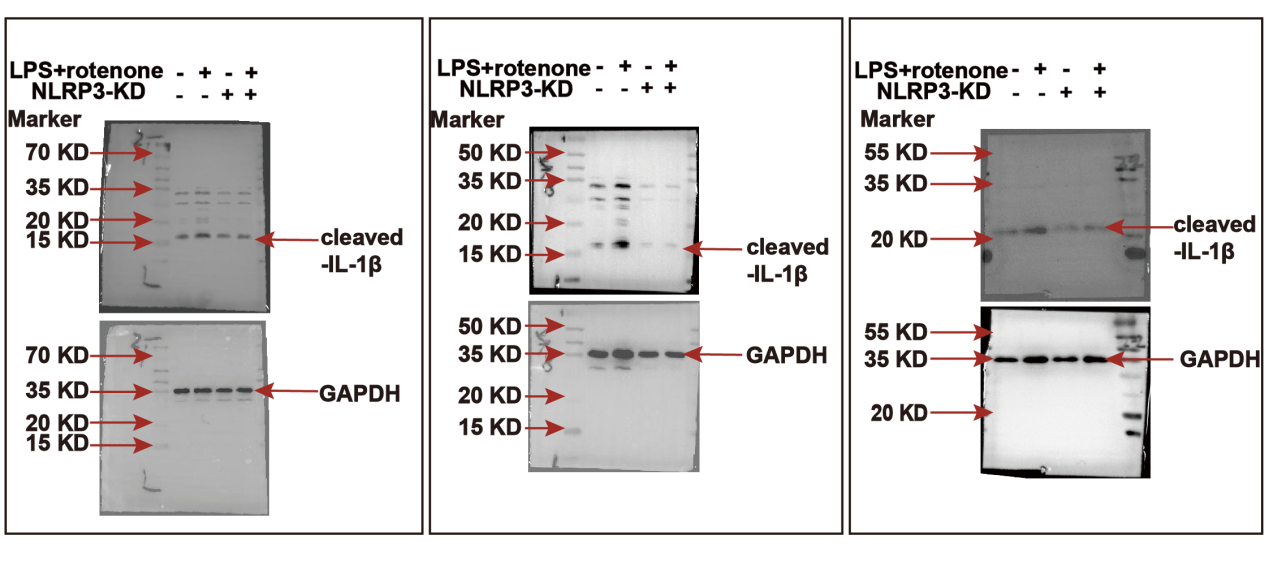


Fig. 5R TH


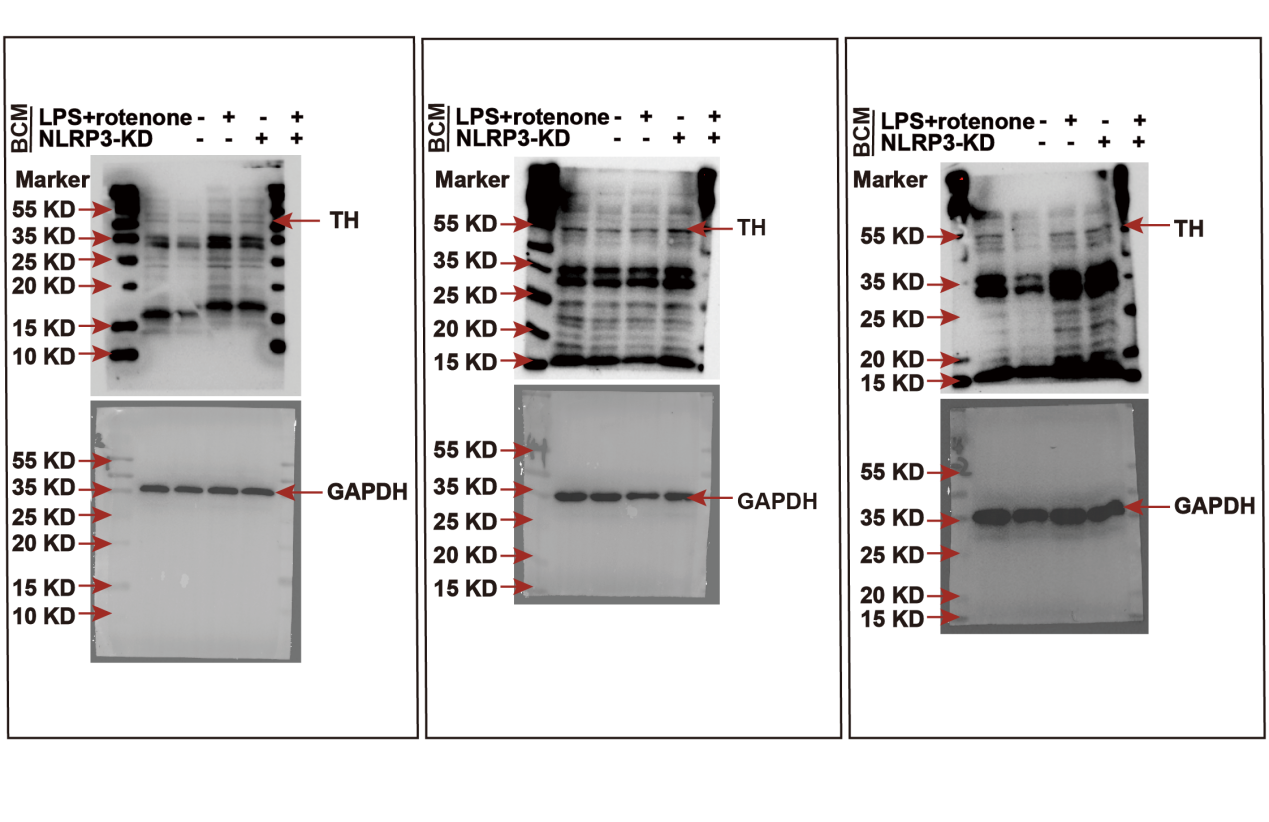


Fig. 6B cleaved-caspase-1


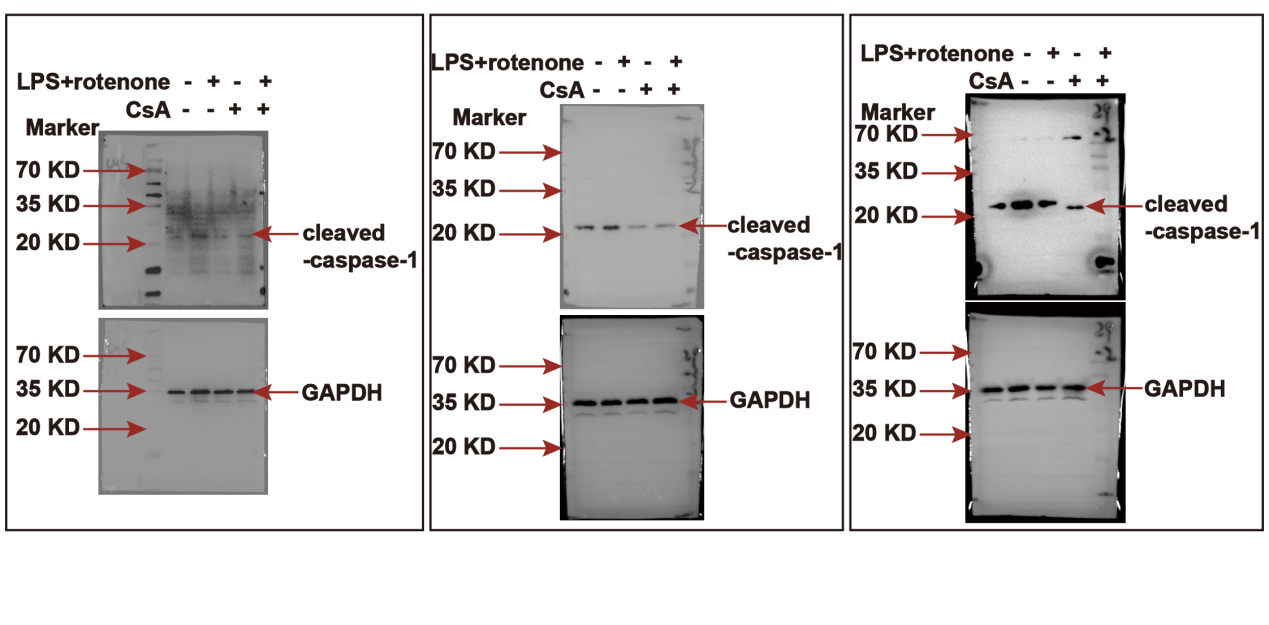


Fig. 6B cleaved-IL-1β


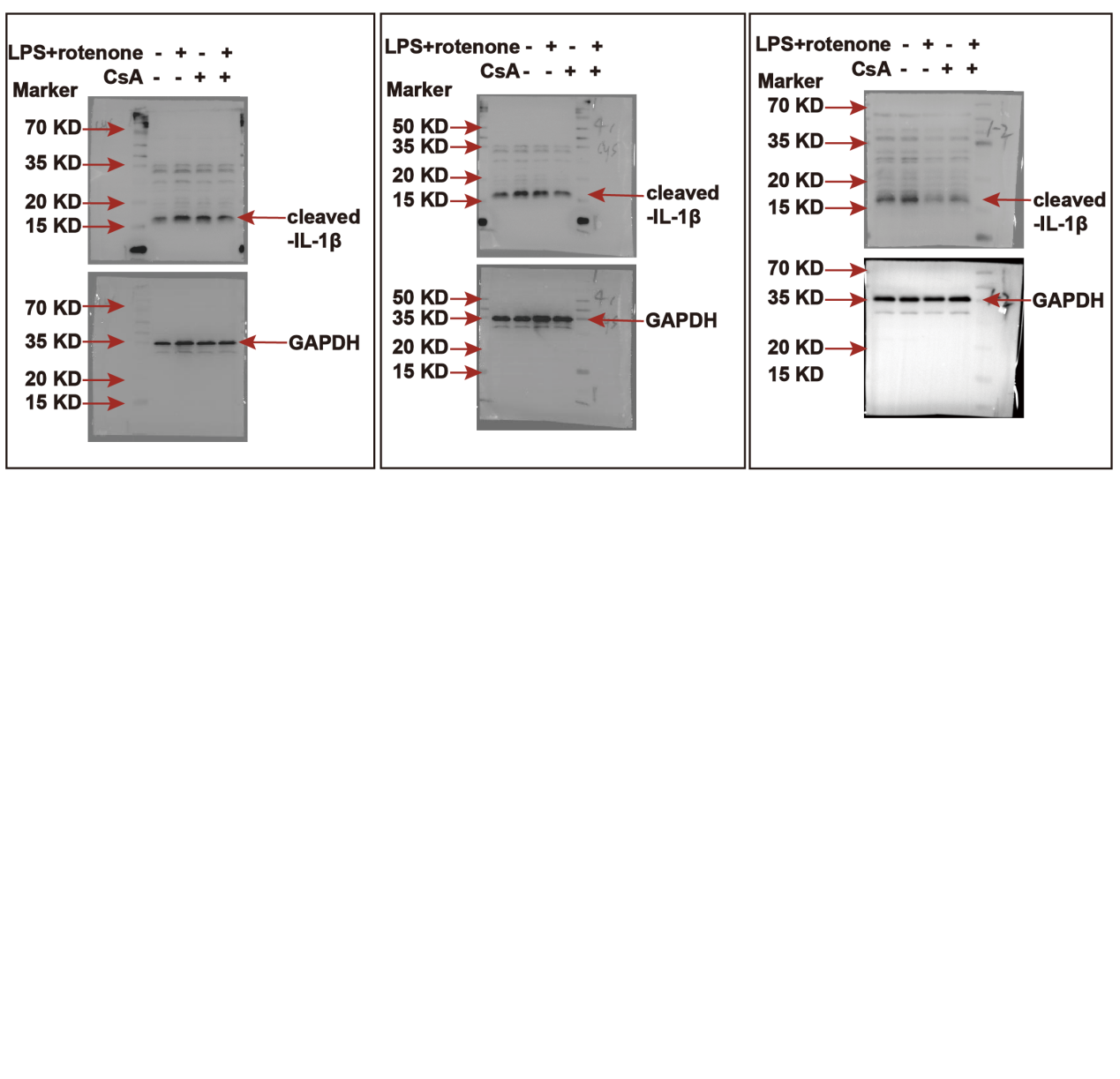


Fig. 6E cleaved-caspase-1


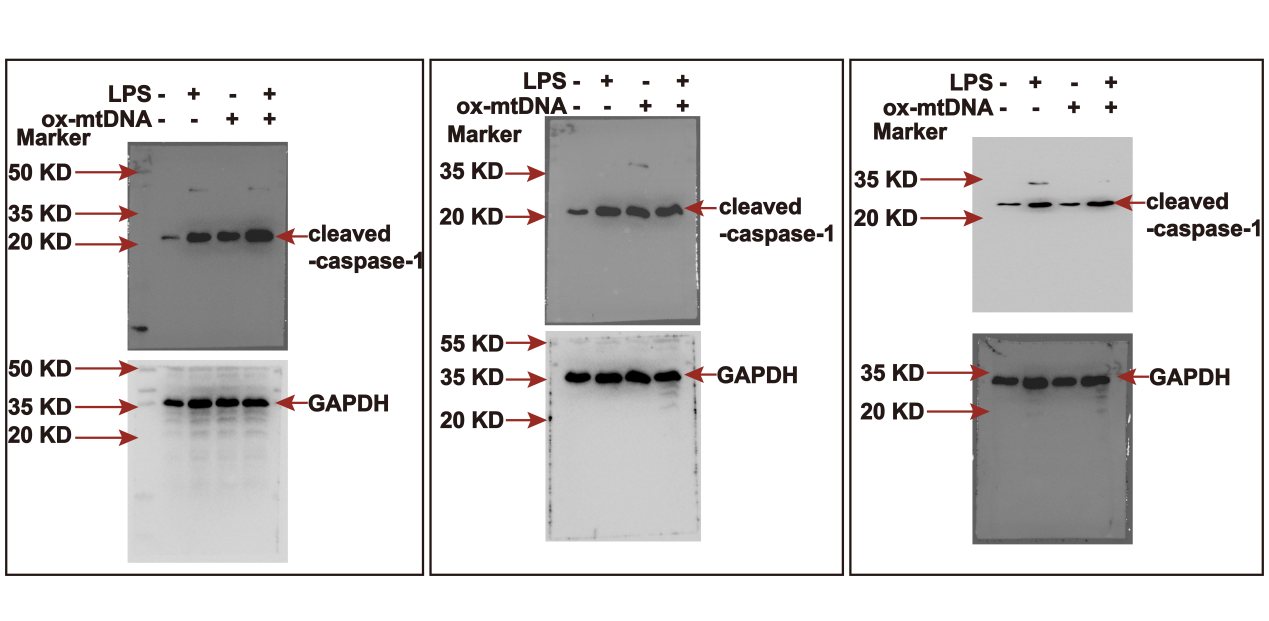


Fig. 6E cleaved-IL-1β


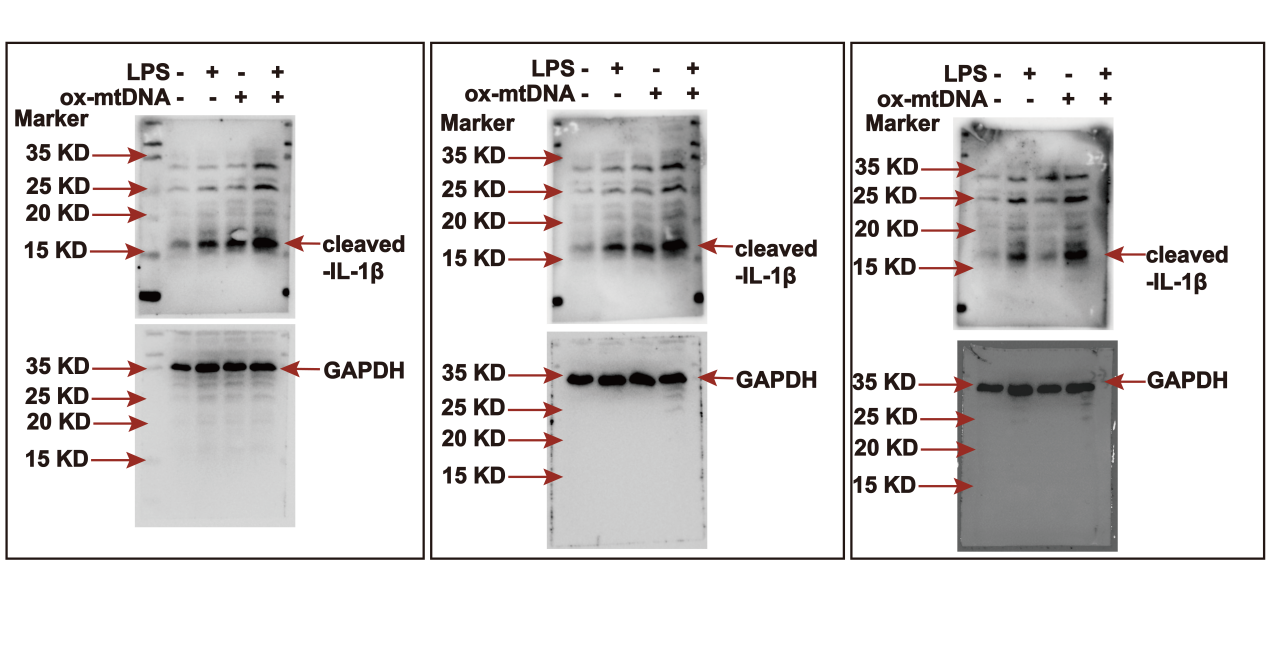


Fig. 6H cleaved-caspase-1


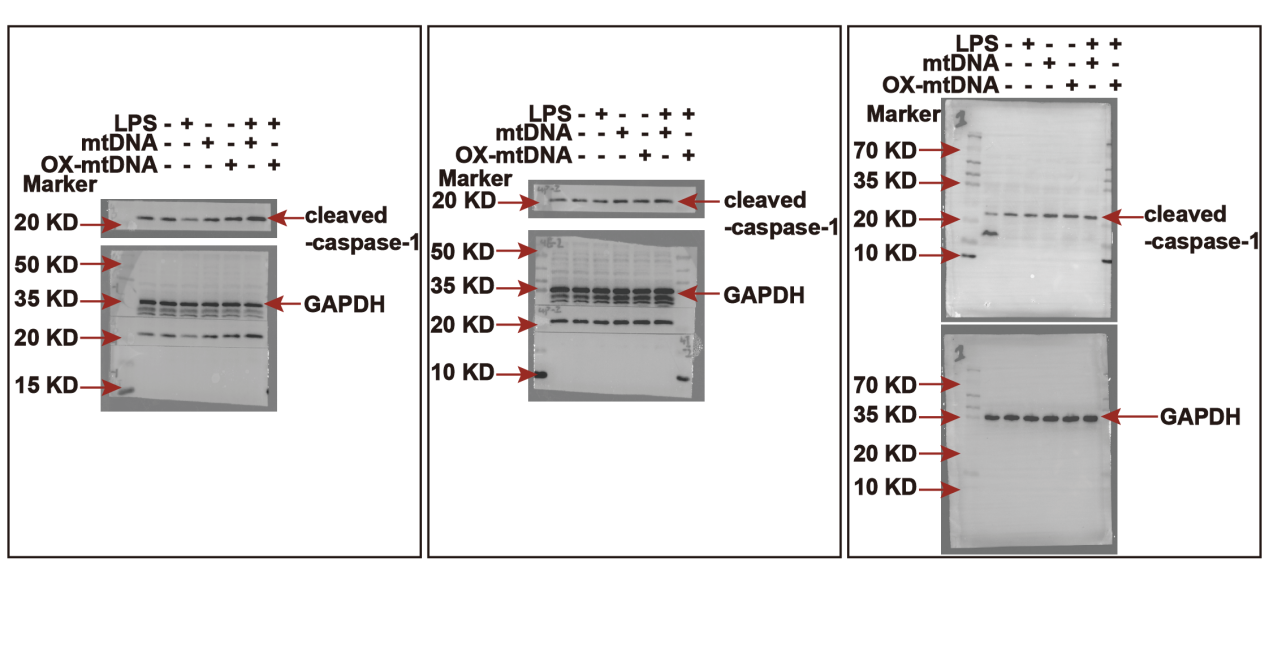


Fig. 6H cleaved-IL-1β


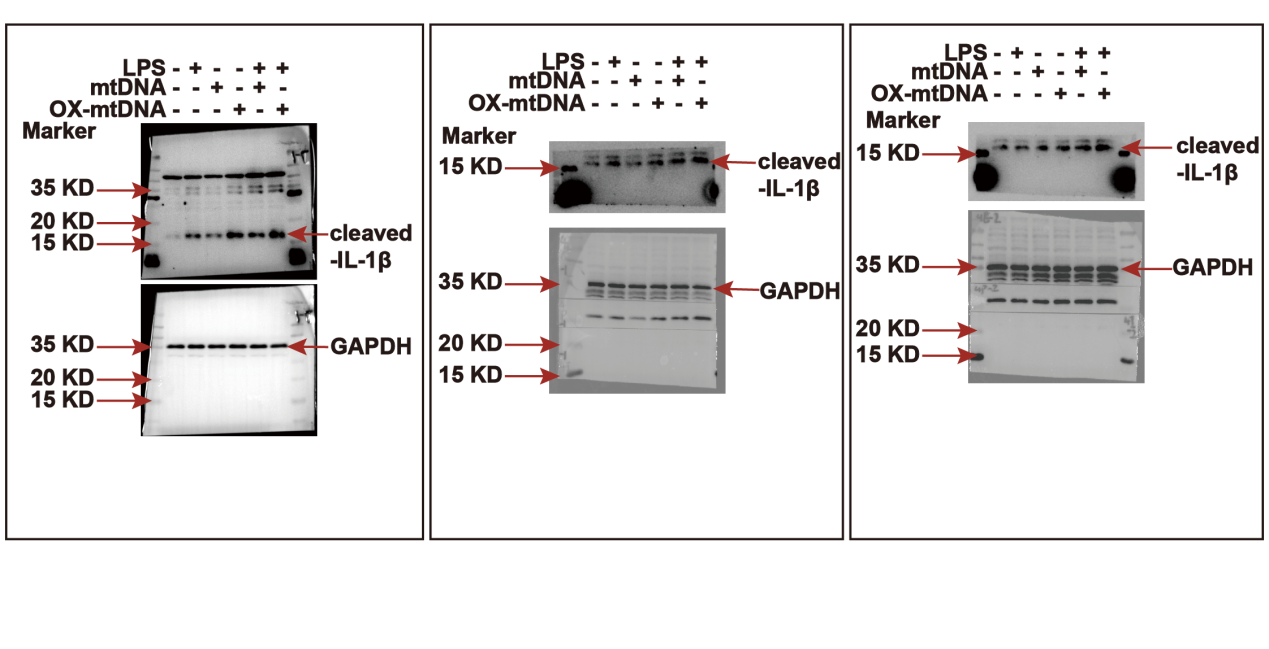


Fig. 7H cleaved-caspase-1


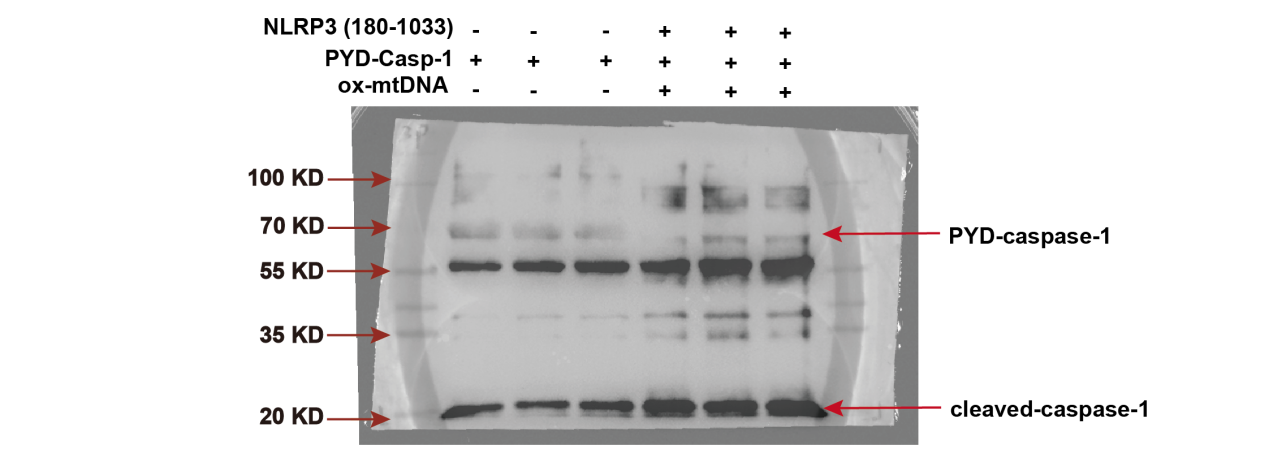


Fig. S2A ASC


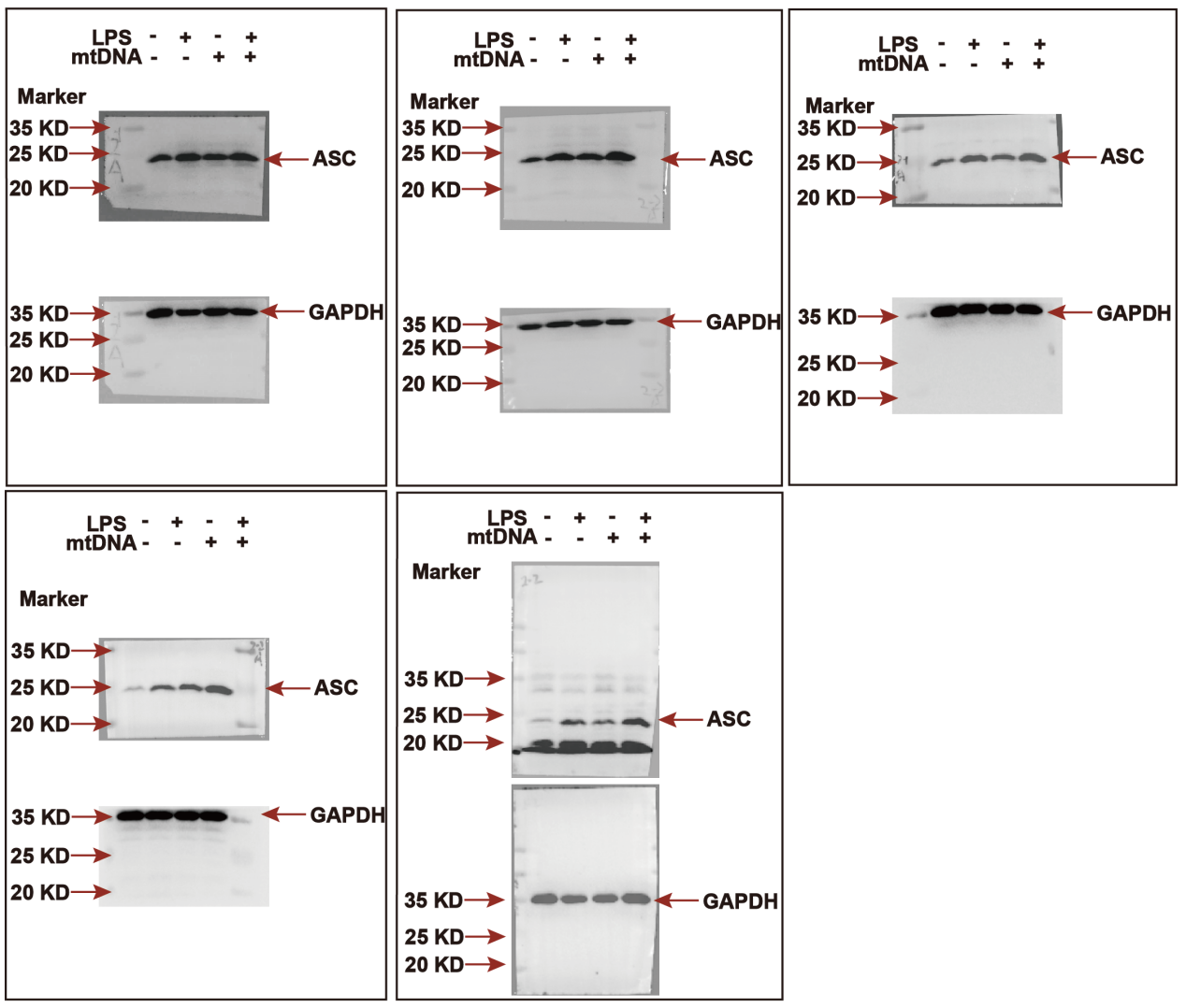


Fig. S2A pro-caspase-1


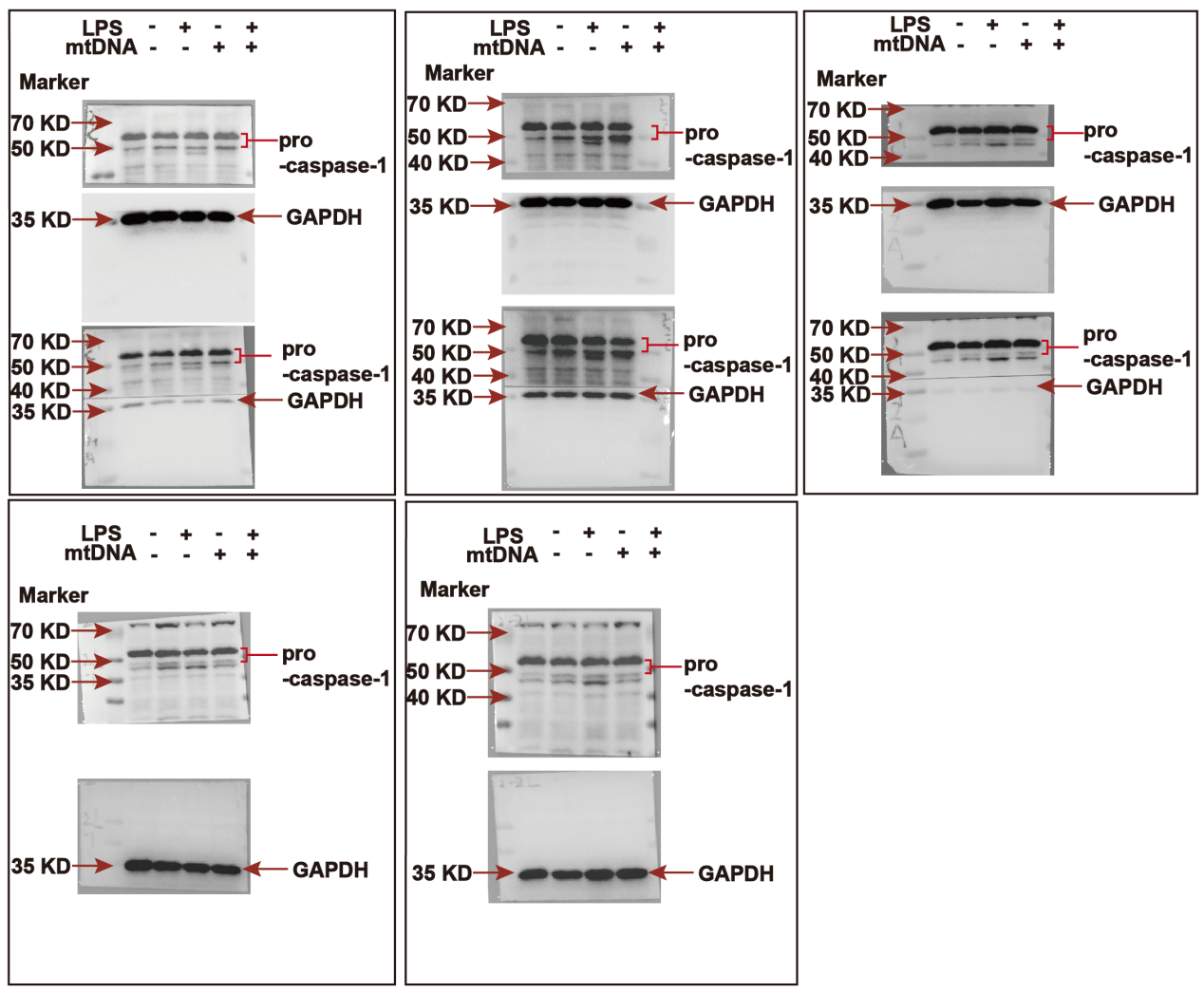


Fig. S2A pro-IL-1β


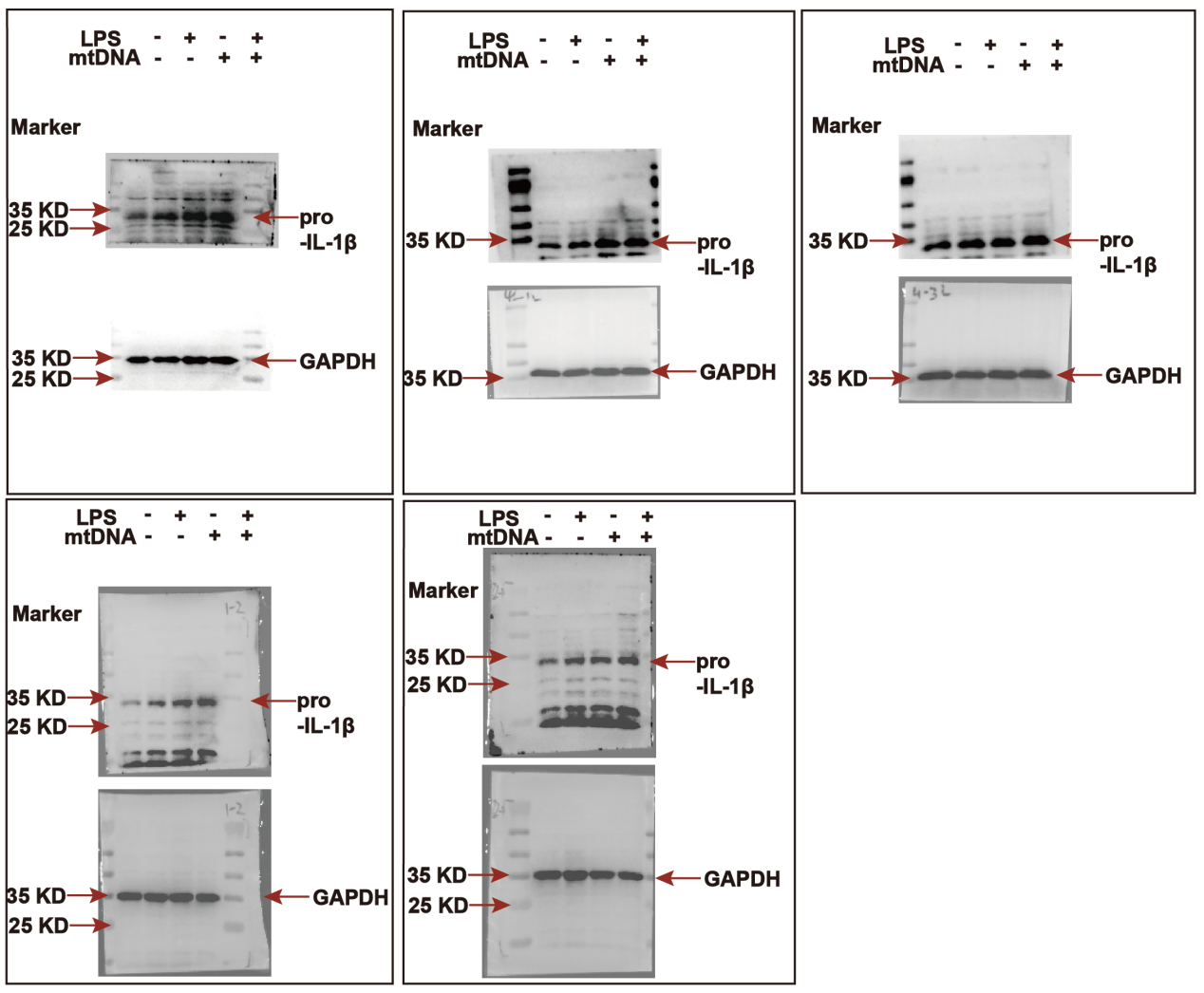


Fig. S3C Iba-1


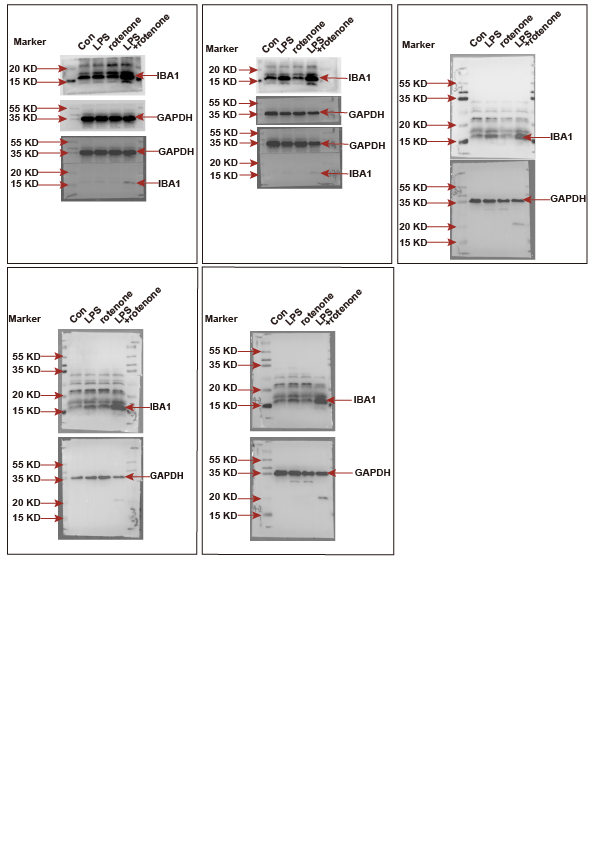


Fig. S3I TH


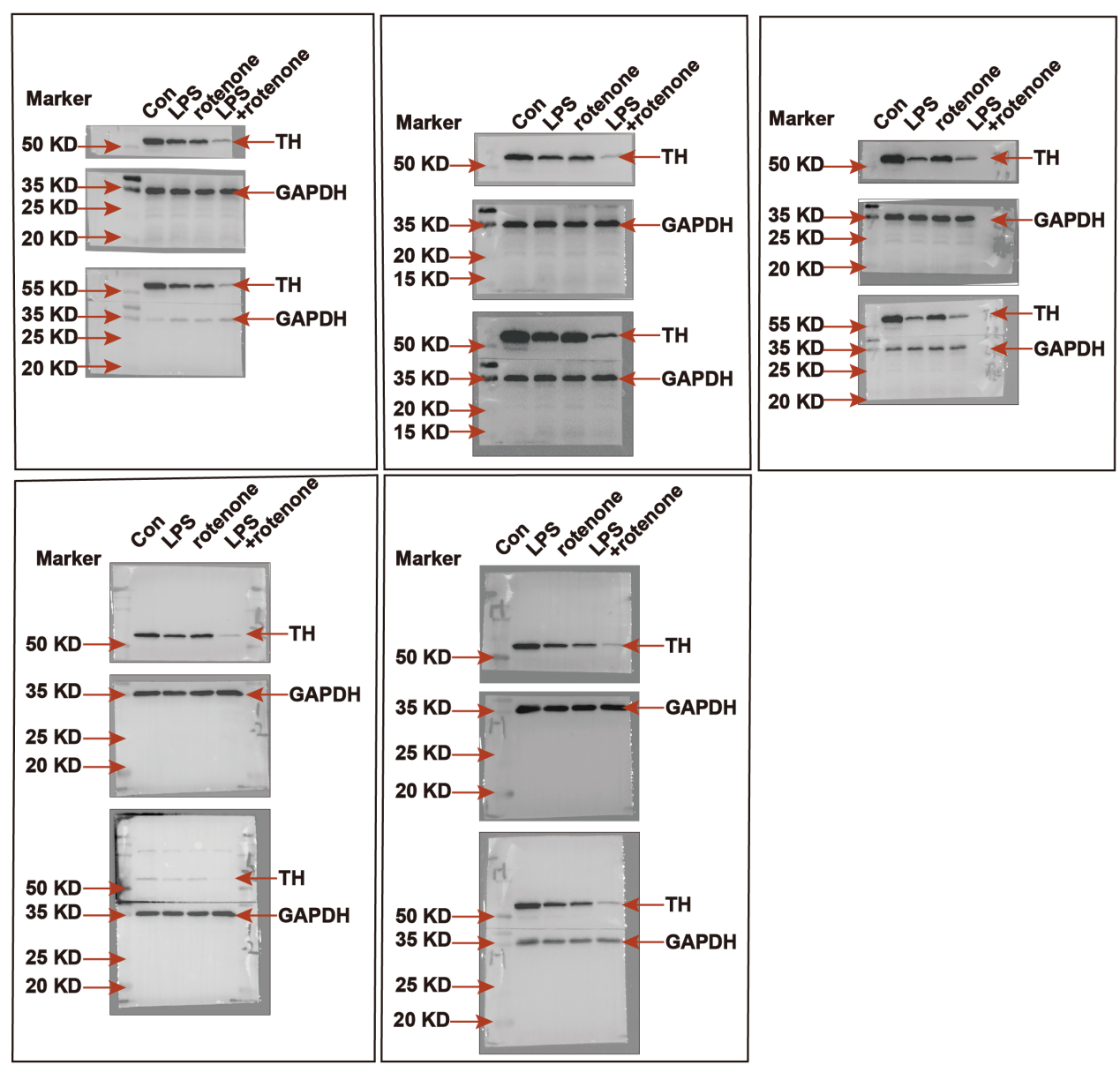


Fig. S3J ASC


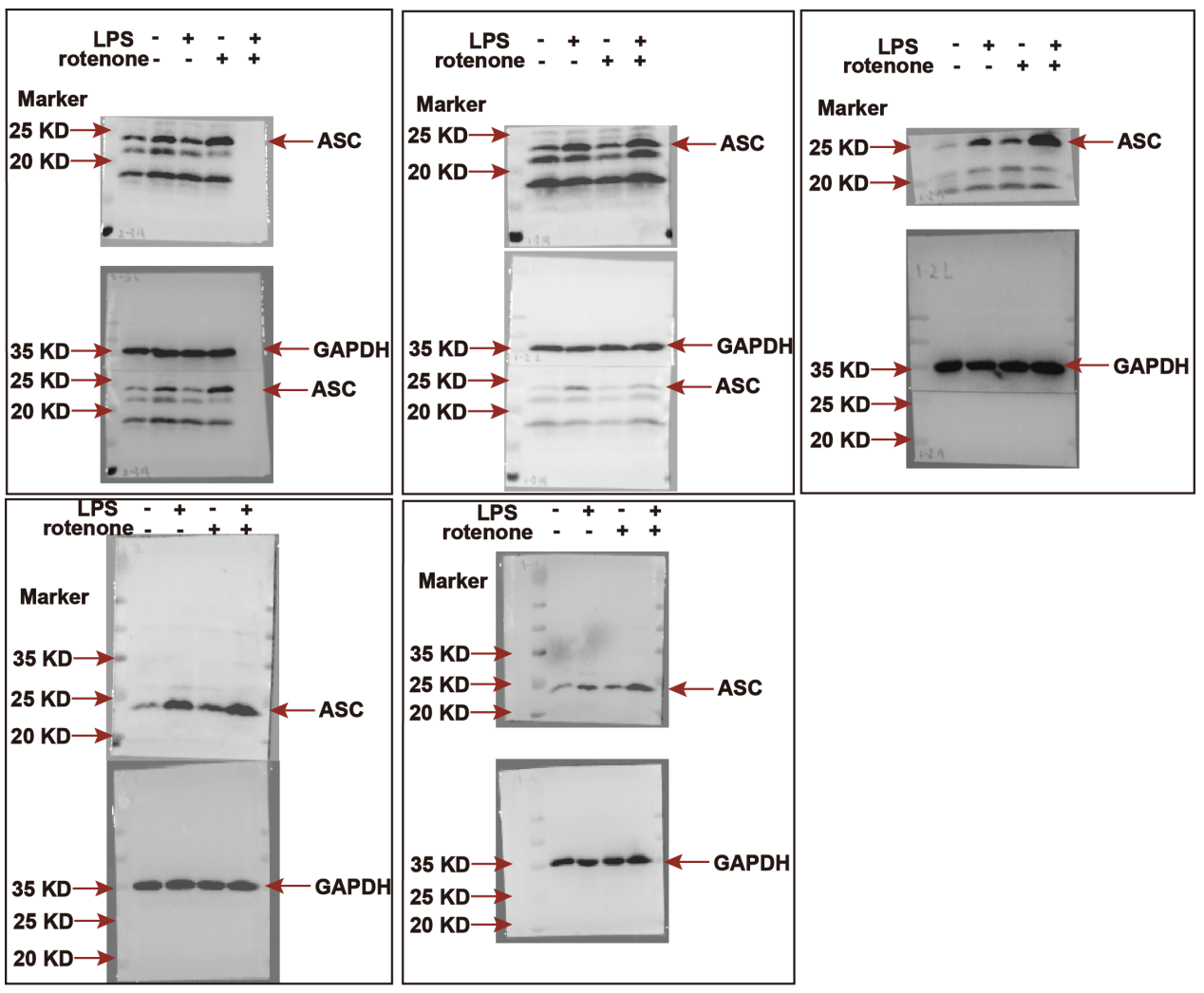


Fig. S3J pro-caspase-1


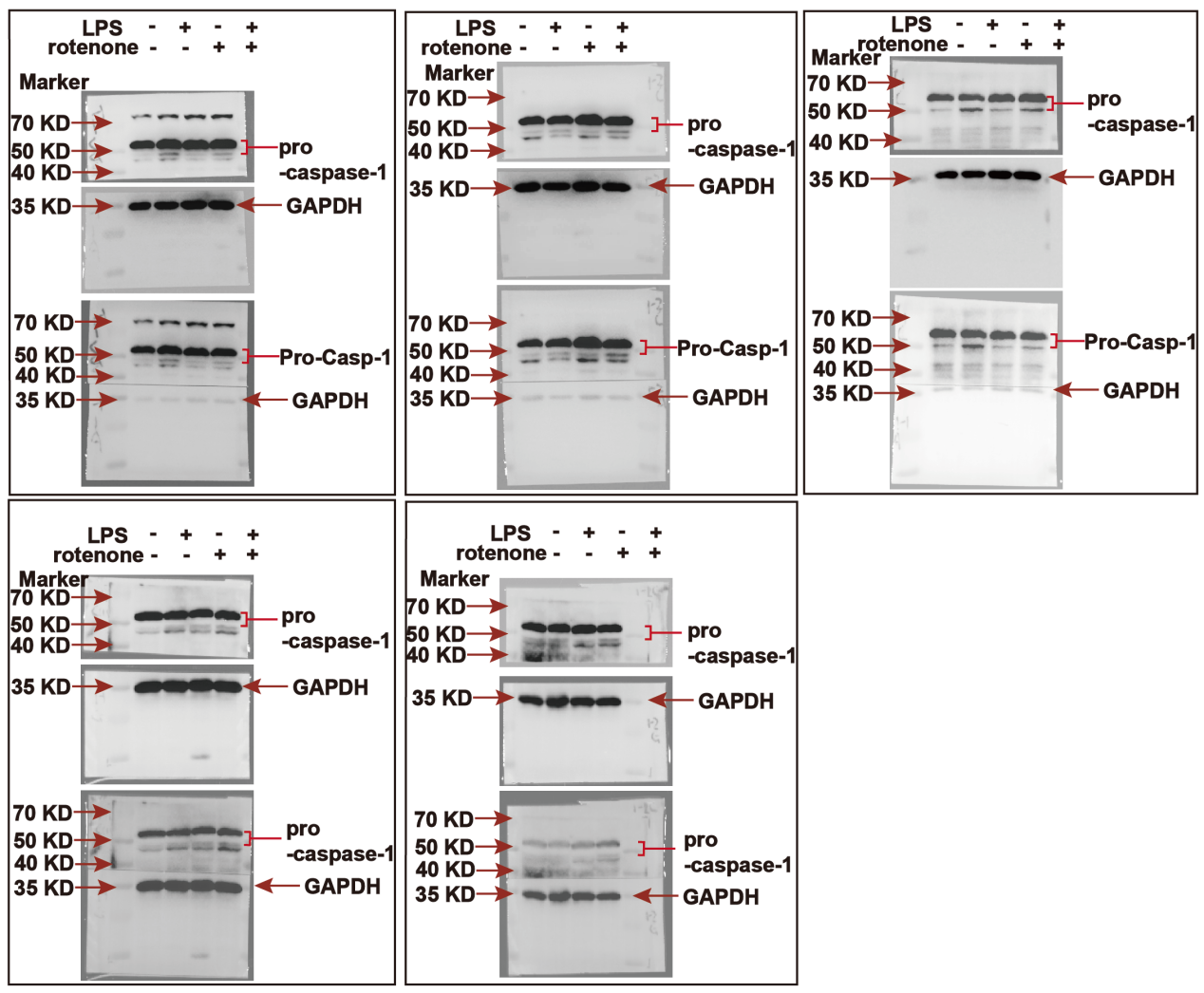


Fig. S3J pro-IL-1β


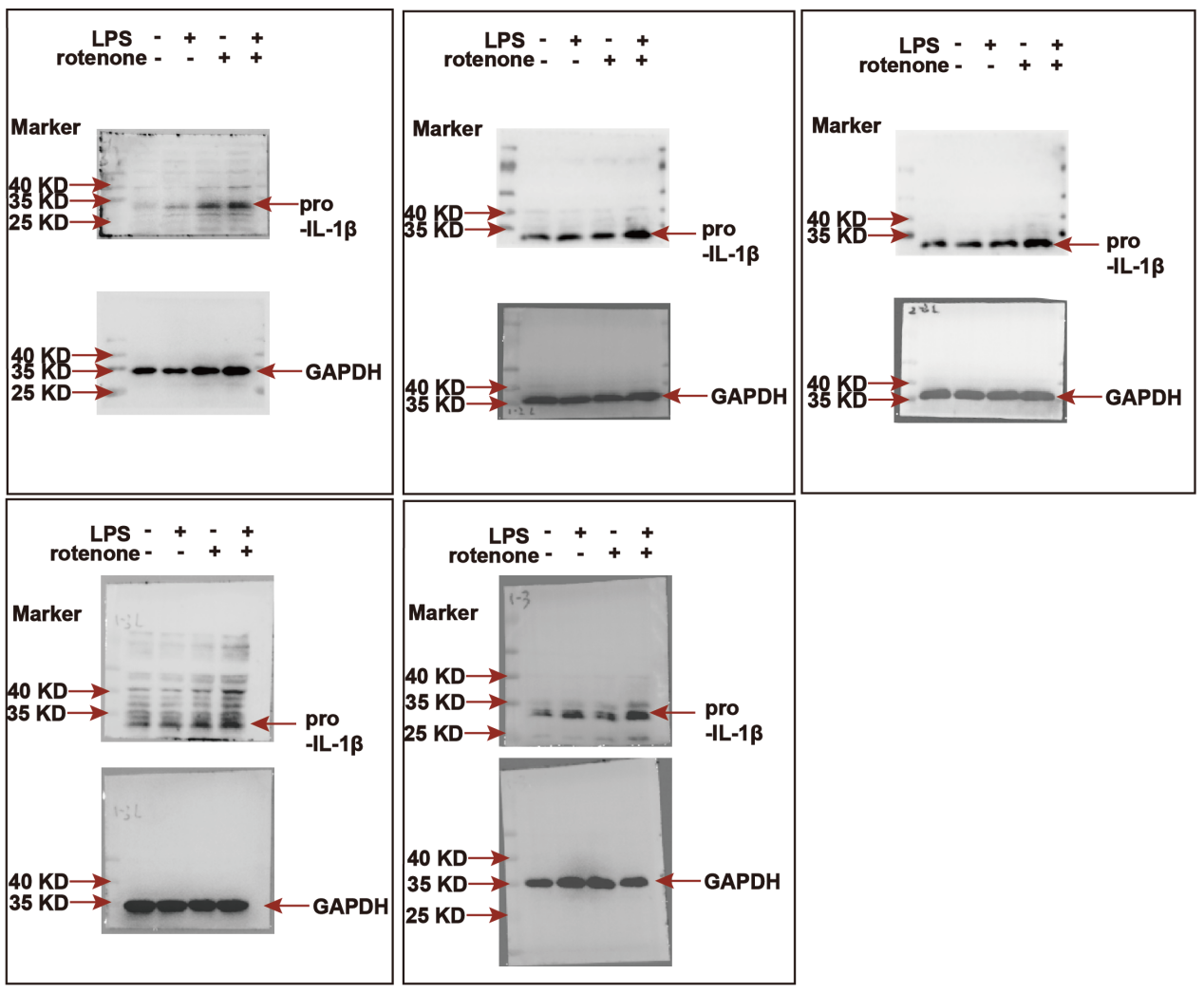


Fig. S4A ASC


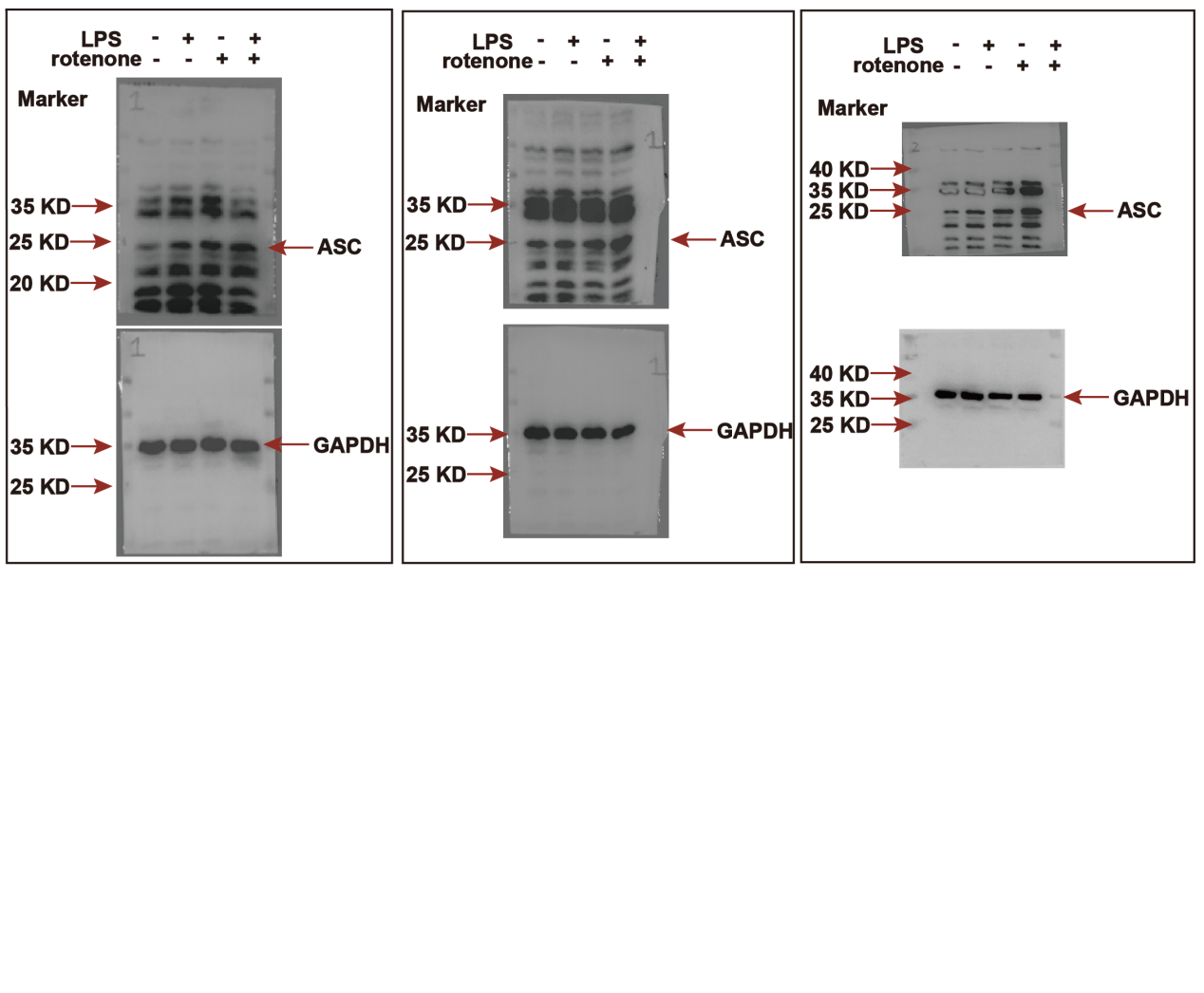


Fig. S4A pro-caspase-1


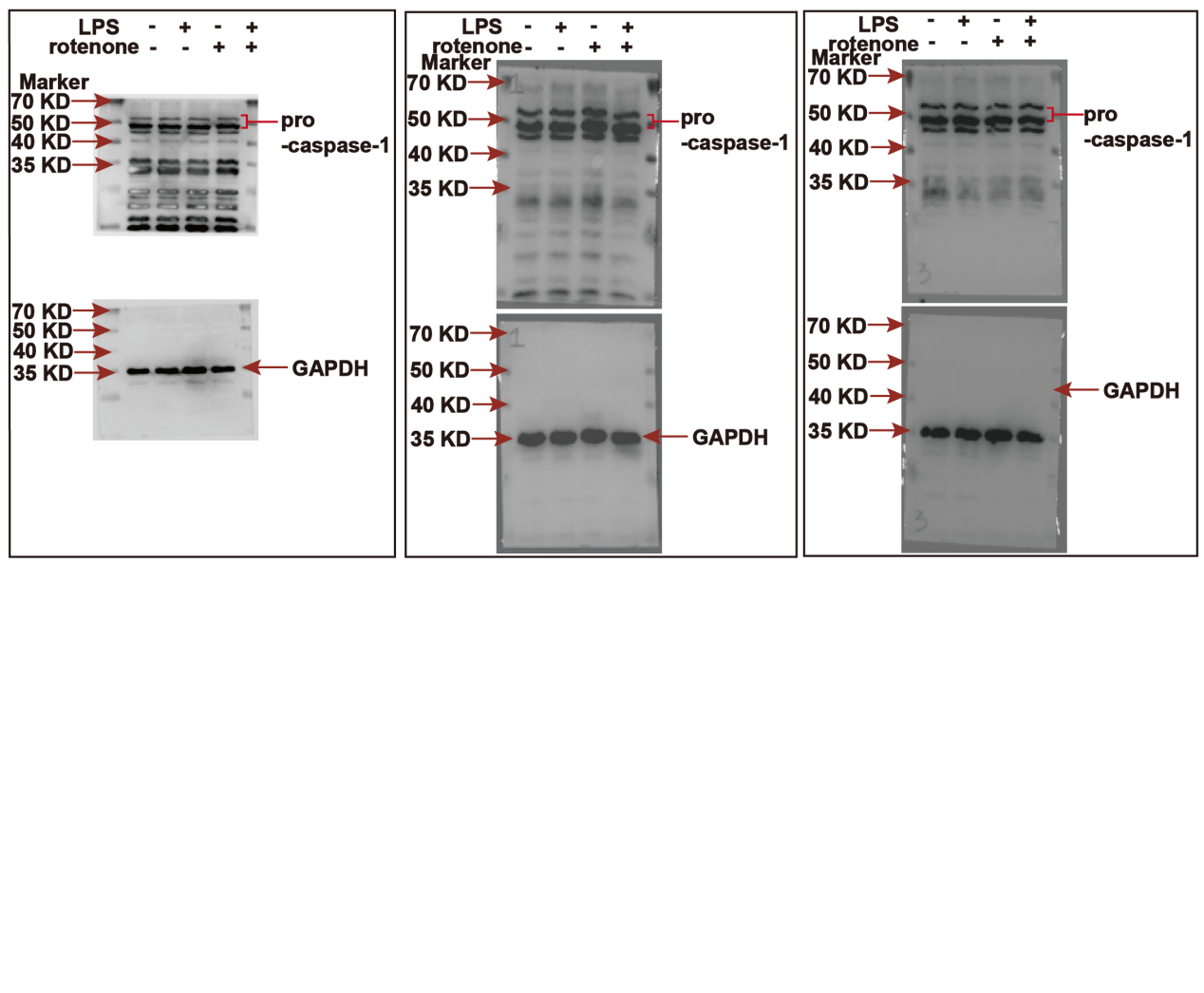


Fig. S4B pro-IL-1β


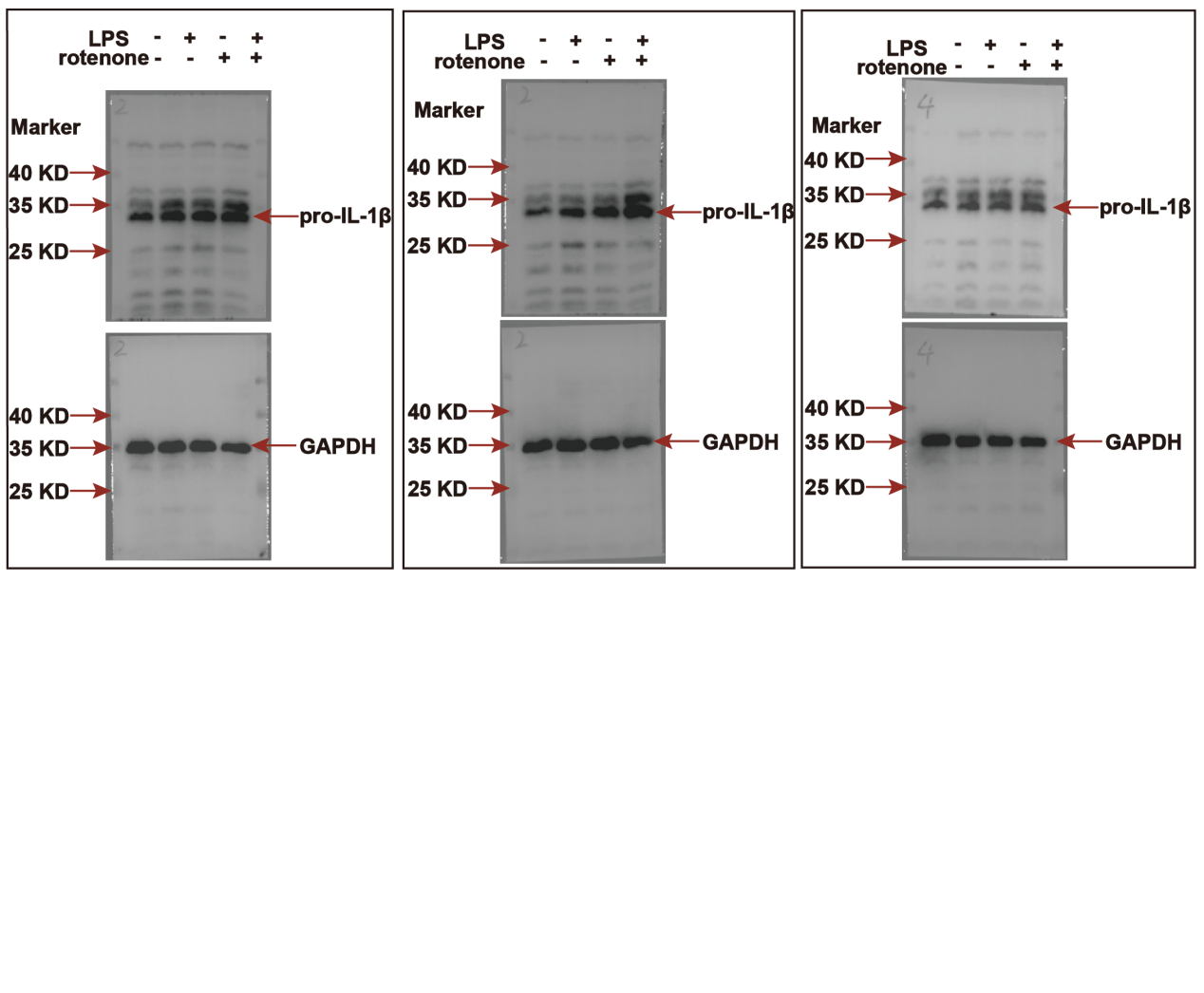


Fig. S4B NLRP3


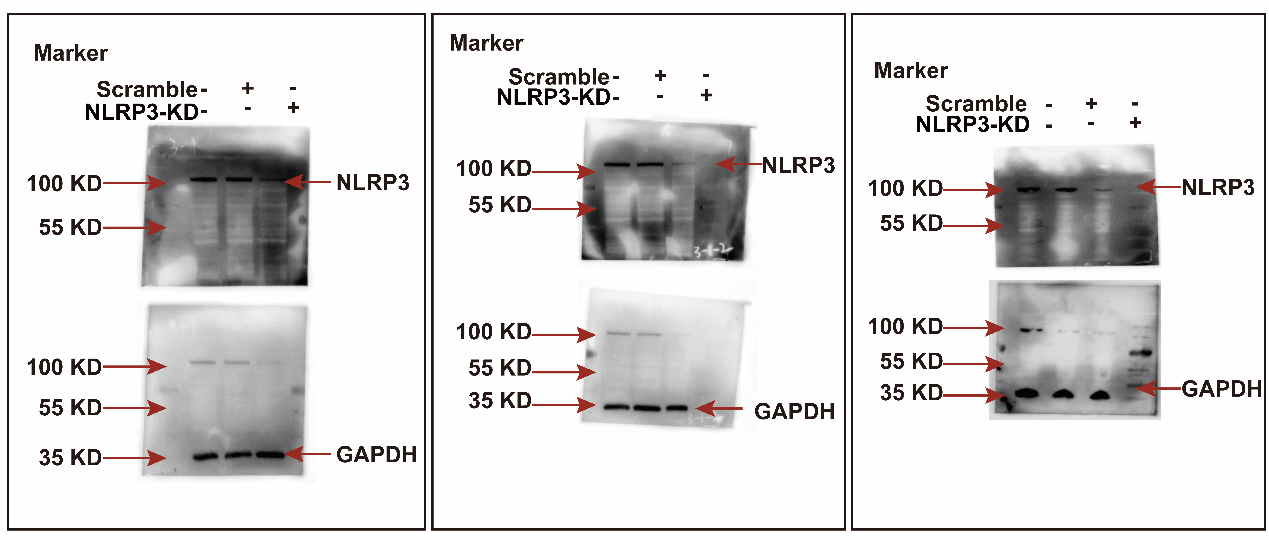


Fig. S5E TH


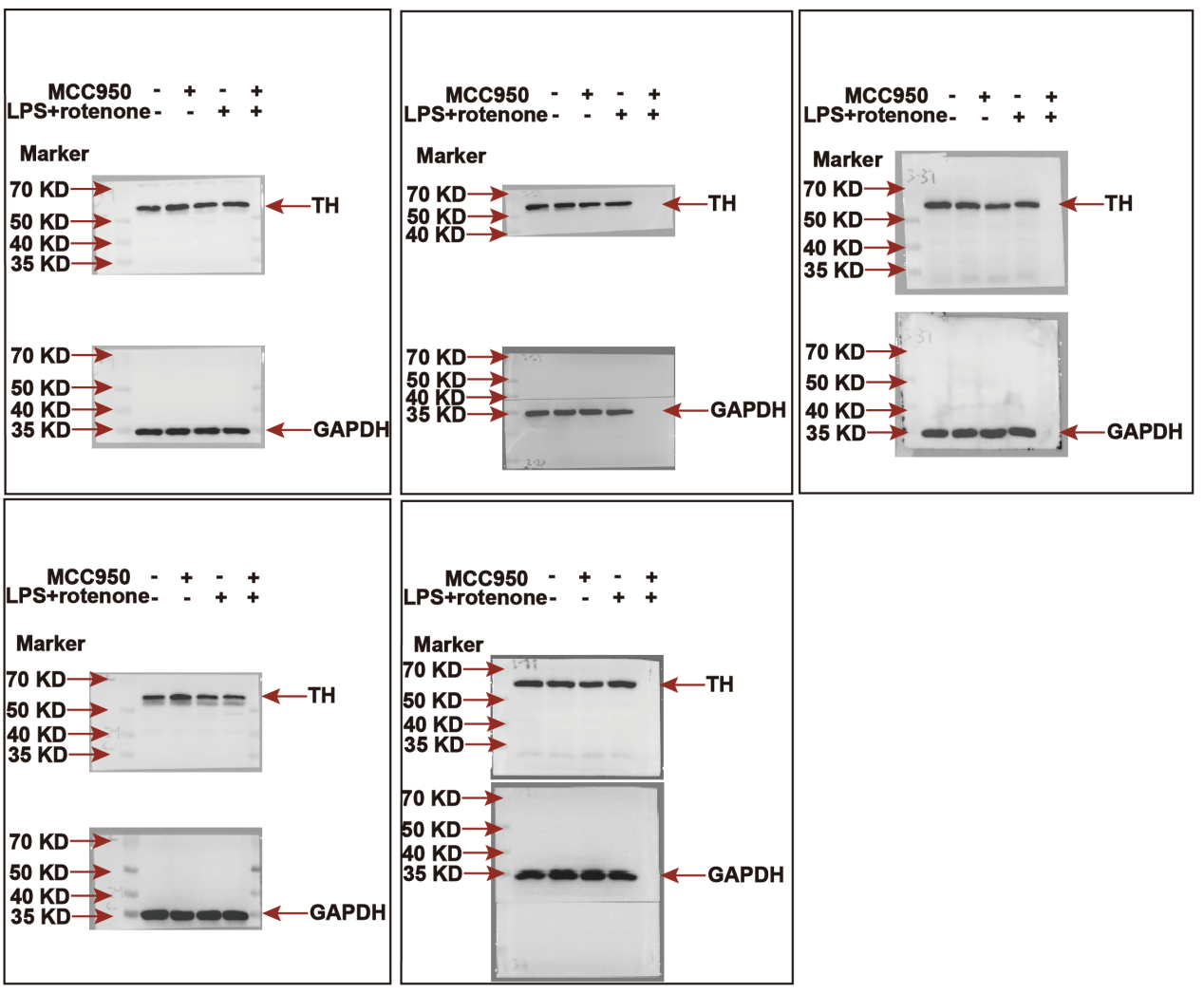


Fig. S5E Iba-1


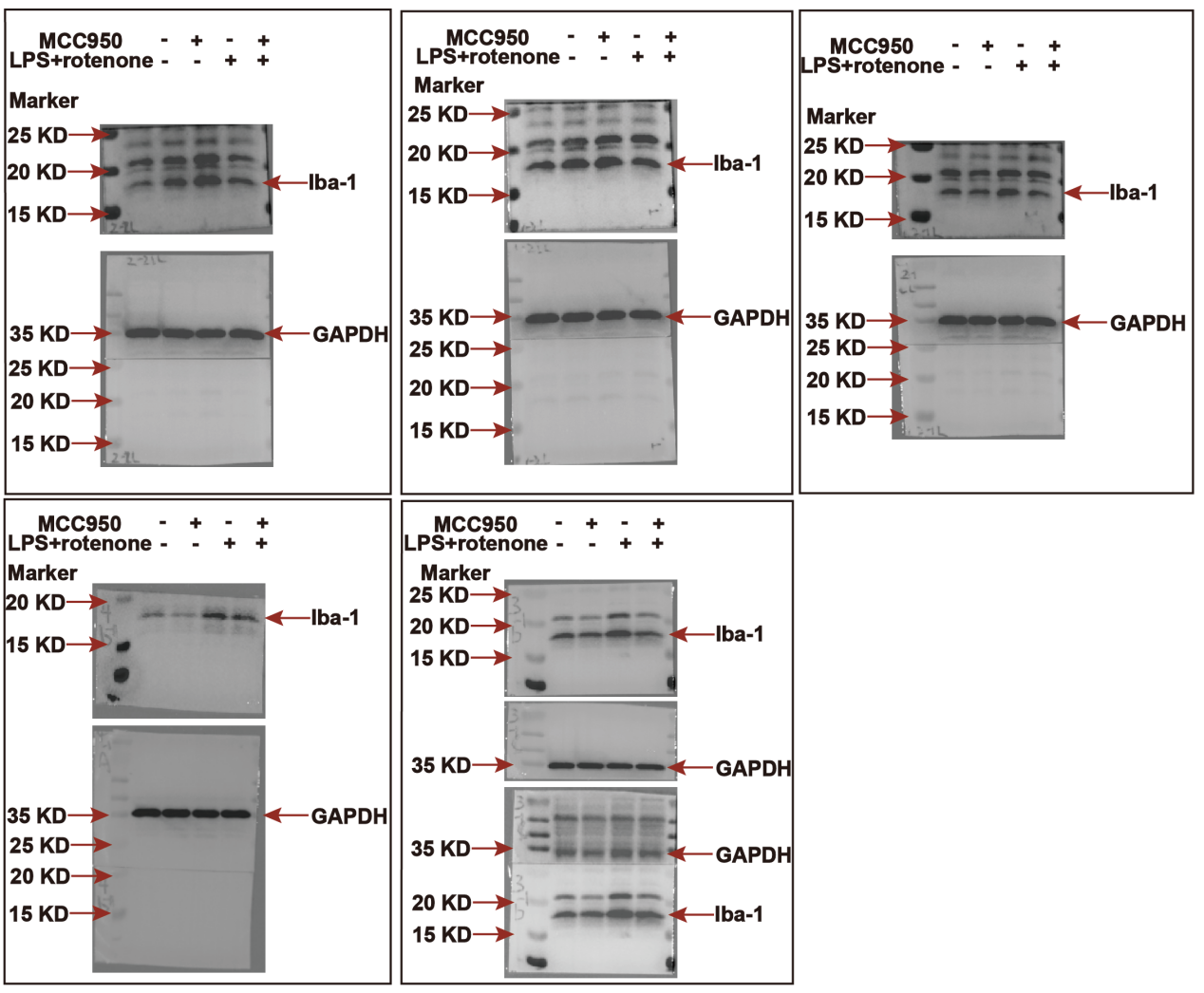


Fig. S5E ASC


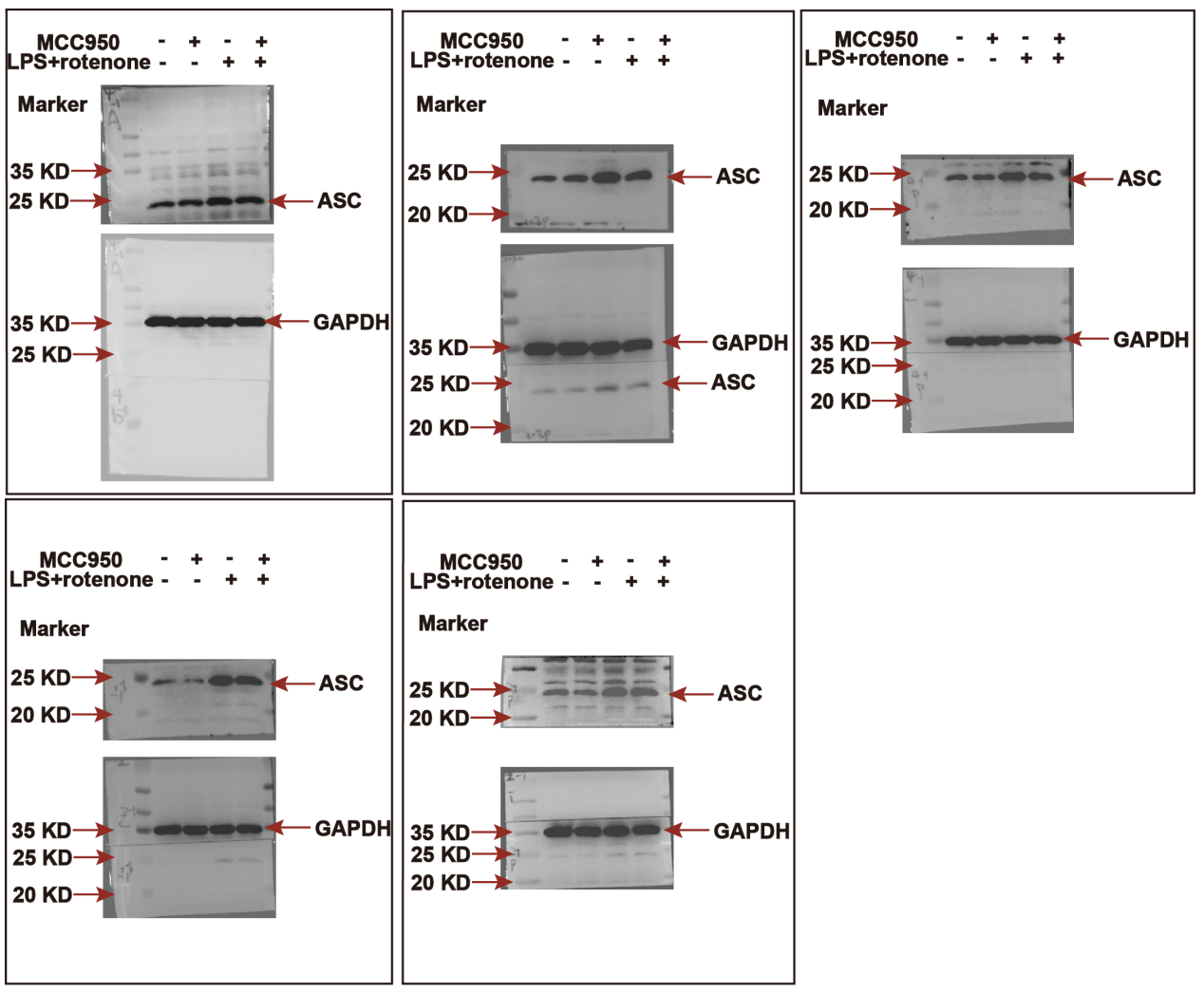


Fig. S5E pro-caspase-1


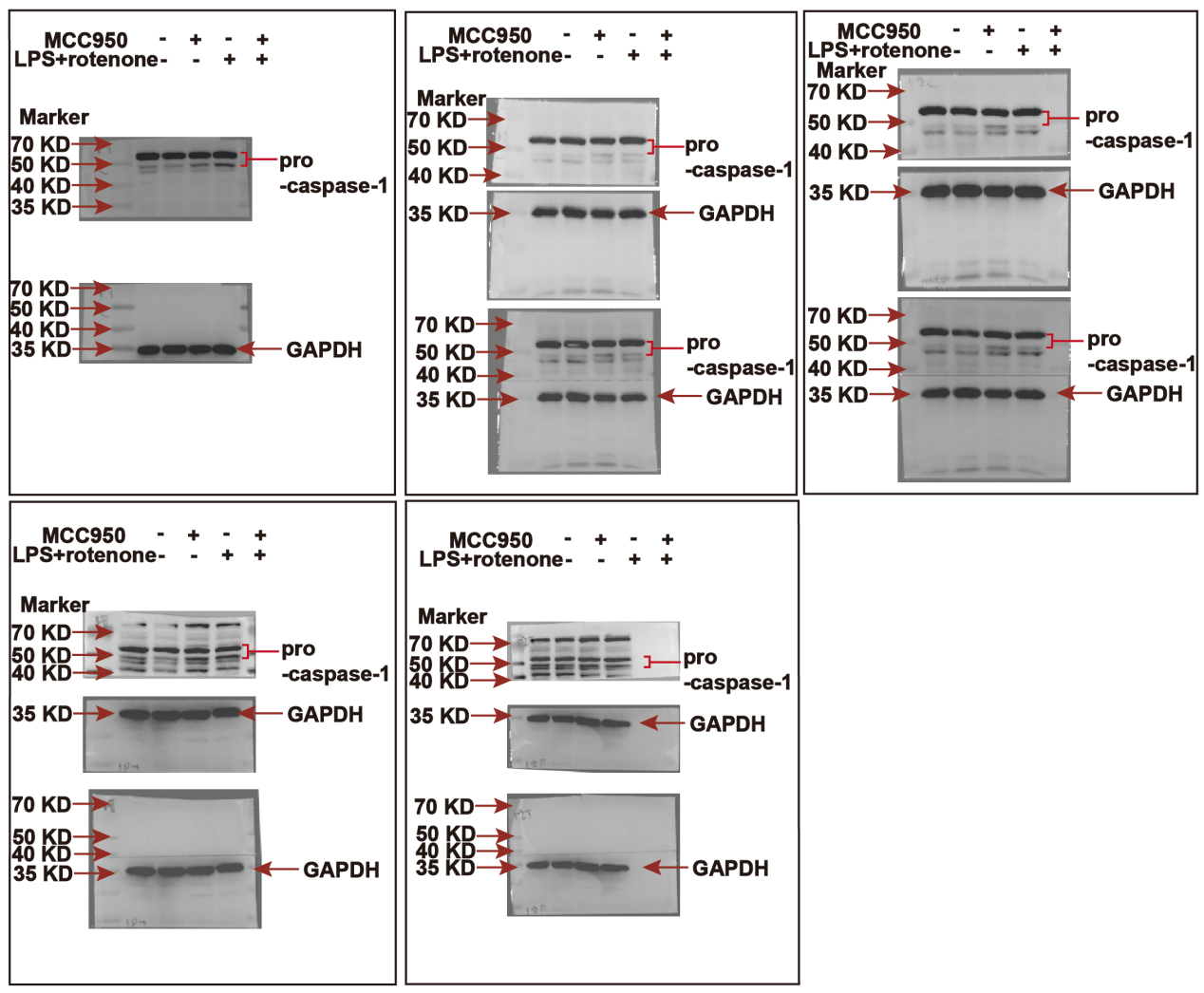


Fig. S5E cleaved caspase-1


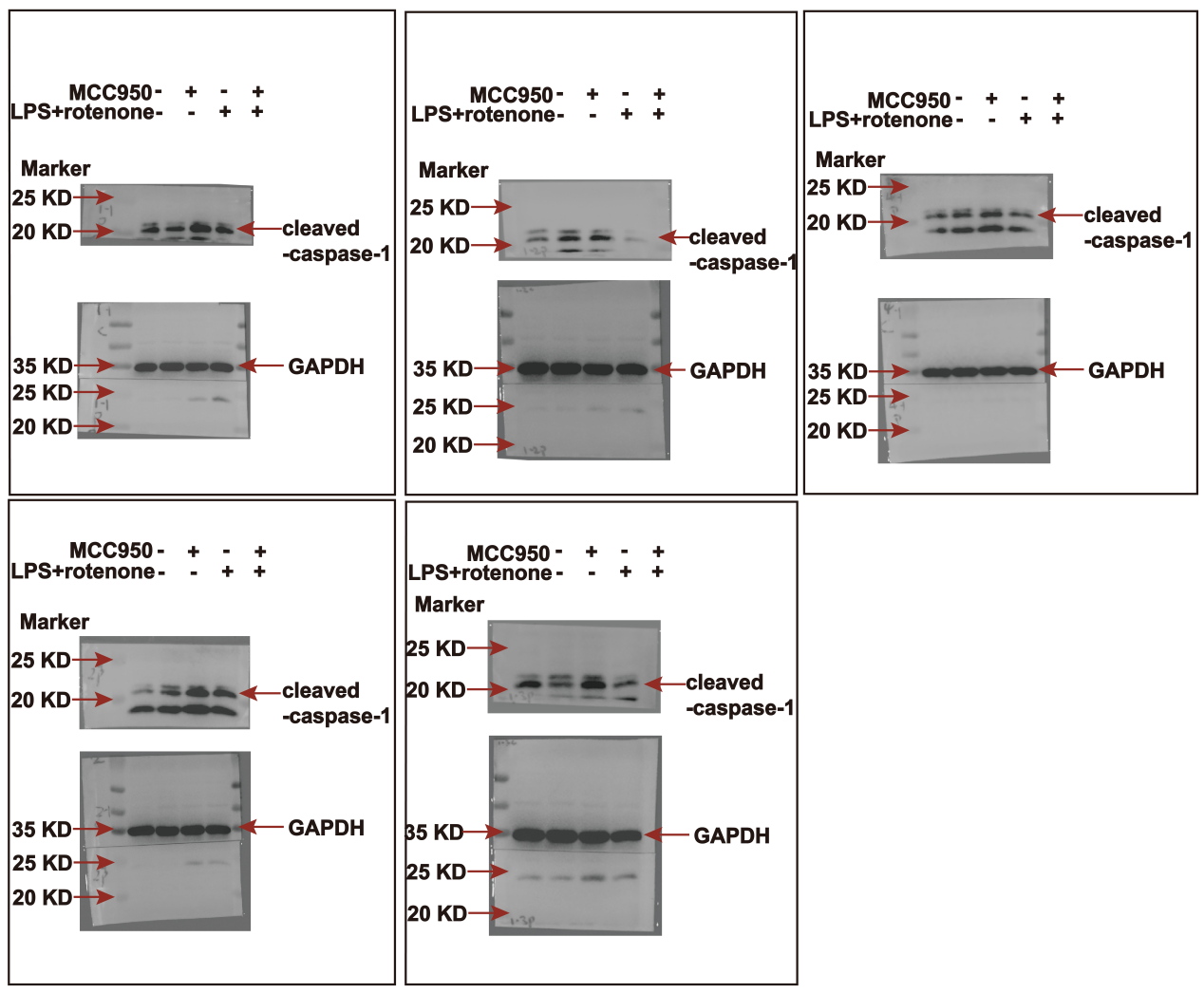


Fig. S5E pro-IL-1β


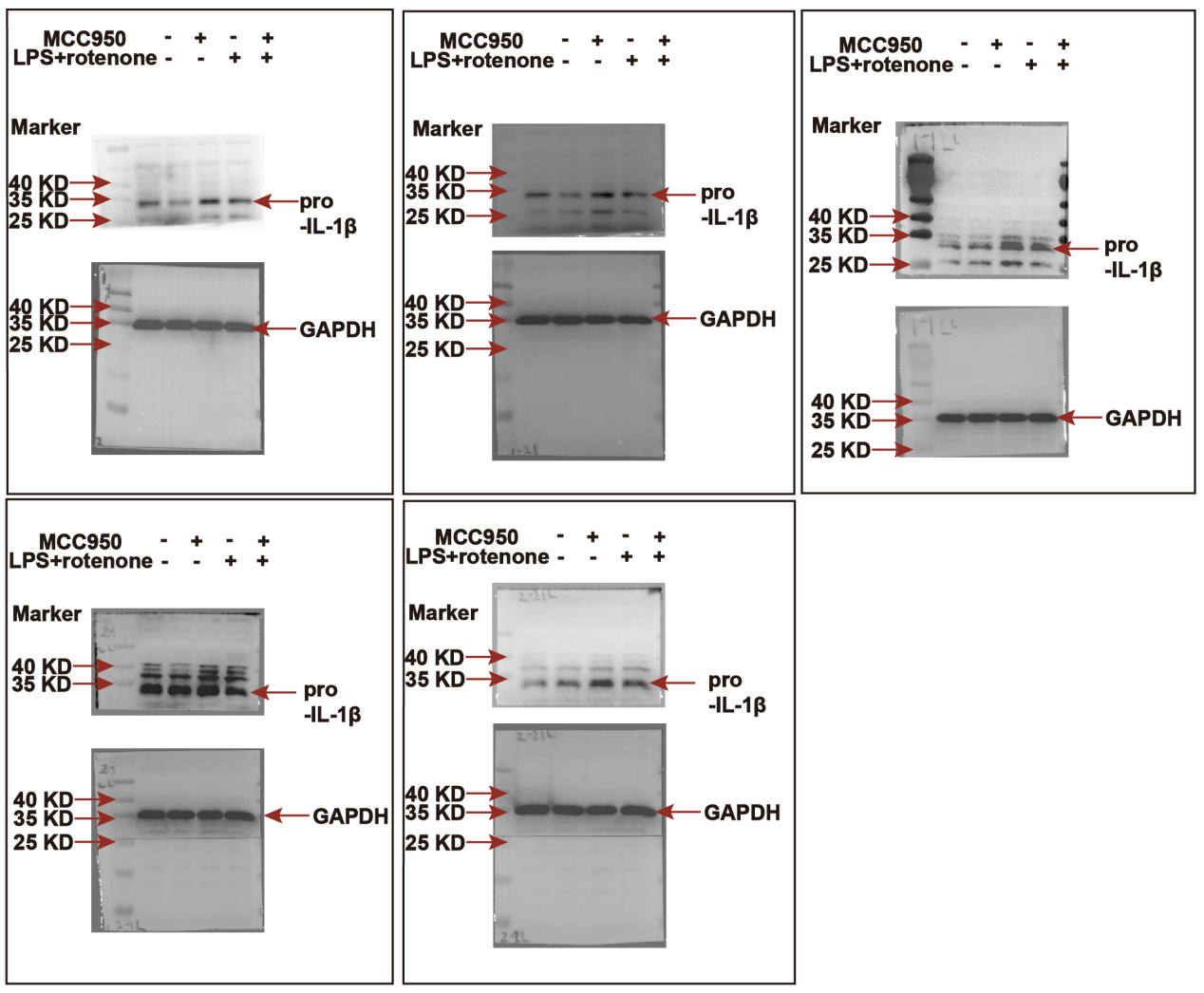


Fig. S5E cleaved-IL-1β


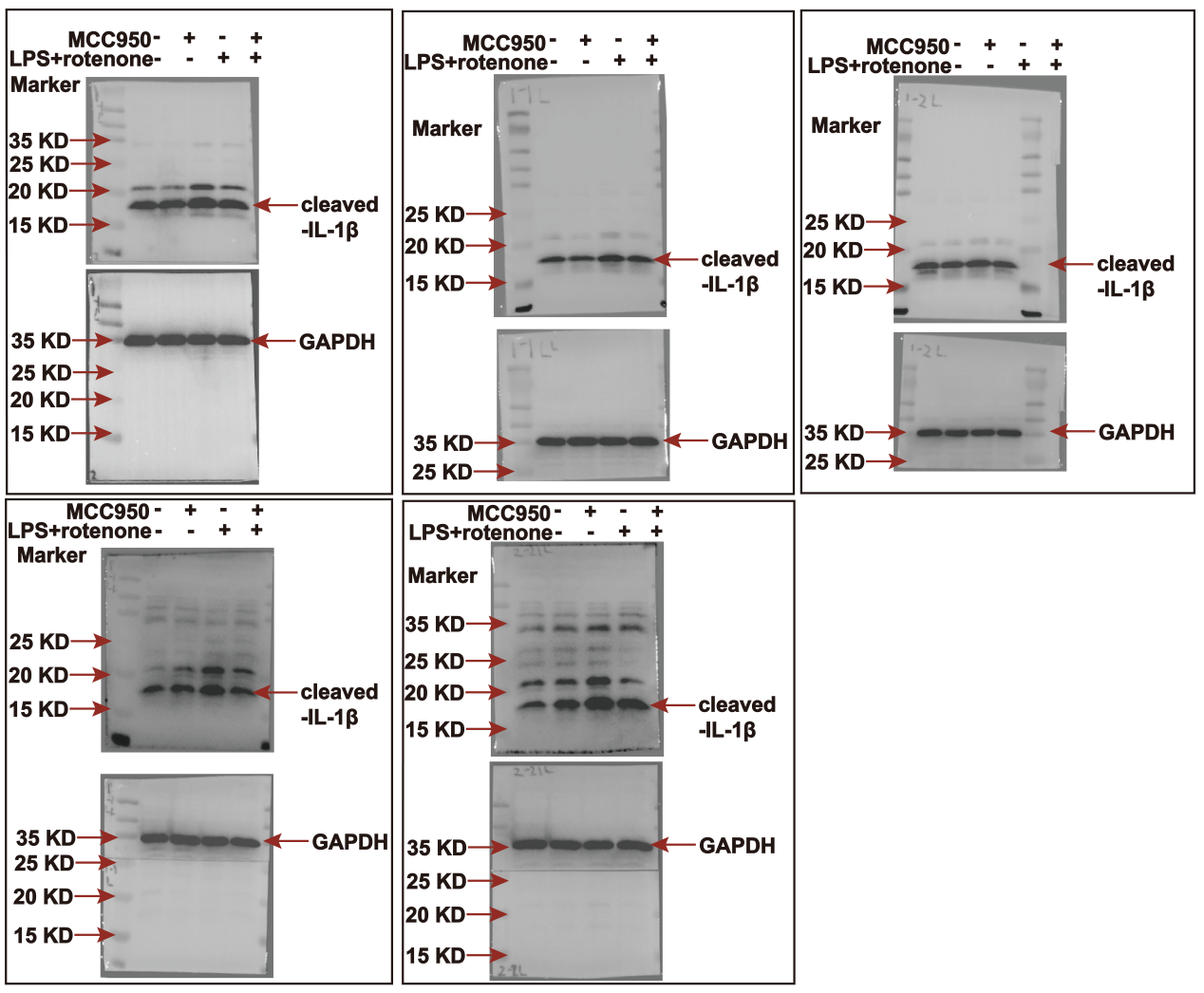


Fig. S5M TH


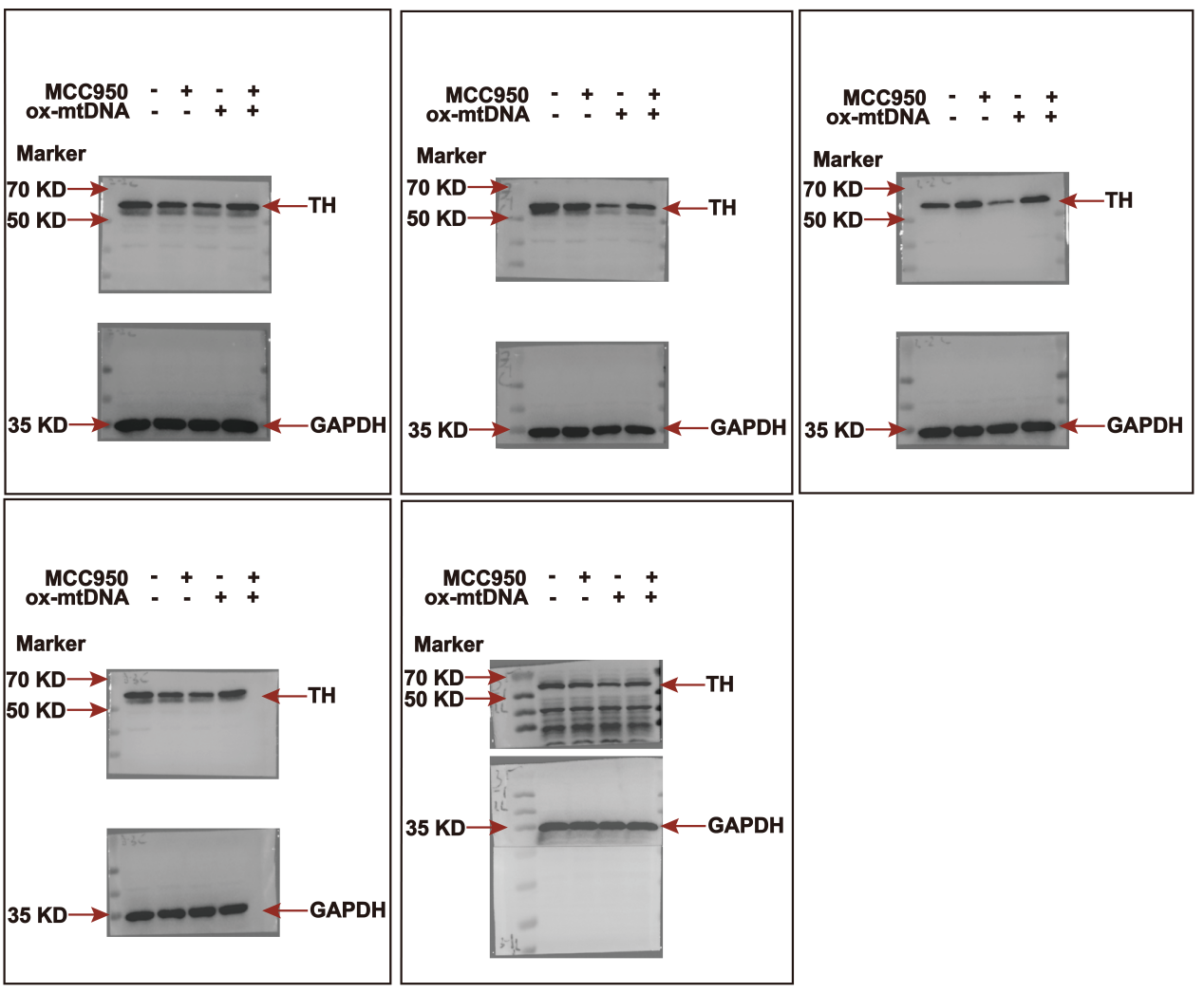


Fig. S5M Iba-1


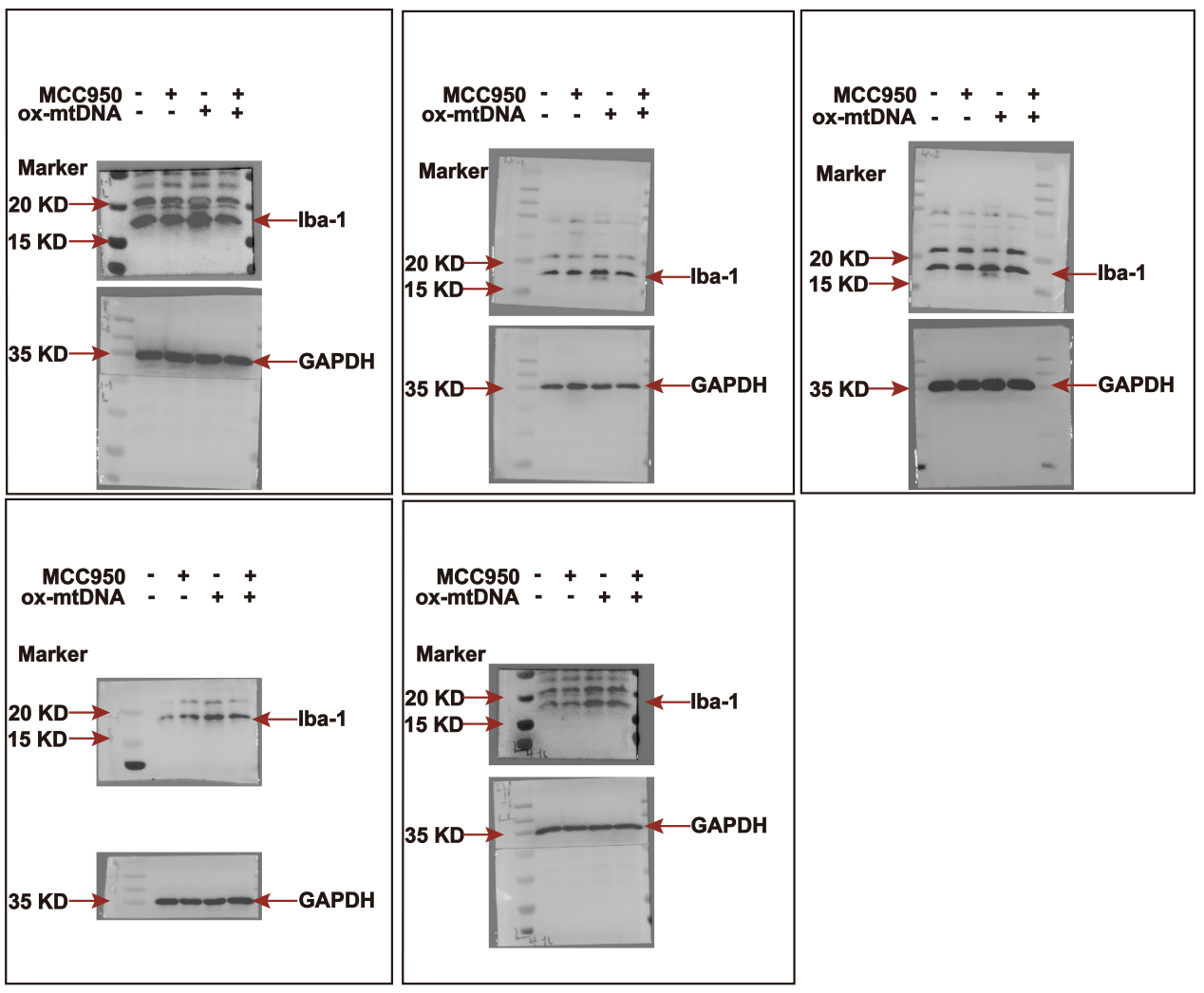


Fig. S5M ASC


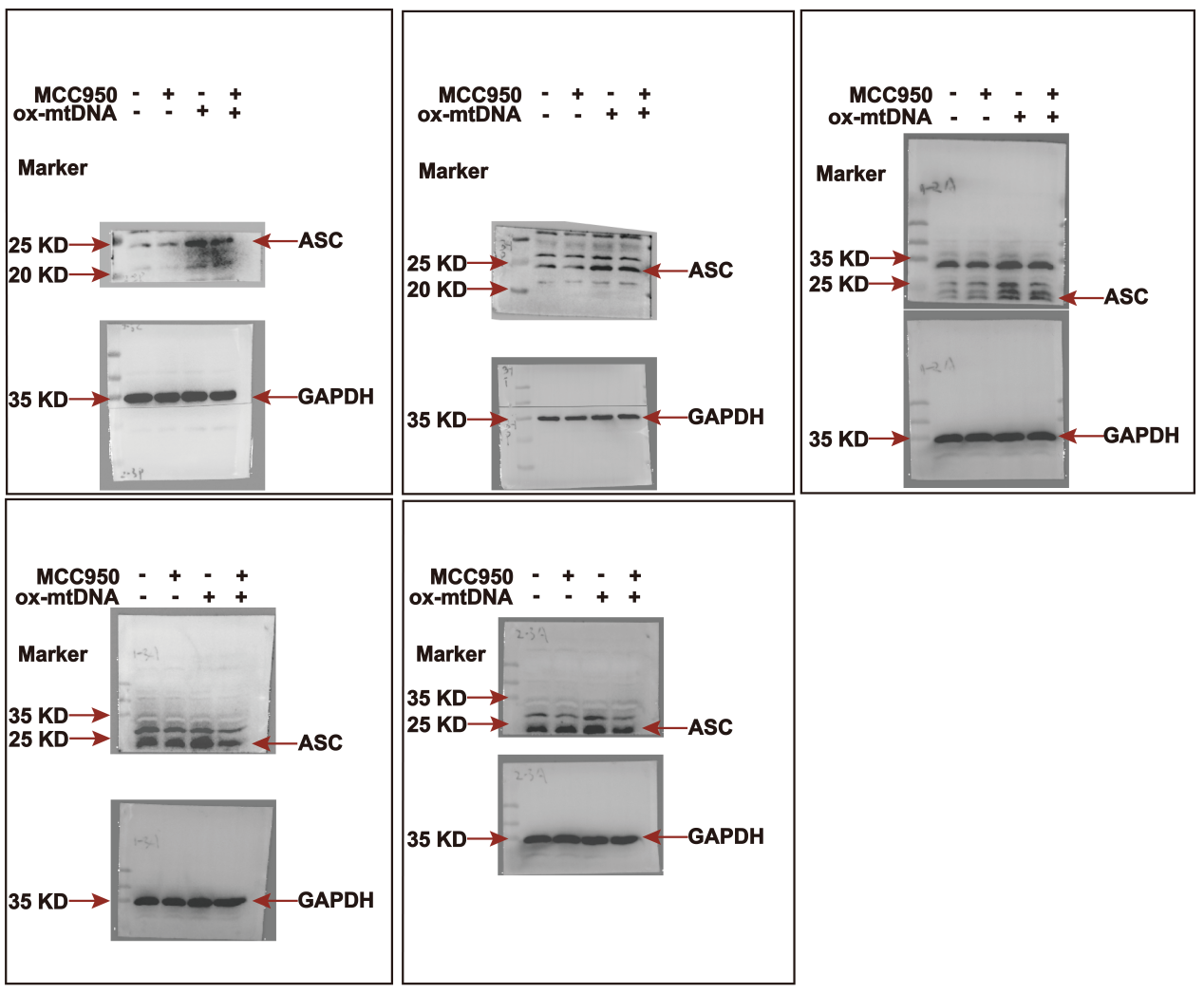


Fig. S5M pro-caspase-1


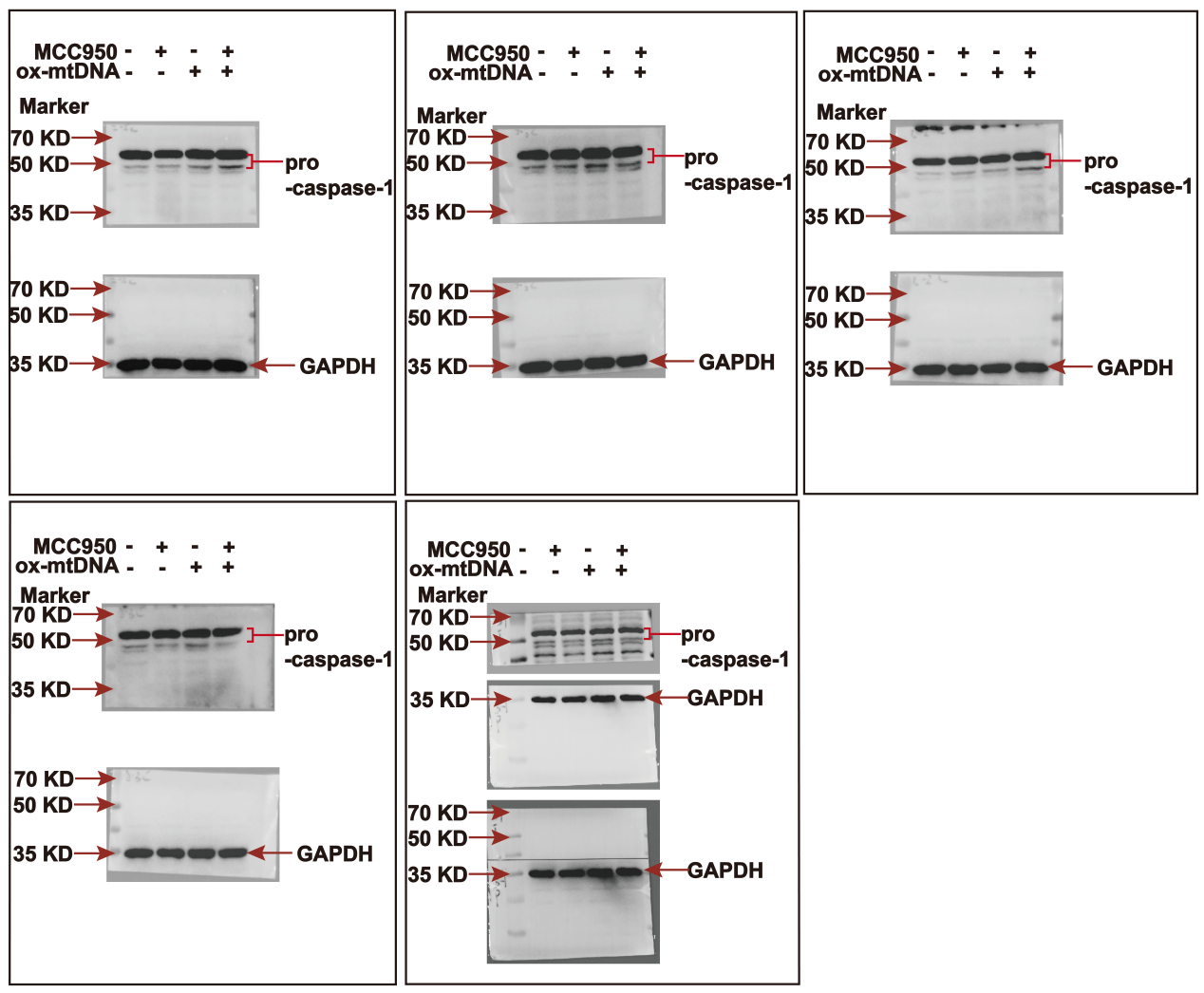


Fig. S5M cleaved caspase-1


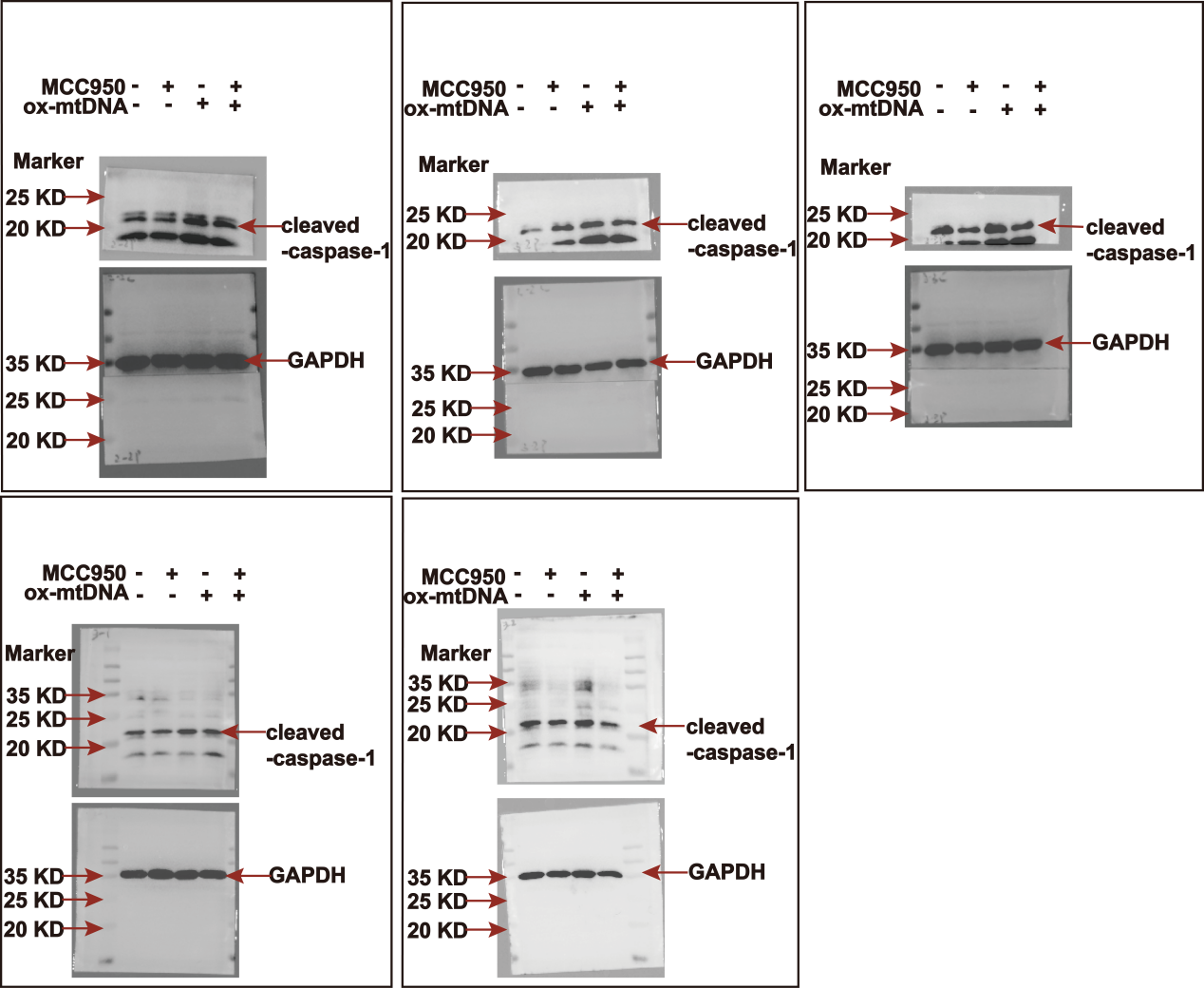


Fig. S5M pro-IL-1β


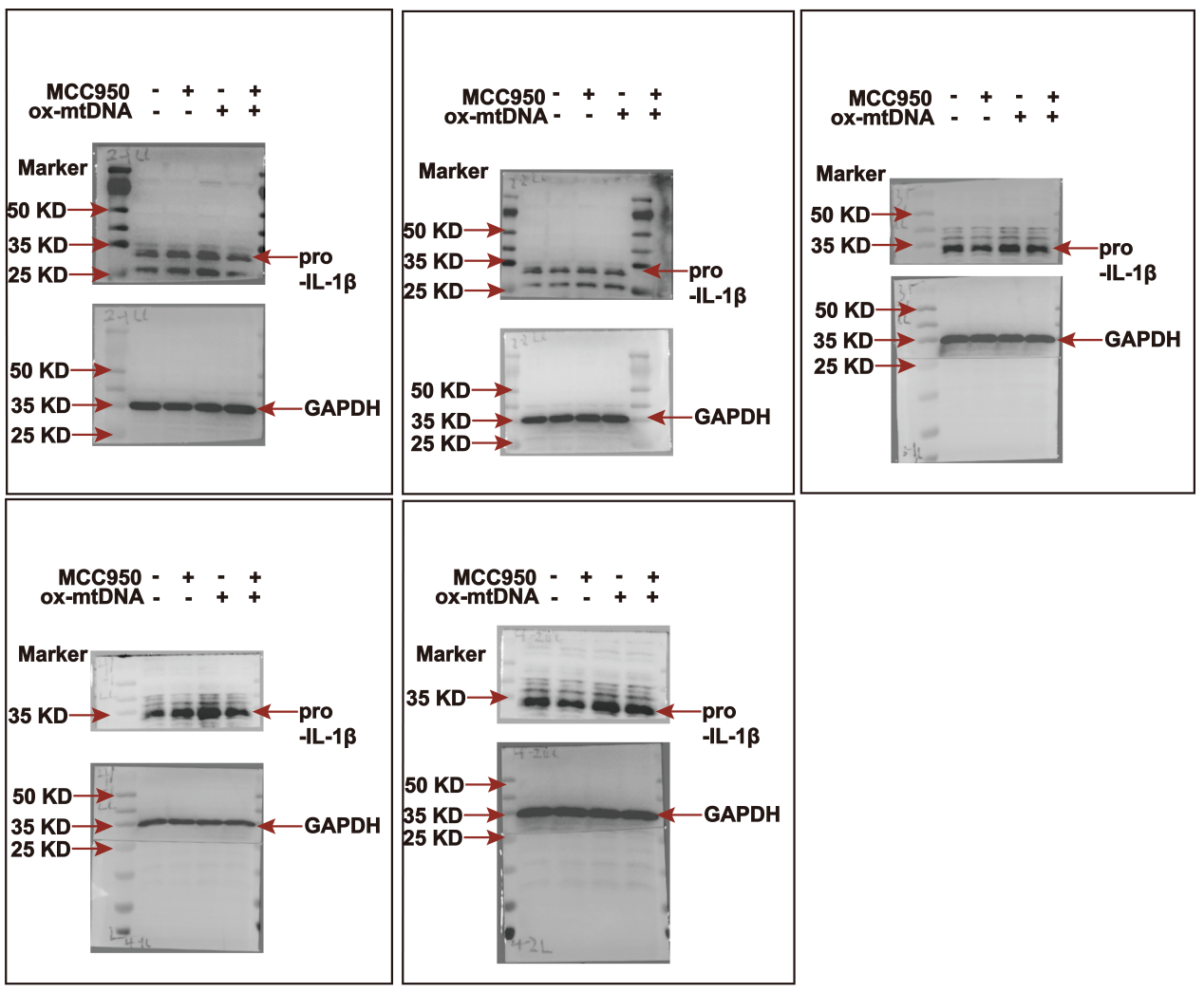


Fig. S5M cleaved-IL-1β


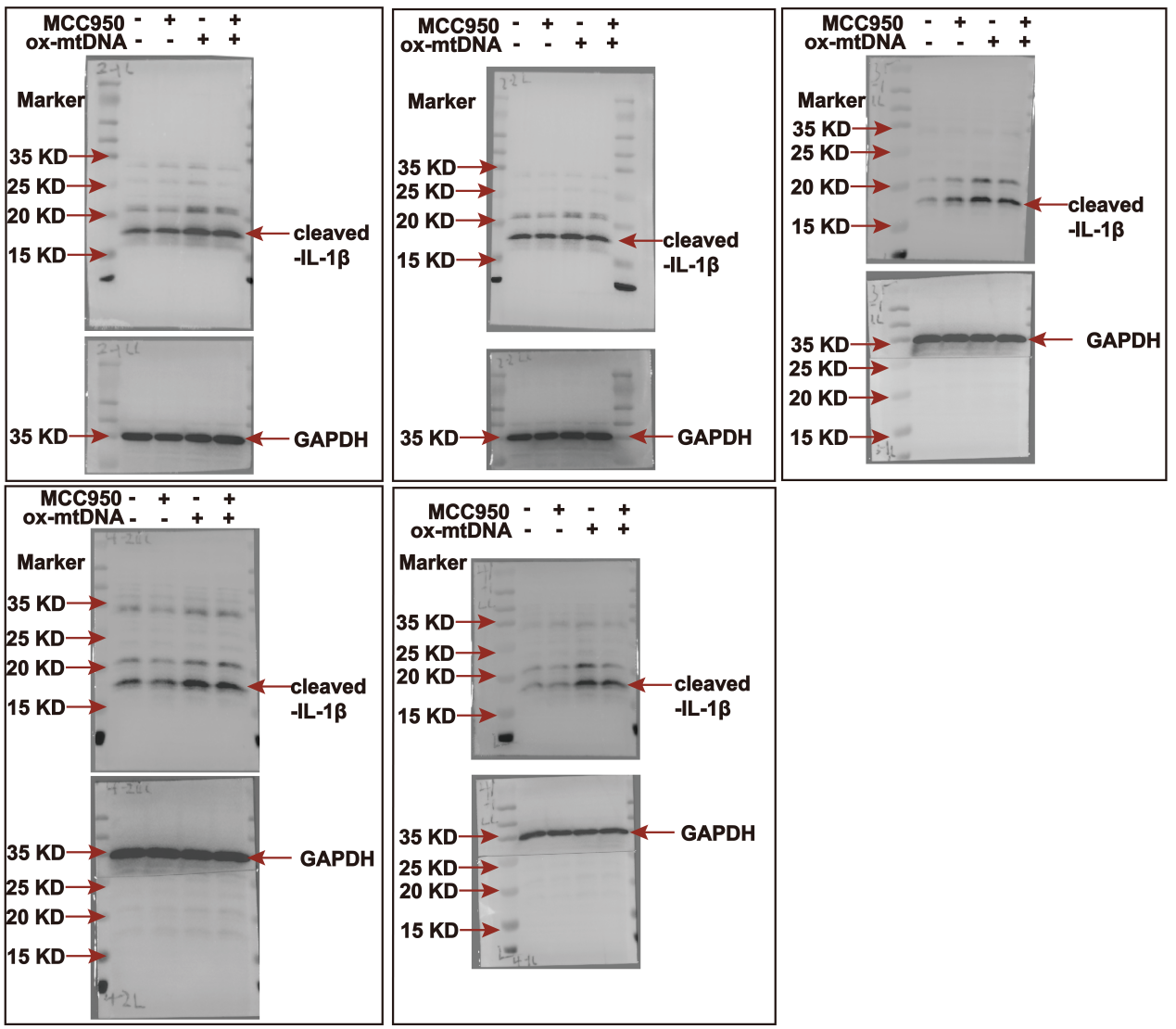


Fig. S7F cleaved-caspase-1


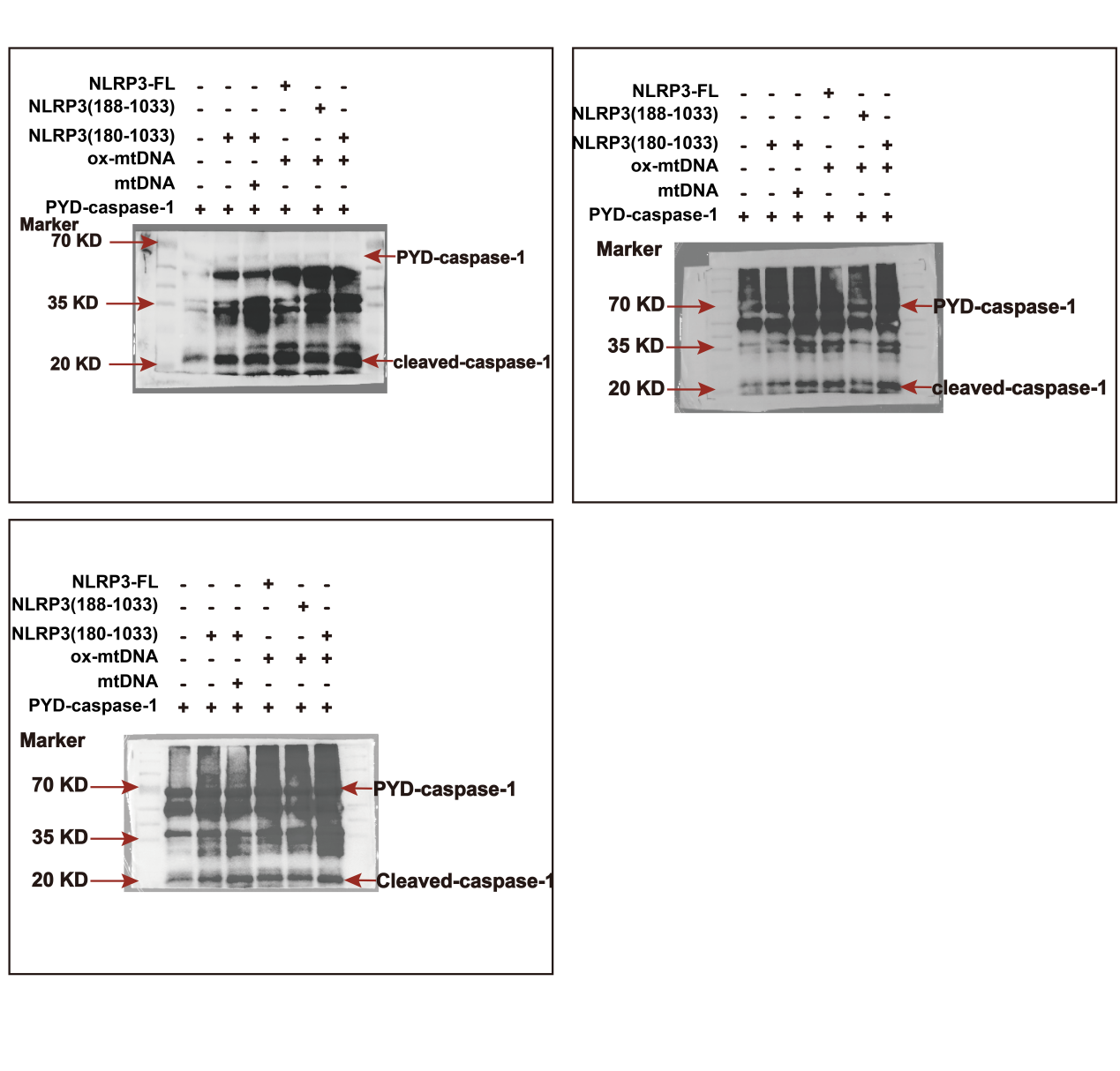

Supplement: Supplementary file 3 — Original western blots [file 41419_2026_8424_MOESM3_ESM.docx]
